# Identification of regenerative roadblocks via repeat deployment of limb regeneration in axolotls

**DOI:** 10.1038/s41536-017-0034-z

**Published:** 2017-11-06

**Authors:** Donald M. Bryant, Konstantinos Sousounis, Duygu Payzin-Dogru, Sevara Bryant, Aaron Gabriel W. Sandoval, Jose Martinez Fernandez, Rachelle Mariano, Rachel Oshiro, Alan Y. Wong, Nicholas D. Leigh, Kimberly Johnson, Jessica L. Whited

**Affiliations:** 1 Harvard Medical School, the Harvard Stem Cell Institute, and the Department of Orthopedic Surgery, Brigham and Women’s Hospital, 60 Fenwood Rd., 7016D, Boston, MA 02115 USA; 2 The Allen Discovery Center at Tufts University, 200 Boston Ave., Suite 4600, Medford, MA 02155 USA

## Abstract

Axolotl salamanders are powerful models for understanding how regeneration of complex body parts can be achieved, whereas mammals are severely limited in this ability. Factors that promote normal axolotl regeneration can be examined in mammals to determine if they exhibit altered activity in this context. Furthermore, factors prohibiting axolotl regeneration can offer key insight into the mechanisms present in regeneration-incompetent species. We sought to determine if we could experimentally compromise the axolotl’s ability to regenerate limbs and, if so, discover the molecular changes that might underlie their inability to regenerate. We found that repeated limb amputation severely compromised axolotls’ ability to initiate limb regeneration. Using RNA-seq, we observed that a majority of differentially expressed transcripts were hyperactivated in limbs compromised by repeated amputation, suggesting that mis-regulation of these genes antagonizes regeneration. To confirm our findings, we additionally assayed the role of *amphiregulin*, an EGF-like ligand, which is aberrantly upregulated in compromised animals. During normal limb regeneration, *amphiregulin* is expressed by the early wound epidermis, and mis-expressing this factor lead to thickened wound epithelium, delayed initiation of regeneration, and severe regenerative defects. Collectively, our results suggest that repeatedly amputated limbs may undergo a persistent wound healing response, which interferes with their ability to initiate the regenerative program. These findings have important implications for human regenerative medicine.

## Persistent healing blocks salamander limb regeneration

Understanding a roadblock to limb regeneration in salamanders could help develop suitable regenerative therapies for humans. Jessica Whited of the Harvard Stem Cell Institute and colleagues in the US found that repeatedly amputating a limb in Mexican salamanders compromised their normal ability to regenerate it. The team found high levels of a gene encoding a damage-protecting protein, called amphiregulin, at the wound site. Artificially inducing the expression of this gene in salamanders whose limbs were amputated just once also led to slow and poor regeneration. The findings suggest that high levels of amphiregulin induce a persistent wound-healing response that interferes with the salamanders’ ability to initiate regeneration. Pushing organisms with strong regenerative abilities into a non-regenerative state can help researchers understand the factors that limit human regenerative abilities and thus develop suitable therapies.

## Introduction

Humans are capable of a limited degree of regeneration such as liver regeneration, and there is solid evidence that humans can regenerate amputated digit tips during childhood.^1,2^ In contrast, the axolotl is a highly regenerative organism and is capable of faithfully replacing an entire appendage following amputation throughout its lifetime. This regenerative process consists of several steps and involves the interplay of many different tissues.^3^ Following limb amputation, the wound is quickly sealed by a blood clot. Within a day, a specialized sheet of epithelium referred to as the wound epidermis encompasses the amputation plane. Following innervation of the wound epidermis, cells within the underlying stump tissue are cued to activate and proliferate to form a critical structure known as the blastema. The molecular and cellular factors driving these actions remain poorly understood. The blastema is a pool of activated progenitor cells that eventually gives rise to new limb tissues; progenitors are thought to largely be fate-restricted over this process.^4^ The anatomical similarities of the axolotl limb with the human limb and well-defined landmarks of the regenerative process make the axolotl an ideal model organism for understanding the mechanisms of limb regeneration and gaining insights into why mammals lack this ability.

Understanding contexts in which axolotl limb regeneration does not proceed normally may provide insight into factors that impact successful regeneration. A classic example of such a context is the reliance of limb regeneration on nerves. Nearly two centuries of research have demonstrated that destruction of the nerves supplying the salamander limb prior to amputation imposes a regenerative block on the limb.^5,6^ When denervated prior to amputation salamander limbs do not form blastemas, and the amputation surface typically heals with an accompanying deposition of fibrotic tissue.^7,8^ More recently, a study showed that depletion of macrophages prior to, or during the early stages of regeneration, also results in regenerative failure with some evidence of internal fibrosis, highlighting a requirement for macrophages in limb regeneration and hinting at a possible connection to wound healing.^9^ Similar to denervation and macrophage depletion, failure to form a proper wound epithelium following amputation antagonizes regeneration. Insertion of an amputated limb immediately into the coelom to prevent the formation of a proper wound epithelium or suturing a full thickness skin flap over the amputation site impairs limb regeneration in newts.^10,11^ Interestingly, allowing a proper wound epidermis to form prior to the insertion of the limb into the coelom results in markedly better regenerative outcomes.^12^ Collectively, these studies underscore the functional importance of nerves, macrophages, and wound epithelium for limb regeneration.

The experiments described above demonstrate that manipulating specific systems can block limb regeneration. However, can salamanders perfectly regenerate anatomical structures an unlimited amount of times if no experimental manipulations are made outside of injury? Of course, the answer to this question appears to vary quite dramatically depending on a range of variables including species, type of regenerative process (e.g., lens, limb, heart, etc.), metamorphic state, age, and type of injury (e.g., amputation, bite injury, etc.).^13–17^ Extensive regenerative capacity is well demonstrated in studies of newt lenses, as serial removal of the lens within *Cynops pyrrhogaster* resulted in regeneration of the lens following as many as 18 removals over the course of 16 years.^14^ In contrast, repeat amputation of the limbs of the newt *Notophthalmus viridescens* resulted in severe defects,^18^ though some evidence of successful repeated regeneration in very young newts exists from Spallanzani’s work.^19^ In a recent study, we challenged limb buds to repeated removal and asked if animals could generate normal limbs much later in life than when they are programmed to originally develop.^20^ Intriguingly, this protocol revealed a limitation to first limb development, and animals tasked with developing the limb around 10 months of age (compared to the normal couple of weeks), either did not grow any limb, or they grew a morphologically-normal, miniature limb.^20^ These miniaturized limbs were permanently altered insofar as they regenerated as miniature limbs following amputation, demonstrating that the experiment effectively decoupled appendage size from body size.^20^ Beyond salamanders, the concept of repeated regeneration has been explored in a variety of other contexts. Zebrafish caudal fins have been demonstrated to regenerate over nine amputation-regeneration cycles with the same rate of blastemal growth and final fin size as controls.^21^ However, structural defects within non-regenerate, stump bone were observed,^22^ and repeated amputations were later shown to alter aspects of the proximo-distal patterning along the length of the fin, essentially decoupling tissue growth from pattern in this context.^23^ Regenerative process in many invertebrates is more substantial, and several organisms examined, for example hydra^24^ and planaria,^25,26^ show no evidence of regenerative failure even after many insults. Although the answer to the question of whether regeneration can be repeatedly deployed in a perfect fashion varies between species and organ or appendage, these studies show that pushing the limits of highly-regenerative organisms can greatly advance our understanding of fundamental regenerative principles.

Herein, we tested the extent of the axolotl limb regenerative program by repeatedly amputating their forelimbs and allowing them to complete several regenerative cycles. We found that regenerative fidelity declined, and an increasing number of limbs failed to regenerate beyond the amputation plane when forelimbs were challenged with an increasing number of amputations at the same plane. Moreover, those limbs that failed to regenerate after repeated amputation showed signs indicative of fibrosis. RNA-seq profiling of regeneration-incompetent limbs compared to normally-regenerating limbs highlighted an abnormally high expression of *amphiregulin* during the wound healing stage of limb regeneration, suggesting that persistently high levels of Amphiregulin may antagonize limb regeneration. Consistent with this hypothesis, we found that overexpression of *amphiregulin* in axolotl limbs undergoing one regenerative cycle regenerated more slowly and exhibited significantly poorer regenerative outcomes relative to control limbs. Overall, our study suggests that the decline in regenerative ability of the axolotl limb after repeated amputation can be leveraged to identify novel gene expression patterns that are disruptive to regeneration.

## Results

### Axolotl limbs exhibit a decline in regenerative capabilities when repeatedly amputated at the same plane

**Fig. 1:**
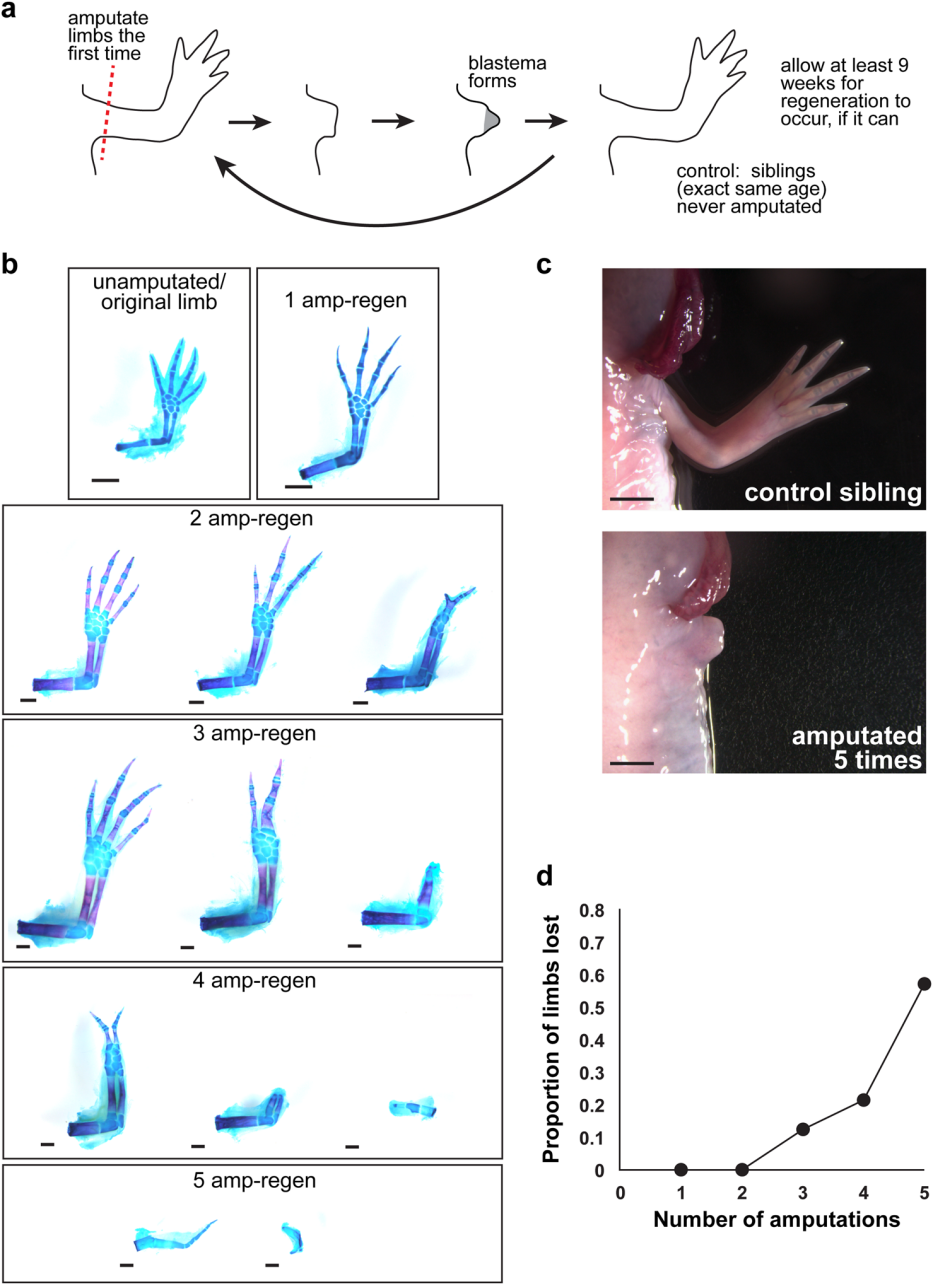
Regenerative decline after repeated amputation. Both forelimbs were amputated and allowed to fully regenerate. **a** Experimental overview. **b** Skeletal preparations of limbs following successive rounds of amputation. The limb in bottom right panel failed to regenerate beyond the plane of amputation (dashed line). **c** Representative examples of sibling control limb (top left panel) and limb that failed to regenerate after repeated amputation (bottom left panel). **d** Cumulative distribution plot of loss in ability to regenerate beyond the plane of amputation (right graph). Scale bars in **b** are 1 mm

To determine whether axolotl limbs are limited in their ability to continually regenerate limbs, we performed proximal amputations on both forelimbs of individually-housed, naïve axolotls (Fig. 1a) that had never experienced bite injury through communal living or experimental amputation. Our first amputation was performed on axolotls ~2 months post-hatching (~3-4 cm snout-to-tail in length), and we allowed limbs to fully regenerate (on average, 13 weeks between each amputation) before amputating limbs in the same plane again (details on how the plane of amputation was identified are provided in Supplementary Fig. 1a–b). We used “digits stage” as a marker of full regeneration. As expected, all of the limbs from this cohort of animals were able to regenerate to the digits stage following a single amputation (Fig. 1b *n* = 32 limbs/16 animals). However, the percentage of limbs that were able to regenerate completely progressively decreased upon repeated amputation to the point where only 25% of limbs challenged with five amputations were able to regenerate digits (Fig. 1b *n* = 28 limbs/14 animals for each time point).

After each round of amputation, we additionally quantified the proportion of limbs that were not able to regenerate beyond the plane of amputation. We found that more than half of the limbs in our study failed to regenerate when challenged with five rounds of amputation (Fig. 1c, d, *n* = 16/28 limbs). These limbs appeared to have no visible external regeneration with no observable blastema, and they exhibited a stump-like morphology. Taken together, these data indicate that axolotl limbs may be finite in their ability to undergo repeated regeneration cycles.

### A shift in the amputation plane leads to less severe regenerative decline in limbs challenged with repeated amputations

**Fig. 2:**
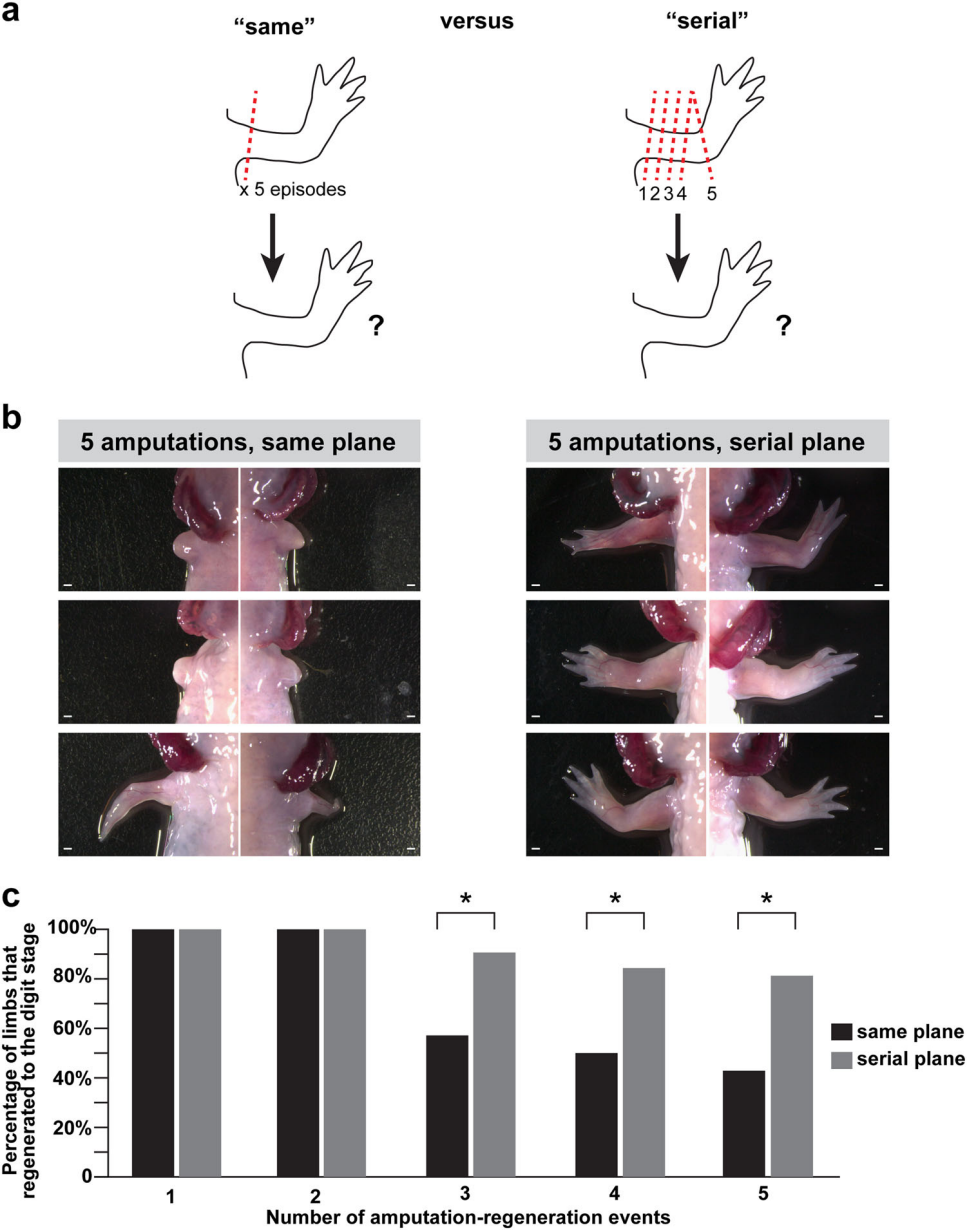
Regenerative decline after repeated amputation is less severe if the plane of amputation is shifted distally along the axis of the limb. Both forelimbs were amputated for each animal and allowed to fully regenerate. **a** Experimental overview. **b** Representative images of axolotl limbs after 5 rounds of amputation at the same plane (left panels) or with serially distal planes of amputation (right panels). **c** Quantification of the number of limbs able to regenerate digits after each amputation round following repeated amputation in the same plane or repeated, progressively distal amputations. Asterisk (*) indicates *p* < 0.05 (Fisher’s Exact Test). All scale bars equal to 1 mm

We speculated that our observed decline in regenerative capacity could potentially be due to the regenerative process itself being exhaustive such that numerous resources (e.g., progenitor cells) are being continuously drawn upon following each successive amputation that are not readily replenished. Alternatively, it is possible that repeatedly challenging the same population of cells within the limb could lead to the accrual of damaged tissue (e.g., fibrosis), which could interfere with successive rounds of regeneration. While these two possibilities are not mutually-exclusive, we decided to test if repeatedly amputating through the same anatomical plane leads to evidence of internal tissue scaring that might not happen if successive amputations occurred at different locations. We performed a repeated amputation study where we shifted the plane of amputation distally after each round of regeneration (Fig. 2a). By changing the plane of amputation distally we were able to challenge the tissues of the axolotl limb to undergo repeated rounds of regeneration, but each amputation involved the injury of tissue that had not been directly cut previously. For example, the fourth serially distal amputation would involve injuring tissue that is derived from three rounds of amputation (i.e., three regenerative cycles), but this newly regenerated tissue itself would be receiving an amputation (i.e., injury) for the first time.

After five amputation rounds, limbs challenged with serially-distal amputations exhibited notably better regenerative outcomes than limbs that were repeatedly amputated in the same region (Fig. 2b). Perturbations in the regenerated limbs following serial distal amputations were not as dramatic as siblings receiving repeat amputations at the same plane, and a significantly higher proportion of these limbs were able to regenerate to the digits stage after 3 or more rounds of amputation (Fig. 2c, *p* < 0.05, *n* = 32 serially amputated limbs vs. 14 limbs amputated at the same plane, Fisher’s exact test). Moreover, limbs that regenerated to the digits stage after serial distal amputations often exhibited better regenerative outcomes than those limbs amputated in the same plane (e.g., more digits; Supplementary Fig. 1c–d **)**. Collectively, these data indicate that problems with regenerative fidelity following repeated challenge may be due to localized events at the amputation plane.

### Limbs that fail to regenerate following repeated amputation exhibit signs of persistent fibrosis

**Fig. 3:**
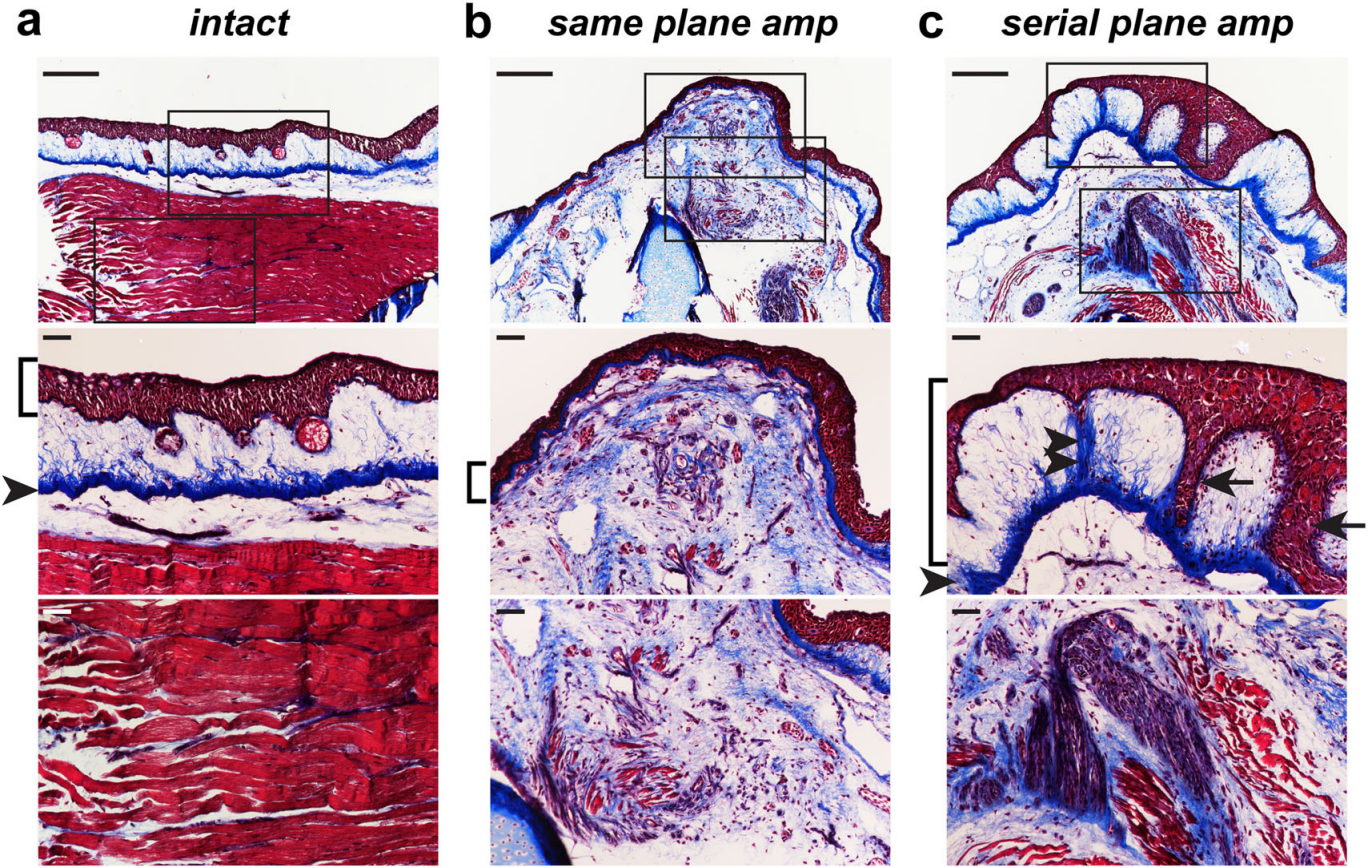
Aborted limb stumps exhibit persistent collagen deposition. A Masson’s trichrome stain was performed on limbs that failed to regenerate (“stumps”) following repeated amputation and on intact control limbs. **a** Column a (leftmost) depicts an intact specimen (no amputations). **b** Column b (middle) depicts a specimen following same-plane, repeated amputation. **c** Column c (right) depicts a specimen following serial-plane, repeated amputation. Top panels show representative images of intact control **a** and failed regenerates from limbs amputated in the same plane **b** and limbs amputated serially distally **c**. Middle and lower horizontal panels are higher magnification views of images in the top panels. Brackets delineate epidermis, arrowheads indicate substratum compactum (dermis), double arrowheads indicate connection between substratum compactum and epidermis, and arrows indicate epidermal tongues. Scale bars in top rows are 500 µm, and scale bars in middle and bottom rows are 100 µm

In other experimental systems (e.g., denervation, macrophage depletion, etc.), aborted limb regeneration is often accompanied by the deposition of fibrotic tissue.^8,9^ To determine whether loss of regenerative capacity following repeated amputation in the same plane results in fibrotic tissue deposition, we performed one last round of amputation and performed histological analyses on the stumps that failed to regenerate at any point during our study and on intact control limbs (Fig. 3a–c). Within several limbs that failed to outgrow following repeated amputation, we found the presence of extensive scar tissue as evidenced by collagen deposition proximal to the plane of amputation (Fig. 3b, *n* = 12 limbs examined). We also examined the few failed regenerates that resulted from serial distal amputations and found that they also exhibited extensive collagen deposition (Fig. 3c, *n* = 4 limbs examined). In addition to scar tissue, we also observed the presence of epidermal tongues that extended down to the substratum compactum in failed regenerates (Fig. 3c). To specifically consider possible aberrant collagen deposition in failed regenerates, we stained tissue sections with α-Collagen I and α-Collagen IV (Supplementaty Fig. 2). We found that both of these proteins appear dysregulated in the failed regenerates. For example, Collagen I is normally largely localized to the dermis, within the stratum compactum layer; however, in the failed regenerates, Collagen I is more diffuse in the dermis, and it is clearly visible in internal areas of the limb (Supplementary Fig. 2a–a’). Collagen IV is considerably more apparent in failed regenerates than in controls, specifically in the area between the muscle and epidermis (Supplementary Fig. 2b–b’). Taken together, these data suggest that limbs that failed to regenerate following repeated amputation implemented a scarring response in place of the full regenerative program.

### Transcriptional analyses of failed regenerates and identification of *amphiregulin* as a candidate antagonistic factor

**Fig. 4:**
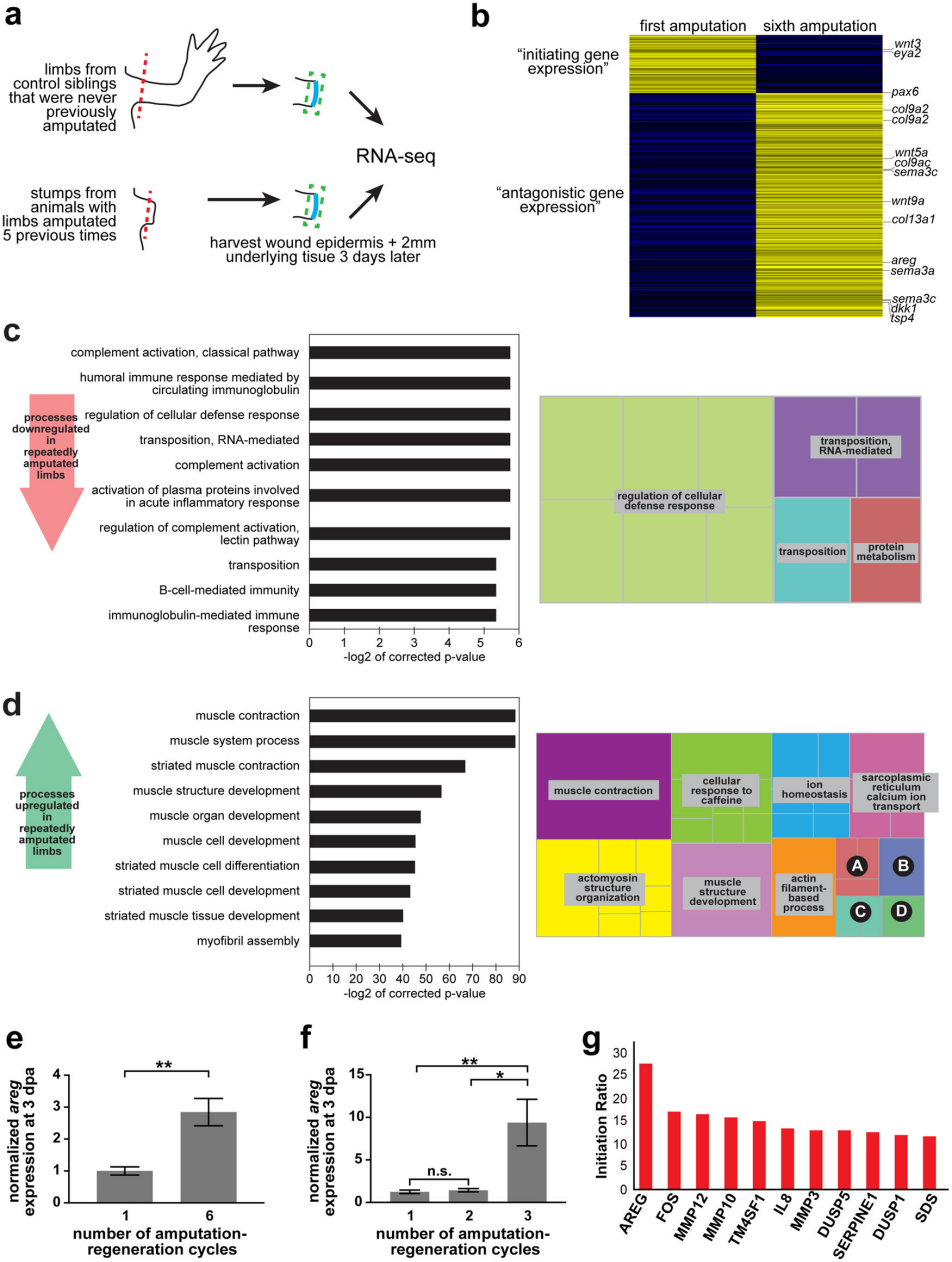
Transcriptomic analyses suggest an antagonistic expression pattern for *amphiregulin*. Failed regenerates that had undergone 5 amputations and sibling control limbs that had never been injured were amputated proximally and harvested at 3 days post-amputation. **a** Overview of RNA-sequencing strategy. **b** Heatmap showing the results of *k*-means clustering (*k* = 2) of significantly differentially expressed genes between repeatedly amputated limbs and control limbs. Several genes with known roles in regenerative processes are shown. **c** Gene Ontology analyses of genes that are downregulated in the repeated amputation condition. Panel on the left shows the top 10 most significantly enriched Biological Processes. Panel on the right is a treemap of all significantly enriched Biological Processes. **d** Gene Ontology analyses of genes that are upregulated in the repeated amputation condition. Panel on the left shows the top 10 most significantly enriched Biological Processes. Panel on the right is a treemap of all significantly enriched Biological Processes. “A”: forward locomotion; “B”: multicellular organismal process; “C”: directional locomotion; “D”: developmental process. A full list of significantly enriched Biological Processes can be found in Supplementary Table 2. **e** qRT-PCR showing normalized *areg* expression at 3 dpa in normally-regenerating control limbs (first amputation) and in limbs compromised by repeat amputation (sixth amputation). Error bars are SEM. *N* = 8 limbs per condition. Asterisks (**) denotes *p* < 0.01. **f** qRT-PCR showing normalized *areg* expression at 3 dpa in normally-regenerating control limbs (first amputation) and in limbs amputated twice and thrice. Error bars are SEM. *N* = 4-5 limbs per condition. Asterisks (**) denotes *p* < 0.01; Asterisk (*) denotes *p* < 0.05; n.s. denotes not significant. **g** Plot showing the 11 genes with the highest initiation ratio (ratio of early expression to late expression). Expression data for panel **g** were obtained from [32]

Motivated by our findings with repeated amputation, we asked whether we could use the failed regenerates in our study to uncover novel insights into mechanisms that may antagonize natural regeneration. Starting with the failed regenerates from animals that had already been through five amputation-regeneration events, we performed a single (sixth) amputation on these stumpy, failed regenerates. This sixth amputation plane was positioned slightly proximal to the previous amputation plane, essentially setting the amputation point within the region of demonstrated failed regeneration. At 3 days post-amputation, we performed RNA-sequencing analysis of the local tissue (i.e., wound healing stage, *n* = 4 failed regenerates) to identify factors that may be involved with regenerative decline (Fig. 4a). As a control, we also amputated and sequenced the limbs of sibling animals (that had not been previously injured) at 3 days post-amputation (*n* = 4 control limbs) (Fig. 4a). Using previously defined methods,^27^ we generated a de novo transcriptome and performed differential expression analysis. We uncovered 912 transcripts that were differentially expressed (FDR < 0.05) between normally-regenerating control limbs and limbs compromised by repeat amputation that are not engaged in the normal early regeneration response (Fig. 4b, each condition shown is the average of four biological replicates; genes are listed in Supplementary Table 1). Of note, we observed that far more transcripts were aberrantly upregulated (724 transcripts) than downregulated (188 transcripts) in the compromised limbs vs. the controls. We surmised that upregulation of a given transcript may reflect an expression pattern that is antagonistic to regeneration, while downregulation of a given transcript might reflect failure to adequately activate transcripts necessary to initiate regeneration (Fig. 4a). Given that nearly 80% of the transcripts in our dataset were upregulated in limbs that had been previously amputated five times, it is possible that the emergence of regenerative roadblocks could be the major driving force behind the regenerative decline of repeatedly challenged limbs.

Among the differentially expressed genes, we found several genes with known functional roles in regeneration in either other systems or in axolotl. Among genes downregulated in the repeatedly amputated limbs, we identified *eyes absent homolog 2* (*eya2*), upregulated during lens regeneration in young axolotls^28^ and required for eye regeneration in planaria.^29^ The genes upregulated in repeatedly amputated limbs included several types of collagen (*col2a1*, *col9a1*, *col9a2*, *col9a3*, and *col23a1*), *Dickkopf-related protein 1* (*dkk1*), *thrombospondin 4* (*thbs4*), and *semaphorin-3a* (*sema3a*). *Dkk1*, a Wnt-signaling inhibitor, and *thbs4* have separately been shown to antagonize axolotl limb regeneration,^30,31^ while *sema3aa*, has been shown to inhibit zebrafish heart regeneration.^32^ Intriguingly, we observed that both *connective tissue growth factor* (*ctgf*) and *amphiregulin* (*areg*), which have been both shown sufficient to cause fibrosis in mammalian contexts, were aberrantly upregulated in the limbs challenged by repeated amputation.^33–38^

We also performed Gene Ontology (GO) analyses on our RNA-seq data and found significant enrichment (FDR < 0.05) for GO terms such as “complement activation” and “regulation of cellular defense response” in the set of genes that are downregulated in repeatedly amputated limbs (Fig. 4c, Supplementary Table 2). Such enrichment may reflect a dampened immune response in repeatedly amputated limbs. GO terms such as “muscle contraction”, “actomyosin structure organization”, and “muscle structure development” were significantly enriched (FDR < 0.05) for genes that were upregulated in our dataset (Fig. 4d, Supplementary Table 2). It is possible that this may reflect an impaired ability of repeatedly amputated limbs to break down muscle during limb regeneration. Similarly, the “cellular response to caffeine” GO term was enriched in our upregulated set of transcripts, suggesting that related pathways could promote regenerative decline (Fig. 4d, Supplementary Table 2). Indeed, previous studies have shown that caffeine can interfere with wound healing, lending support to this hypothesis.^39^

Motivated by our ability to uncover known gene expression patterns that impair regeneration using failed regenerates, we asked whether we could use our dataset to identify a previously unknown gene expression relationship that could limit limb regeneration. We further sought to identify relationships that could antagonize limb regeneration during its initial stages (i.e., wound healing). To this end, we chose to focus our attention on the *amphiregulin* (*areg*) gene. We performed two separate qRT-PCR experiments (Fig. 4e, f). We first validated that *areg* expression is significantly higher at 3 dpa in limbs having undergone a sixth amputation vs. a first amputation (2.84-fold upregulated, *p* < 0.05, eight biological replicates per group, Fig. 4e). We separately derived a cohort of size/age-matched animals and compared *areg* expression at 3 dpa following one, two, or three amputation-regeneration cycles (Fig. 4f, 4-5 biological replicates per group). We found no significant difference in *areg* expression between one and two amputation-regeneration events, but a significant increase in *areg* expression between three and one events, as well as between three and two (*7.5-fold higher in third* vs*. first, p < 0.01, one-way ANOVA with Bonferroni post-hoc correction; 6.5-fold in third* vs. *second, p < 0.05, one-way ANOVA with Bonferroni post-hoc correction)*. Notably, the upregulation in *areg* expression upon the third amputation-regeneration event parallels the timeline for when limb loss is first observed in our original experiment (in a fraction of the population, between the second and third amputation-regeneration events). We also took advantage of a previously-published RNA-seq time course of axolotl limb regeneration,^40^ and we confirmed that in this independent study, *areg* expression was enriched early in limb regeneration. This expression was markedly higher than that of any other gene at that time in this pre-existing dataset (Fig. 4g, Supplementary Fig. 3). Together, these data show that *areg* expression is higher in limbs that have undergone repeated amputation and they suggest that aberrantly high *areg* expression could disrupt axolotl limb regeneration by antagonizing its progression past the earliest stages of the regenerative program.

### The expression of *amphiregulin* is restricted to the early wound epithelium during axolotl limb regeneration

Amphiregulin is a member of the epidermal growth factor (EGF) family that has been shown to play a role in a wide range of biological processes, including liver and intestinal epithelium regeneration, psoriasis, and liver fibrosis.^36,41–44^ In mammals, *amphiregulin* is expressed at low levels in the epidermis, rapidly induced following injury to the skin, and upregulated in pathological skin conditions such as psoriasis.^44,45^ Moreover, transgenic expression of human *areg* in the basal or suprabasal epidermal regions of mice results in hyperproliferation of the epidermis and a psoriatic-like skin condition.^46^ Collectively, these data suggest that *areg* plays a functional role in integumentary homeostasis.

**Fig. 5:**
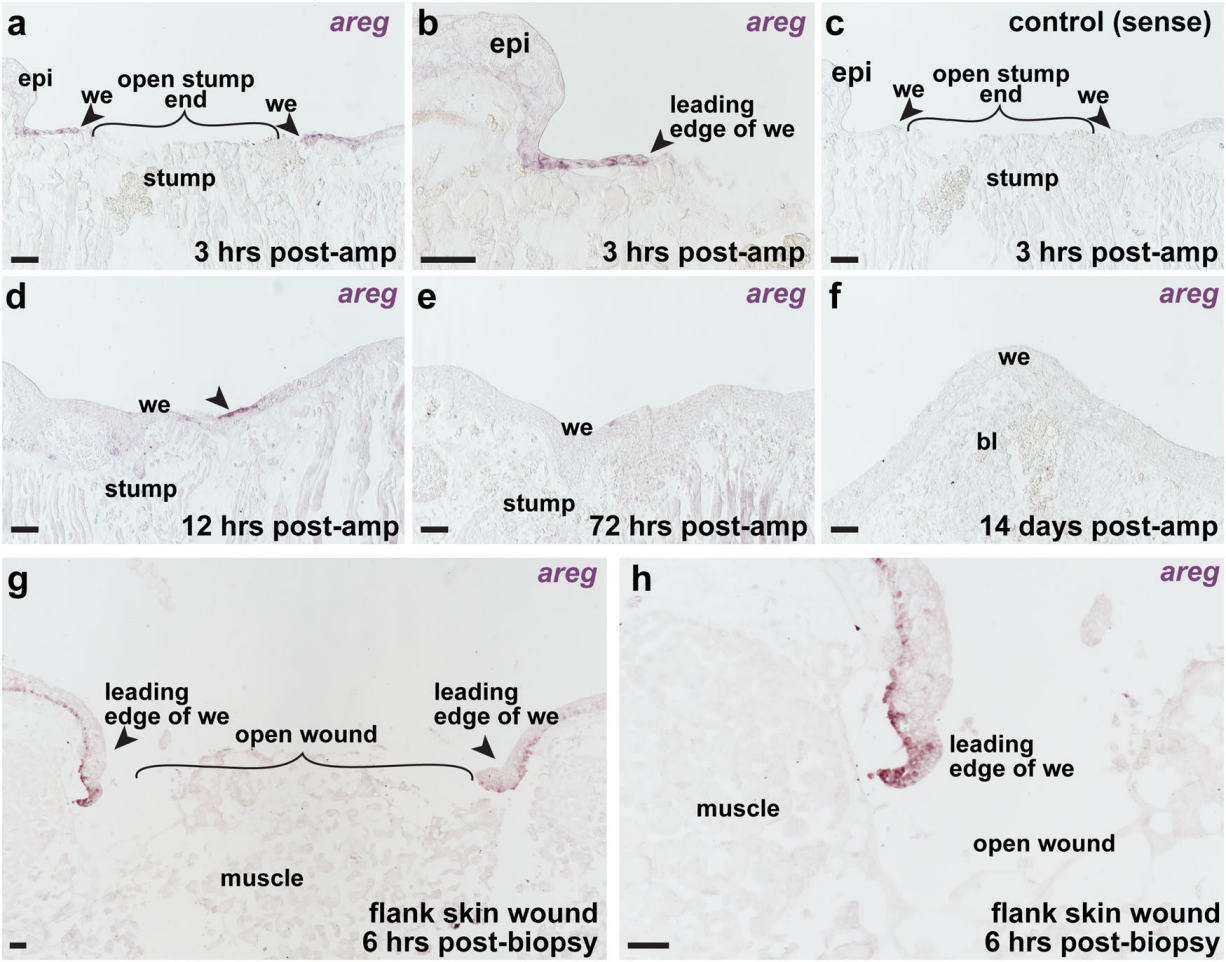
*Amphiregulin* is expressed during the wound healing stage of limb regeneration. In situ hybridization analyses of *areg* expression in wild-type juvenile axolotls; **a**–**f** are regenerating limb samples; **g**–**h** are flank skin wound samples. **a** Expression of *areg* at 3 h post-amputation. Arrowheads indicate expression of *areg* in the leading edge of the wound epidermis. *Epi* epidermis. **b** Higher magnification view of panel a. **c** Section stained with sense control probe at 3 h post-amputation. **d** Expression of *amphiregulin* at 12 h post-amputation. Arrowheads indicate expression of *areg* in the wound epidermis (we). **e** Tissue section stained with *areg* anti-sense in situ probe at 72 h post-amputation. **f** Tissue section stained with *areg* anti-sense in situ probe at 14 days post-amputation (medium bud blastema). **g** In situ hybridization showing *areg* expression in leading edge of wound epidermis at 6 h post-biopsy. **h** Higher magnification view of panel **g**. *We* wound epidermis, *bl* blastema. Images are representative of four biological replicates per time point. Scale bars are 100 µm

Analysis of *areg* expression by in situ hybridization revealed that it is rapidly induced in the wound epithelium within the first few hours of limb regeneration (Fig. 5a, b). Sections treated with control (sense) *areg* in situ probe did not show any staining in the wound epithelium (Fig. 5c). While *areg* expression could still be observed at 12 h post-amputation (Fig. 5d), its expression becomes virtually undetectable by 3 days post-amputation and was not observed in the wound epidermis of medium bud stage blastema (Fig. 5e, f). Notably, *amphiregulin* expression appeared to be enriched in the leading edge of the wound epidermis at 3 h post-amputation (Fig. 5a, b), suggesting that it may play a role in wound closure following amputation. Interestingly, *amphiregulin*’s rapid induction in axolotl wound epidermis is reminiscent of its quick induction in injured mammalian skin, suggesting that it may play a conserved role in epithelial biology. To address whether *areg* expression in the epidermis is specific to wound epidermis forming across amputated limbs or if it might be a more general hallmark of skin wound healing, we performed skin punch biopsies. By RNA in situ hybridization, we observed robust staining for *areg* transcript in epidermis, at the edges of the healing skin wound (Fig. 5g, h).

We also assessed *amphiregulin*’s expression in the stumpy limbs of repeatedly amputated limbs (several months after regenerative failure) and found that several stump specimens expressed *areg* in the epidermis whereas *areg* expression appeared to be absent in the epidermis of intact control limbs (Supplementary Fig. 4a, d). Furthermore, we amputated limbs that failed to regenerate after prior injury and examined *areg* expression at 12 h post-amputation and 3 days post-amputation via in situ hybridization and found that *areg* appeared to be expressed by similar cell populations in amputated stump and control limbs (Supplementary Fig. 4c, f). Thus, within the limits of in situ hybridization, we conclude that *areg* is not ectopically expressed by a different population of cells following amputation of failed regenerates; rather, its upregulation may reflect an increase in production by epidermal cells.

### Persistently high *amphiregulin* expression impairs axolotl limb regeneration

**Fig. 6:**
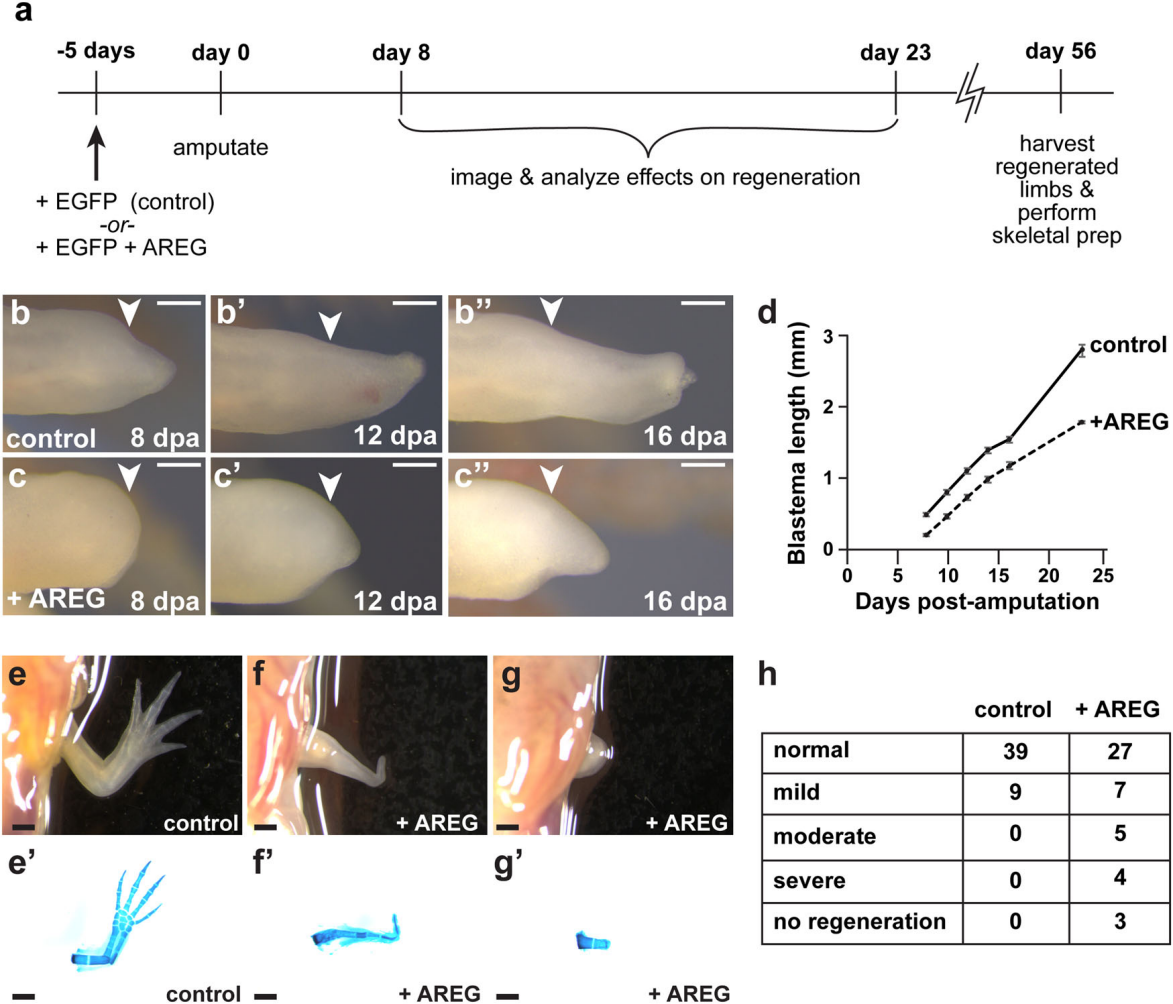
Overexpression of *amphiregulin* disrupts limb regeneration. Axolotl limbs with no prior injuries were electroporated with either plasmid encoding GFP (control) or plasmids encoding GFP plus AREG. **a** Overview of experimental strategy. **b-b**”) Representative images of EGFP control limbs from 8 to 16 days post-amputation (dpa). Arrowheads denote amputation planes. **c-c**”) Representative images of *areg* mis-expressing limbs from 8 to 16 days post-amputation (dpa). Arrowheads denote amputation planes. **d** Quantification of blastema lengths in **b**–**b**” and **c**–**c**”. *N* = 24 animals for control and *N* = 23 animals for *areg* overexpression. **e-g** Representative images of control limbs and limbs exhibiting severe regenerative defects or no regeneration beyond the stylopodium following *areg* overexpression. **e**’–**g**’) Representative skeletal preparations of control limbs and limbs exhibiting severe regenerative defects or no regeneration beyond the stylopodium following *areg* overexpression. **h** Quantification of defects after control *egfp* and *areg* overexpression. The two groups exhibit significantly different morphologies (*p* < 0.01, Fisher’s exact test). *N* = 48 limbs for control and *N* = 46 limbs for *areg* overexpression

Our earlier transcriptional analyses suggested that persistent expression of *areg* could antagonize limb regeneration and disrupt its ability to progress to later stages in the regenerative cycle. To test this hypothesis, we overexpressed *amphiregulin* in the intact limbs of juvenile axolotls via the electroporation of a plasmid encoding the axolotl AREG protein driven by a constitutive promoter (pCAG-*areg*); to monitor electroporation efficiency, we co-delivered a similar plasmid encoding enhanced GFP (pCAG-*egfp*) (Fig. 6a, Supplementary Fig. 5). Control limbs were electroporated with only pCAG-*egfp* (Fig. 6a, Supplementary Fig. 5). We confirmed the expression of AREG protein specifically in limbs electroporated with pCAG-*areg* using antibodies against AREG (Supplementary Fig. 5). After allowing the animals to recover for 5 days, we amputated the limbs and monitored blastema formation and development, as well as overall regenerative fidelity (Fig. 6a).

We observed that overexpression of *amphiregulin* in axolotl limbs delayed regeneration and progression to the blastema stage (Fig. 6b, c”). At 8 dpa, 100% of control limbs showed clear signs of blastema formation (*n* = 48 limbs). However, only 67% of *areg*-overexpressing limbs (*n* = 31/46 limbs), a significantly smaller fraction (*p *< 0.001, Fisher’s exact test), were able to form a blastema by 8 dpa. Furthermore, we quantified blastema lengths from 8 dpa to 23 dpa and found that *areg*-overexpressing limbs had shorter blastema (>2-fold smaller at 8 dpa) at every time point relative to controls (*p* < 0.001; *n* = 24 control animals, *n* = 23 animals with *areg*-overexpressing limbs) (Fig. 6d).

In addition to delayed regeneration, several limbs that overexpressed *areg* exhibited abnormal regenerative outcomes ranging from complete or near-complete regenerative failure to the loss of several major skeletal elements at the end of the regenerative cycle (~8 weeks post-amputation) (Fig. 6e–g’). Next, we quantified the number of regenerated limbs from each group that exhibited normal skeletal morphology, mild skeletal defects (e.g., digit truncation, carpal fusions, digit outgrowths, etc.), or severe defects (e.g., complete failure to regenerate, loss of major skeletal elements such as radius/ulna and whole digits, etc.) (Supplementary Table 3). We found that *areg* overexpression led to significantly poorer regenerative outcomes relative to controls (Fig. 6h, *p* < 0.01). Thus, our data indicate that *areg* dysregulation antagonizes the ability of axolotl limbs to progress past the early stages of limb regeneration.

### *Amphiregulin* mis-expression promotes abnormal epithelial thickening and impairs internal proliferation during limb regeneration

**Fig. 7:**
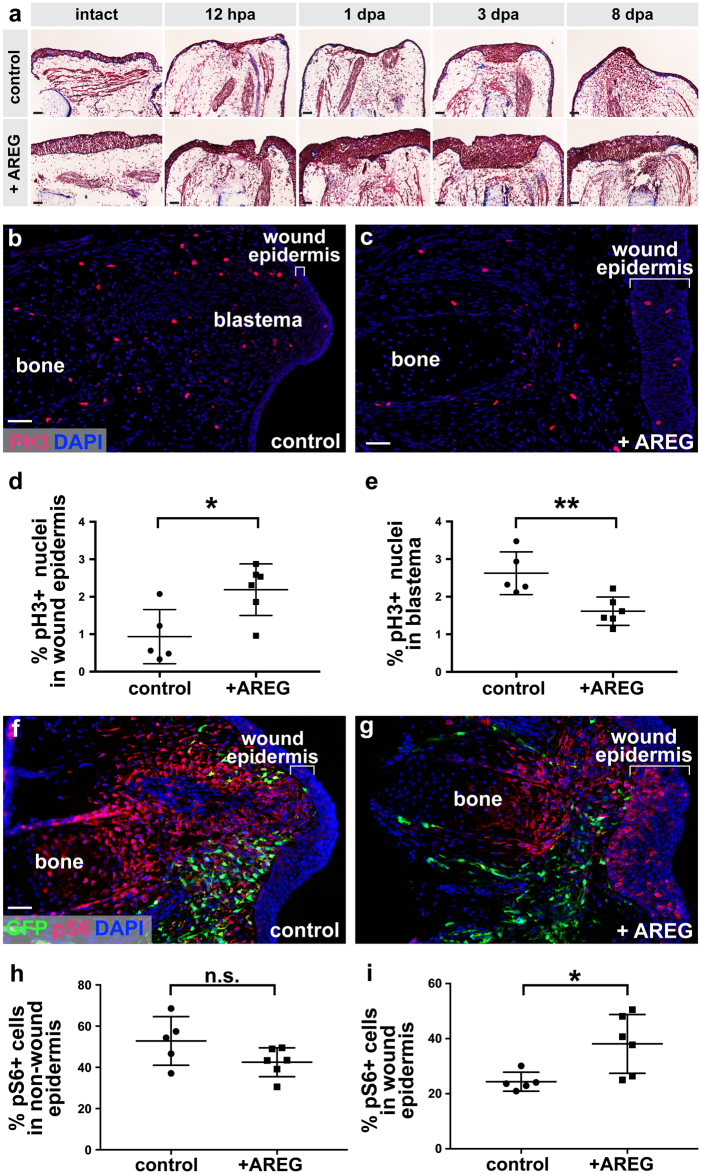
Overexpression of *amphiregulin* results in abnormally thick wound epidermis, alterations in cellular proliferation, and increased mTOR signaling in the wound epidermis during limb regeneration. Axolotl limbs with no prior injuries were electroporated with either plasmid encoding GFP or plasmids encoding GFP and AREG. **a** Multi-timepoint Masson’s trichrome staining of tissue sections from *egfp* control or *areg* overexpressing limbs. For 0 dpa, 12 hpa, 1 dpa, and 3 dpa, *N* = 5 limbs per group per timepoint. For 8 dpa, *N* = 5 limbs for control and *N* = 6 limbs for *areg* overexpression. **b** Representative immunofluorescent staining of phospho-Histone H3 (pH3) on tissue sections from *egfp* control limbs at 8 dpa. **c** Representative immunofluorescent staining of phospho-Histone H3 (pH3) on tissue sections from *areg* overexpressing limbs at 8 dpa. **d** Quantification of the percentage of pH3-positive nuclei in the wound epidermis. Asterisk (*) indicates *p* < 0.05. *N* = 5 limbs for control, and *N* = 6 limbs for *areg* overexpression. **e** Quantification of the percentage of pH3-positive nuclei in non-wound epidermal tissues. **f** Representative immunofluorescent staining of phospho-rpS6 (pS6) on tissue sections from *egfp* control limbs at 8 dpa. **g** Representative immunofluorescent staining of pS6 on tissue sections from *areg* overexpressing limbs at 8 dpa. **h** Quantification of the percentage of pS6 positive cells in non-wound epidermal tissues. *n.s.* not significant. *N* = 5 limbs for control, and *N* = 6 limbs for *areg* overexpression. **i** Quantification of the percentage of pS6 positive cells in the wound epidermis. Asterisk (*) indicates *p* < 0.05. *N* = 5 limbs for control, and *N* = 6 limbs for *areg* overexpression. Scale bars are 100 µm

After 5 days post-electroporation, we noticed that the stylopodium of limbs that overexpressed *areg* were considerably larger than their control counterparts (Supplementary Fig. 5). We quantified the stylopodial width of electroporated limbs and found that they were significantly thicker in limbs that overexpressed *areg*. This finding held true even when normalized to their non-treated hindlimb stylopodium (Supplementary Fig. 5). When investigated further, histological examination of intact limbs that overexpress *amphiregulin* revealed that they had significantly thicker epidermis than control limbs (Fig. 7a *p* < 0.05, *n* = 5 limbs per condition, Supplementary Fig. 6a, b), which is consistent with previous studies showing that *areg* promotes epidermal growth in mammals.^44,46–49^ Furthermore, the observed enlargement of limbs that misexpress *areg* appeared to be primarily due to epidermal thickening (Supplementary Fig. 6a–d). The internal portion of *areg*-misexpressing limbs was not significantly thicker than their control counterparts while their total epithelial thickness (sum of both epithelial layers in longitudinal section) was more than 3 times that of control limbs (Supplementary Fig. 6c, d).

Upon amputation, limbs that misexpressed *areg* formed a wound epithelium that was thicker than that of their control counterparts at several stages of regeneration, including the blastema stage (~8 dpa, Fig. 7a, *p* < 0.001). These data suggest that formation of an abnormal wound epithelium could be one contributing factor to the regenerative defects that we observed in limbs that overexpress *areg*. We considered whether the thickened wound epidermis in *areg*-overexpressing limbs might not be molecularly similar to normal, functional wound epidermis. To test this possibility, we stained *areg*-overexpressing limbs at 8 dpa with the most widely-used wound epidermis marker in salamanders, WE3.^50^ We found that wound epidermis in *areg*-overexpressing limbs is still reactive to this antibody (Supplmentary Fig. 7), indicating that by this measure, the nature of the wound epidermis may be similar to normal wound epidermis despite being thickened.

We observed that the wound epidermis of *amphiregulin* mis-expressing limbs had significantly more cells than the wound epidermis of control limbs (~1.6 fold more nuclei at 8 dpa, *p* < 0.01, Fig. 7b, c, f, g). These data suggested that increased proliferation of wound epidermal cells may be a major factor in the epidermal thickening that we observed. To test this, we performed proliferative studies on limbs that mis-expressed *areg* using the mitosis marker phospho-Histone H3 (ser10) (pH3). We observed an increase in the percentage of pH3 + nuclei in the wound epidermis of limbs treated with *areg* (Fig. 7b–d, *p* < 0.05), which is consistent with our observation that these limbs have thickened wound epidermis and more wound epidermal cells. In contrast, we found that the non-wound epidermal tissues of *areg*-treated limbs had a significantly lower percentage of pH3 + nuclei relative to controls (Fig. 7b, c, e, *p* < 0.01). Interestingly, we did not detect a significant difference in the percentage of pH3 + nuclei in internal and epidermal tissues in intact limbs that misexpress *areg* (Supplementary Fig. 6e, f), suggesting that *areg* misexpression may have distinct biological effects in regenerative and homeostatic contexts. Together, these data suggest that the formation of an abnormal wound epidermis and diminished proliferative capacity of non-wound epidermal tissues may contribute to the antagonistic effects of *areg* mis-expression on limb regeneration.

### *Amphiregulin* mis-expression increases mTOR signaling in the wound epidermis

We next aimed to identify potential downstream effectors that could be mediating the antagonistic effects of *areg* on limb regeneration. Mammalian Amphiregulin is known to bind the EGF Receptor (EGFR).^51^ A recent study in axolotls has shown that pharmacological inhibition of EGFR can lead to soft tissue regression at the amputation site; this effect is accompanied by a decrease in proliferative epidermal cells compared to controls.^52^ Thus, some aspects of blocking EGFR in limb regeneration are opposite of what we observe when we misexpress a putative activating ligand. It is therefore possible that EGFR may transduce the effect of Amphiregulin misexpression. However, many mediator pathways operate downstream of EGFR activation to elicit a variety of biological effects. We sought to investigate what intracellular mediator system might transduce the Amphiregulin signal in our experiment. Previous studies have shown that EGFR signaling can activate the mTOR pathway, and hyperactivation of PI3K/mTOR signaling has been shown to promote accelerated epithelial wound healing.^53,54^ Given the distinct effects of *amphiregulin* on the wound epidermis in limb regeneration, we focused our attention on the mTOR signaling pathway.

Phosphorylated ribosomal protein S6 (pS6) is a well-studied readout of active mTOR signaling.^55^ To determine whether *areg* mis-expression leads to perturbed mTOR signaling during limb regeneration, we examined the presence of pS6 in regenerating limbs. We found that mis-expression of *amphiregulin* did not result in a significant change in the percentage of non-wound epidermal cells that had active mTOR signaling (pS6 positive at 8 dpa) (Fig. 7f–h). In contrast, we observed a significant increase in mTOR signaling in the wound epidermis (Fig. 7i), suggesting that mTOR signaling may mediate the downstream effects of *amphiregulin* overexpression on the wound epidermis during limb regeneration.

## Discussion

The lack of regenerative capabilities in adult mammals underscores the importance of understanding how highly-regenerative organisms such as axolotls replace lost appendages. One effective approach to tackling this problem is by pushing regenerative programs in organisms such as axolotls to a non-regenerative state and identifying factors that may be related/altered in nonregenerative species. Gaining a greater understanding of the regenerative limitations of axolotls may provide insights critical for uncovering regenerative limitations in mammals.

Here, we probed the limits of axolotl limb regeneration by challenging them with repeated amputation. We observed both a decline in regenerative fidelity as well as ability to regenerate beyond the plane of amputation (Fig. 1). These findings suggest that there is indeed a limit to the ability of axolotl limbs to continue to regenerate with near perfection. Furthermore, our studies with repeated, serially distal amputations suggest that the decline may be due in part to recurrently injuring the same tissue instead of globally exhausting the regenerative cycle (Fig. 2). One longstanding hypothesis in the field of regenerative biology is that there is a “tug of war” between the scarring process and regenerative process, and humans may possess limited capabilities because the molecular programs that drive the former process win this competition following injury.^56,57^ In support of this, we found evidence of extensive collagen deposition in limbs that failed to regenerate after repeated amputation (Fig. 3, Supplementary Fig. 2). It is possible that the accumulation of fibrotic tissue could play a role in the failure of axolotl limbs to regenerate when subjected to repeated amputation, but future studies are needed to fully test this hypothesis. Future studies examining the consequences of repeated amputation in the completely adult context, starting when the animals are fully mature (~1 year old), may provide fruitful avenues for discovering additional limitations to the regeneration program. Another question worth exploring is whether any possible “recharging” effect following very lengthy periods between regeneration and re-amputation might allow for the program to recover. This will be an important future experiment that could reveal different outcomes from our existing findings. Our existing findings have proven fruitful for discovering molecular constraints on limb regeneration. However, if axolotls can engage in perfect repeated regeneration if they are given extended recovery times, contrasting that finding with our data could be important for understanding limitations. The opposite experiment, examining the consequences of shorter amputation-regeneration cycles, may also provide important clues for understanding what cellular and/or molecular factors may underlie success or failure. Future experiments may also focus more pointedly on individual tissue types—nerve, muscle, skin, et cetera—to determine how each might be impacted with successive amputations, and how specific tissues might contribute to regenerative outcomes.

We surmised that the failed regenerates generated in this study could provide valuable clues about roadblocks that could arise to limb regeneration. In line with this, our transcriptomic analyses of failed regenerates at an early stage in regeneration uncovered gene expression patterns that are known to disrupt regeneration (Fig. 4). Our data also suggested that dysregulation of the EGF family member *amphiregulin* could be antagonistic to limb regeneration, as increasing the expression of this gene during limb regeneration severely disrupted this process (Figs. 6, 7). Previous studies have implicated a role for *amphiregulin* in psoriasis, an autoimmune disease characterized by hyperproliferative epidermis.^47–49,58^ Our work with *amphiregulin* in axolotl limbs is in line with these findings as it is sufficient to promote epidermal thickening in intact limbs (Fig. 7a and Supplementary Fig. 6a–d) and promotes increased cell proliferation in wound epidermis (Fig. 7b–e). Intriguingly, while we detected a significant decrease in proliferation of internal tissues in regenerating limbs that misexpress *areg* (Fig. 7), we did not observe a decrease in proliferation of internal tissues in the intact state (Supplementary Figure 6a, b, e). These data suggest that *amphiregulin* could have different functions in homeostasis and regeneration. Future investigation of *amphiregulin*’s differential effects in homeostatic and regenerative contexts will yield valuable insight into the role that this factor plays in limb regeneration.

Given the linkage between *amphiregulin* and psoriasis, it is tempting to speculate that dysregulation of *amphiregulin* expression could promote a hyperactive wounding response. Previous research suggests that humans with psoriasis have an increased healing rate; in particular, this study found that individuals with psoriasis healed wounds created by skin biopsy more rapidly than individuals with normal skin.^59^ Our observations that *amphiregulin* mis-expression leads to increased mTOR signaling in the wound epidermis are also consistent with this hypothesis, as higher levels of PI3K/mTOR signaling promote a swifter healing response in mammals (Fig. 7f–h).^53^ Our data also suggest the possibility that the wound healing stage serves as a regenerative checkpoint following limb amputation and that resolution of this phase may be necessary to progress to later stages of regeneration. If this is the case, then failure to downregulate wound healing signals would be expected to slow or halt regeneration altogether. Thus, if Amphiregulin serves as a wound-healing signal during limb regeneration, then the prolonged presence of this molecule could potentially interfere with the progression to later regenerative stages, such as blastema formation. Although this is an exciting possibility with important implications for mammalian regeneration, further work is needed to test such a model.

Several studies involving skin suturing and intracoelomic insertion of limbs immediately after amputation have demonstrated that failure to form a wound epidermis is inhibitory to limb regeneration.^10–12^ Our studies with *amphiregulin* hint at the possibility that driving wound epidermis in the opposite direction (i.e., having too much wound epidermis) can also impede regeneration. Interestingly, the thickened skin in our study is reminiscent of previous research showing that frog limbs that fail to regenerate are covered by a thickened layer of skin.^60^ Future studies involving the transplantation of thickened skin to naïve limbs may shed additional insight on the possibility that thickened wound epidermis can impede limb regeneration. Future studies will also be important for addressing the molecular nature of wound epidermis in repeatedly-amputated limbs and in limbs overexpressing *areg* beyond the single WE3 marker we report here. Dysregulation of critical wound epidermis molecules remains an important possibility worth considering in either of these contexts.

Roles for *amphiregulin* in both fibrosis and regeneration in mammalian liver have been discovered. Mice with loss-of-function mutations in *areg* show decreased cellular proliferation following partial hepatectomy.^43^ In samples from both cirrhotic human livers and a rat model of liver cirrhosis, *amphiregulin* levels are elevated.^43^ Together, these data suggest that a balance between the need for *amphiregulin* in early healing and the pathological consequences of too much *amphiregulin* may exist across many species and organs. AREG has also been shown to mediate the fibrotic effects of TGF-β1 expression in mouse lungs.^36^ In the time frames examined to date, we have not yet observed mis-expression of *areg* to be sufficient to cause hallmarks of fibrosis in limbs; future work, perhaps aimed at extending the temporal window of *areg* expression, will be required to examine this possibility.

Recently, a report has found that newts are capable of efficiently clearing senescent cells after multiple rounds of limb amputation.^61^ Axolotls were examined after a single amputation and found to also successfully clear senescent cells through macrophage engulfment.^61^ While this study did not report on the patterning outcomes following full regeneration-amputation cycles, as we present here, it does provide a useful starting point for considering mechanisms at play following repeated injury. For instance, a simple model with accumulation of senescent cells over repeated deployment is not likely to be responsible for the severe regenerative outcomes following repeated regeneration-amputation cycles.

Our findings presented here indicate that even the highly-regenerative axolotl has limitations to its regenerative capabilities. We also show that these limits can be leveraged to discover factors that present obstacles to the regenerative process. While providing the right regenerative stimuli may be critical for promoting regeneration in humans, it may be just as important to remove or reduce antagonistic signals to improve the outcomes of regenerative therapies. The analyses and data that we provide will serve as a valuable foundation for identifying and studying processes that impede mammalian regeneration. Further exploration of the insights and gene expression relationships that we uncovered in this study have high potential for advancing our understanding of the regenerative roadblocks that mammals face after injury.

## Methods

### Animal experimentation

All animal experimentation was performed in accordance with Harvard Medical School’s Institutional Animal Care and Use Committee regulations and approved under animal experimentation protocol #04160. Leucistic axolotls were used for all animal experimentation and maintained as previously described.^27^ Due to the cannibalistic nature of axolotls, we began our study by separating a cohort of axolotls prior to hatching and placing them into individual containers. Because prior research has shown that bite injury can lead to poor regenerative outcomes, this early separation was crucial for avoiding confounding variables.^62^ The individualized housing also had the added benefit of allowing us to monitor the regenerative outcomes of each animal after successive rounds of amputation. Starting sample sizes were based on prior studies in our laboratory with survival requiring up to 1 year. Only animals with perfectly-formed limbs were included in the study. Animals were randomly assigned to experimental groups. Blinding was not performed when assigning animal groups, but blinding was performed for subsequent data analyses when possible.

For amputations and electroporations, axolotls were first anesthetized in 0.1% tricaine. After completion of the surgical procedure, axolotls were allowed to recover overnight in 0.5% sulfamerazine. For repeated amputations in the same plane, both forelimbs of axolotls were amputated at the mid-point between the girdle and mid-stylopodium (diagrammed in Fig. 1a). Following amputation, the bone was trimmed back to allow for efficient wound epidermis formation. Amputations were conducted in a similar manner for both forelimbs of animals undergoing repeated, serially distal amputations (amputation planes diagrammed in Fig. 2a). The first amputation (both same plane and serially distal planes) were performed on axolotls at approximately 2 months post-hatching (~3–4 cm in length). Limbs were allowed to fully regenerate before the next round of amputation (on average, every 13 weeks).

### Microscopy

A Leica M165 FC stereomicroscope was used to capture all whole mount images e.g., skeletal preparations). Skeletal preparations in Fig. 1b and Fig. 6e’–g’ were imaged in a 1:1 glycerol to 1% potassium hydroxide solution. For imaging of blastema in Fig. 6b, c”, animals were anesthetized in 0.1% tricaine and imaged ventrally. Tissue sections were imaged with a Nikon Eclipse Ni microscope using NIS-Elements software. ImageJ^63^ and Leica Application Suite software were used to make specimen measurements.

### Library preparation and RNA-sequencing

Limbs that failed to regenerate were amputated 1 mm proximal to the previous plane of amputation, and control limbs were amputated at the same anatomical location (Fig. 4a). At 3 dpa, the tissue 2 mm proximal to the amputation was harvested, and total RNA was purified from the tissue using the TRIzol reagent (Life Technologies, #15596018). Four biological specimens, from separate animals, were used to generate RNA and were separately processed as biological replicates for each of the two conditions. For each sample, 1 µg of total RNA was processed via the Illumina TruSeq v2 protocol to generate barcoded sequencing libraries. Paired-end, 50-bp sequencing was performed on an Illumina HiSeq 2500 sequencer at Harvard Medical School’s Biopolymers facility.

### RNA-sequencing analyses

Transcriptome assembly, annotation, and differential gene expression analyses were performed by Stirplate (http://stirplate.io/). Genes and transcripts were assembled from the RNA-sequencing data with Trinity^64,65^ and annotated with Trinotate (blastp and blastx were run against the SwissProt datablase, and Hmmer was run against the Pfam database)^66–68^ (http://hmmer.org/). RSEM software^69^ was used to quantify raw counts of RNA-Seq fragments mapping to transcripts for each sample, and an FPKM (Fragments Per Kilobase of transcript per Million mapped reads) threshold of two was applied to the initial assembly in order to generate the final transcriptome. The counts produced by RSEM were analyzed with edgeR^70^ to identify differentially expressed transcripts. A false discovery rate threshold of 0.05 was used to determine significance.

A pseudocount of “1” was applied to all TMM-normalized FPKMs of significantly differentially expressed transcripts. Transcripts were then log-transformed and median centered in Cluster 3.0.^71^ Cluster 3.0 was then used to perform k-means clustering on both genes (i.e., transcripts, *k* = 2) and arrays (i.e., samples, *k* = 2), using 1000 iterations and the Spearman Rank Correlation similarity metric for both. Clustering results were visualized using Java Treeview.^72^

The significantly upregulated and downregulated gene lists were tested for enrichment in relevant GO categories using BiNGO.^73^ Custom GO annotation reference libraries for “Biological Process”, “Cellular Component”, and “Molecular Function” were built using the GO terms provided by Trinotate. Enrichment in each reference ontology library was tested via a corrected hypergeometric test (Benjamini & Hochberg FDR correction) at the *p* = 0.05 level. Significantly enriched “Biological Process” GO terms were visualized using REViGO,^74^ with obsolete GO terms being removed prior to visualization.

For the analyses in Fig. 4g, data were obtained from a previously published transcriptomic study on axolotl limb regeneration (https://axolomics.org/sites/default/files/Stewart_Gene_Expression_Across_TimecourseTPMs_0.txt).^40^ A pseudocount of “1” was added to all TPMs (Transcripts per Kilobase Million) for each time point for all genes. Transformed TPMs from 3 h post-amputation to 3 days post-amputation were then averaged and then divided by average gene expression across all other time points (i.e., 0 dpa and 5–28 dpa) to generate a ratio of each gene’s early expression values to all other time points.

### Histology and immunohistochemistry

Tissues were fixed in 4% paraformaldehyde, taken through a series of sucrose solutions for cryopreservation (beginning with 5% sucrose in PBS and ending with 30% sucrose in PBS), and embedded in optimal cutting temperature compound (O.C.T). Samples were sectioned at a thickness of 16 µm and stored at −80 °C. For Masson’s Trichrome staining, samples were brought to room temperature, rehydrated in PBS, and then fixed for 10 min in 4% paraformaldehyde prepared in PBS. Following fixation, slides were rinsed in PBS and distilled water and then stained with Polysciences, Inc’s Masson’s Trichrome Stain kit (#25088-1).

For immunohistochemistry, slides were brought to room temperature, rehydrated in PBS, and then incubated with blocking solution (1X PBS with 0.1% Triton X-100 and 2% Bovine Serum Albumin) for 1 h at room temperature. Slides were incubated with rabbit anti-phospho Histone H3 (Ser10) (1:400, Millipore #06-570) overnight at 4 °C. Samples were washed and then incubated with Cy3-conjugated goat anti-rabbit IgG (H + L) (1:100, Jackson Immunoresearch # 111-165-003) for 1 h at room temperature. DAPI (1.4 µmol/L) in PBS was applied to slides for 10 min, and slides were mounted with Hydromount (National Diagnostics #HS-106). For detecting Collagen I, we used SC-59772 (Santa Cruz Biotech), 1:100. For detecting Collagen IV, we used ab6586 (Abcam), 1:100. For detecting AREG expression in fixed tissue following plasmid mis-expression, we used MAB262 (R&D Systems), 1:100. For wound epidemis, we used WE3 (DSHB), 1:10.

For *areg* overexpression studies, harvested tissues were sectioned through completely (Fig. 7a, cryosections, 16 μm). Sections from the middle of the specimens, as indicated by presence of bone, were used for further analyses (i.e., epidermal and wound epidermal thickening studies, proliferative studies, etc).

For mTOR signaling analyses, sections from regenerating limbs at 8 dpa (medium-bud-stage blastemas) were fixed with 4% paraformaldehyde for 20 min. Following post-fix, sections were rinsed with PBS and permeabilized with 0.5% Triton-X100 for 20 min. Following permeabilization, sections were rinsed in PBS and boiled in 0.1 M sodium citrate prior to blocking (2% BSA for 30 min at room temperature). Sections were incubated with rabbit anti-phospho-ribosomal protein S6 (Ser235/236, 1:200; Cell Signaling) followed by incubation with Cy3-conjugated secondary antibodies. Sections were then stained with DAPI (Roche) for 5 min before mounting. The percentage of cells exhibiting positive pS6 staining (defined as nuclei encompassed by pS6 staining in Fig. 7f–i) wound epidermis and internal tissues was quantified by a blinded observer.

### qRT-PCR

Tissue was extracted at 3 dpa and placed in TRIzol Reagent where it was homogenized using sterile pestles. RNA was extracted following the recommended TRIzol protocol or a combined TRIzol/RNeasy MinElute cleanup (Qiagen). One microgram RNA was used as input to generate cDNA using High-Capacity cDNA Reverse Transcription Kit (Thermo Fisher Scientific). cDNA was diluted 1:20. qPCR was performed using iTaq Universal SYBR Green Supermix (Bio-Rad) in 20 μl total volume with 1 μl cDNA input following the recommended protocol in a CFX384 C1000 Touch Real-Time machine (Bio-Rad). qPCR settings for *ef1α*: 95 °C 30 s, and 40 cycles of 95 °C 10 s, 60 °C 40 s. qPCR settings for *areg*: 95 °C 30 s, and 40 cycles of 95 °C 10 s, 55.4 °C 10 s, 72 °C 20 s. Specificity was accessed using a melt curve analysis. Samples were run in technical triplicates; each group had 4–8 biological replicates. Expression was calculated by comparing *C*_t_ values to a standard curve. Technical replicates were averaged and *areg* expression per biological sample was corrected based on *ef1α* expression. One sample was excluded based on the Grubbs outlier test. Significance was determined by a homoscedastic two-tail *t*-test (Fig. 4e) and a one-way Analysis of Variance with Bonferroni’s multiple comparison post-hoc test (Fig. 4f) performed by GraphPad Prism 7 software. P-value less than 0.05 was considered significant. Primers used:

*areg* F: 5′–CTCCTCTTCCTCCGTCTTGC–3′

*areg* R: 5′–GCTGTGGTTTGCTGGGCTAG–3′

*ef1α* F: 5′–AACATCGTGGTCATCGGCCAT–3′

*ef1α* R: 5′–GGAGGTGCCAGTGATCATGTT–3′

### In situ hybridization

Sequences were amplified from cDNA and cloned into pGEM-T-easy vector and sequenced. The specific primers used for *amphiregulin* were 5′–GAAGGTGACAGTTTAAGATCG–3′ and 5′–CCACTTCAAAAATATAAGTGCTTGC–3′. Depending upon orientation in pGEM, T7 or Sp6 polymerase was used to perform in-vitro transcription of the probe. In situ hybridization was performed as previously described^75^ on stylopodial tissue sections collected from juvenile axolotls (approximately 9.5–11.5 c.m. in length) at the time points indicated in Fig. 5a–f. For the analyses in Fig. 5g, h, in situ hybridization was performed on sections of tissue harvested at 6 h following a 4 mm biopsy punch into the flank skin of axolotls. In situ hybridization was performed on tissue sections from adult axolotls at the time points indicated in Supplementary Fig. 4.

### Vector design and delivery

The *amphiregulin* ORF was amplified from cDNA using primers 5′–GGAGAATTCACCGGTGCCACCATGGCTTCTGCCCACTACTCC–3′ and 5′–GCCTGCGGCCGCTCACGCATAAACGTCTCC–3′ (underlined nucleotides bind *areg*’s open reading frame) and cloned into pCAG with EcoRI and NotI to create pCAG-*areg*. The CAG-GFP plasmid was a kind gift from Connie Cepko (Addgene plasmid #11150).^76^ For the *areg*-overexpression vector solution, both pCAG-*areg* and pCAG-*egfp* were diluted to a final concentration of 200 ng/µL in sterile PBS prior to injection (Fast Green dye was added to aid with visualization). The control vector solution consisted of pCAG-*egfp* (200 ng/µL) in sterile PBS (with Fast Green dye added for visualization). Approximately 1.5–2.0 µL of vector solution was injected into each forelimb of juvenile axolotls (4–6 cm in length). Both forelimbs of each axolotl were injected with either control vector solution or *areg*-overexpression solution. Animals were then submerged in 1x PBS, and limbs were electroporated using a NepaGene Super Electroporator NEPA21 Type II electroporator. The poring pulse of our electroporation consisted of 3 pulses at 150 Volts (5 ms pulse length per pulse), a 10 ms pulse interval, a 0% decay rate, and had a positive ( + ) polarity. Our transfer pulse consisted of 5 pulses at 50 Volts (50 ms pulse length per pulse), a 950 ms pulse interval, a 0% decay rate, and had a positive ( + ) polarity. The distance between electrodes was 3 mm for all electroporations. After 5 days of recovery, both forelimbs were amputated at the distal-most region of the stylopodium (just proximal to the elbow) as described above.

### Measuring blastemal length and epidermal/wound epidermal thickness

Animals were anesthetized in 0.1% tricaine and imaged with the ventral aspect of the body upwards. All photos were acquired at the same magnification. Images were quantified using Leica application suite software or ImageJ. Blastemas were measured from the center of the plane of amputation to their distal-most tip. To quantify wound epidermal thickness (Fig. 7a), we measured the width of the central-most part of a section’s wound epidermis using ImageJ. To facilitate comparisons between epidermal thickness in intact limbs (appears as two regions in longitudinal sections) to wound epidermal thickness (one region), we first averaged (mean) the two epidermal layers of longitudinal sections of intact limbs. In the context of limb thickening (Supplementary Fig. 6a–d), we calculated the sum of the two epidermal layers that appear in longitudinal section as both of these layers contribute to the overall width of the limb quantified in Supplementary Fig. 5.

### Skeletal preparations

Alcian blue/alizarin red staining was performed as described previously.^77^

### Statistical analyses

All data are presented as either mean ± sem (Fig. 6, Supplementary Fig. 4, Supplementary Fig. 6) or mean ± standard deviation (Fig. 7). A Fisher’s exact test was used to assess the statistical significance of categorical outcomes between two experiments. For the morphological phenotypic analysis of limbs mis-expressing *areg* (Fig. 6), Fisher’s exact test was performed on a 2 × 5 contingency table. All other analyses involving Fisher’s exact test were performed on 2 × 2 contingency tables. For Fig. 6d, the blastema lengths of the left and right forelimbs were averaged (statistical mean) for each animal at each time point. A two-way, repeated measures ANOVA was performed on the time course blastema data in Fig. 6d, followed by post-hoc pairwise *t*-tests with a Holm correction to adjust for multiple hypothesis testing (used for all pairwise comparisons in Fig. 6d). A two-way ANOVA was performed on the time course epidermal/wound epidermal thickness data in Fig. 7, followed by post-hoc pairwise *t*-tests with a Holm correction to adjust for multiple hypothesis testing. A two-tailed homoscedastic Student’s *t*-test was used to determine whether differences in the percentage of phospho-Histone H3 (ser10) positive nuclei in Fig. 7d, e, percentage of phospho-rpS6 cells in Fig. 7h, i, stylopodium thickness measurements in Supplementary Fig. 5, and measurements in Supplementary Fig. 6c–f were statistically significant. Statistical analyses involving Fisher’s Exact tests, two-way ANOVAs (including repeated measures), and post-hoc pairwise *t*-tests were performed using R; Student’s *t*-tests were performed using Microsoft Excel and Graphpad Prism 7.03. A *p*-value less than 0.05 was considered to be statistically significant.

### Data availability

All expression analysis data has been deposited at GEO, under the identifier GSE103087, for release on September 30, 2017.

## Electronic supplementary material

Supplementary Figure and Table Legends

Supplementary Figure 1

Supplementary Figure 2

Supplementary Figure 3

Supplementary Figure 4

Supplementary Figure 5

Supplementary Figure 6

Supplementary Figure 7

Supplementary Table 1

Supplementary Table 2

Supplementary Table 3

## References

[1] Michalopoulos GK (2007). Liver regeneration. J. Cell. Physiol..

[2] Illingworth CM (1974). Trapped fingers and amputated finger tips in children. J. Pediatr. Surg..

[3] Tanaka EM (2016). The molecular and cellular choreography of appendage regeneration. Cell.

[4] Kragl M (2009). Cells keep a memory of their tissue origin during axolotl limb regeneration. Nature.

[5] Todd JT (1823). On the process of reproduction of the members of the aquatic salamander. Quart. J. Sci. Literature Arts.

[6] Kumar A, Brockes JP (2012). Nerve dependence in tissue, organ, and appendage regeneration. Trends Neurosci..

[7] Liversage RA, McLaughlin DS (1983). Effects of delayed amputation on denervated forelimbs of adult newt. J. Embryol. Exp. Morphol..

[8] Salley JD, Tassava RA (1981). Responses of denervated adult newt limb stumps to reinnervation and reinjury. J. Exp. Zool..

[9] Godwin JW, Pinto AR, Rosenthal NA (2013). Macrophages are required for adult salamander limb regeneration. Proc. Natl Acad. Sci. USA.

[10] Goss RJ (1956). Regenerative inhibition following limb amputation and immediate insertion into the body cavity. Anat. Rec..

[11] Mescher AL (1976). Effects on adult newt limb regeneration of partial and complete skin flaps over the amputation surface. J. Exp. Zool..

[12] Goss RJ (1956). The regenerative responses of amputated limbs to delayed insertion into the body cavity. Anat. Rec..

[13] Suetsugu-Maki R (2012). Lens regeneration in axolotl: new evidence of developmental plasticity. BMC Biol..

[14] Eguchi G (2011). Regenerative capacity in newts is not altered by repeated regeneration and ageing. Nat. Commun.

[15] Monaghan JR (2014). Experimentally induced metamorphosis in axolotls reduces regenerative rate and fidelity. Regeneration.

[16] Yun MH (2015). Changes in regenerative capacity through lifespan. Int. J. Mol. Sci..

[17] Uygur A, Lee RT (2016). Mechanisms of cardiac regeneration. Dev. Cell..

[18] Dearlove GE, Dresden MH (1976). Regenerative abnormalities in *Notophthalmus viridescens* induced by repeated amputations. J. Exp. Zool..

[19] Spallanzani, L. in *An Essay on Animal Reproductions [Prodromo di un opera da imprimersi sopra la riproduzioni anamali]* 68–72 (Becket and de Hondt, London, 1769 [Italian: 1768]).

[20] Bryant DM (2017). Repeated removal of developing limb buds permanently reduces appendage size in the highly-regenerative axolotl. Dev. Biol..

[21] Azevedo AS, Grotek B, Jacinto A, Weidinger G, Saude L (2011). The regenerative capacity of the zebrafish caudal fin is not affected by repeated amputations. PLoS ONE.

[22] Gonzalez-Estevez C (2012). SMG-1 and mTORC1 act antagonistically to regulate response to injury and growth in planarians. PLoS Genet..

[23] Azevedo AS, Sousa S, Jacinto A, Saude L (2012). An amputation resets positional information to a proximal identity in the regenerating zebrafish caudal fin. BMC Dev. Biol..

[24] Morgan, T. H. *Regeneration*. (The Macmillan Company, New York, 1901).

[25] Dalyell, J. G. *Observations on Planariae*. (Archibald Constable, Edinburgh, 1814).

[26] Johnson, J. R. *Further observations on the genus Planaria*. (Royal Society, London, 1825).

[27] Bryant DM (2017). A tissue-mapped axolotl de novo transcriptome enables identification of limb regeneration factors. Cell Rep.

[28] Sousounis K, Athippozhy AT, Voss SR, Tsonis PA (2014). Plasticity for axolotl lens regeneration is associated with age-related changes in gene expression. Regeneration (Oxf).

[29] Mannini L (2004). Djeyes absent (Djeya) controls prototypic planarian eye regeneration by cooperating with the transcription factor Djsix-1. Dev. Biol..

[30] Kawakami Y (2006). Wnt/beta-catenin signaling regulates vertebrate limb regeneration. Genes Dev..

[31] Whited JL, Lehoczky JA, Tabin CJ (2012). Inducible genetic system for the axolotl. Proc. Natl Acad. Sci. USA.

[32] Mahmoud AI (2015). Nerves regulate cardiomyocyte proliferation and heart regeneration. Dev. Cell..

[33] Lipson KE, Wong C, Teng Y, Spong S (2012). CTGF is a central mediator of tissue remodeling and fibrosis and its inhibition can reverse the process of fibrosis. Fibrogenesis Tissue Repair.

[34] Gressner OA, Gressner AM (2008). Connective tissue growth factor: a fibrogenic master switch in fibrotic liver diseases. Liver. Int..

[35] Ihn H (2002). Pathogenesis of fibrosis: role of TGF-beta and CTGF. Curr. Opin. Rheumatol..

[36] Zhou Y (2012). Amphiregulin, an epidermal growth factor receptor ligand, plays an essential role in the pathogenesis of transforming growth factor-beta-induced pulmonary fibrosis. J. Biol. Chem..

[37] Ding L (2016). Bone marrow CD11c+cell-derived Amphiregulin promotes pulmonary fibrosis. J. Immunol..

[38] Perugorria MJ (2008). The epidermal growth factor receptor ligand amphiregulin participates in the development of mouse liver fibrosis. Hepatology..

[39] Ojeh N (2016). The effects of caffeine on wound healing. Int. Wound J..

[40] Stewart R (2013). Comparative RNA-seq analysis in the unsequenced axolotl: the oncogene burst highlights early gene expression in the blastema. PLoS Comput. Biol..

[41] Dreux AC, Lamb DJ, Modjtahedi H, Ferns GA (2006). The epidermal growth factor receptors and their family of ligands: their putative role in atherogenesis. Atherosclerosis.

[42] Shao J, Sheng H (2010). Amphiregulin promotes intestinal epithelial regeneration: roles of intestinal subepithelial myofibroblasts. Endocrinology.

[43] Berasain C (2005). Amphiregulin: an early trigger of liver regeneration in mice. Gastroenterology.

[44] Schneider MR, Werner S, Paus R, Wolf E (2008). Beyond wavy hairs: the epidermal growth factor receptor and its ligands in skin biology and pathology. Am. J. Pathol..

[45] Liou A, Elias PM, Grunfeld C, Feingold KR, Wood LC (1997). Amphiregulin and nerve growth factor expression are regulated by barrier status in murine epidermis. J. Invest. Dermatol..

[46] Cook PW, Brown JR, Cornell KA, Pittelkow MR (2004). Suprabasal expression of human amphiregulin in the epidermis of transgenic mice induces a severe, early-onset, psoriasis-like skin pathology: expression of amphiregulin in the basal epidermis is also associated with synovitis. Exp. Dermatol..

[47] Bhagavathula N (2005). Amphiregulin and epidermal hyperplasia: amphiregulin is required to maintain the psoriatic phenotype of human skin grafts on severe combined immunodeficient mice. Am. J. Pathol..

[48] Li Y (2016). Transgenic expression of human amphiregulin in mouse skin: inflammatory epidermal hyperplasia and enlarged sebaceous glands. Exp. Dermatol..

[49] Cook PW (1997). Transgenic expression of the human amphiregulin gene induces a psoriasis-like phenotype. J. Clin. Invest..

[50] Tassava RA, Johnson-Wint B, Gross J (1986). Regenerate epithelium and skin glands of the adult newt react to the same monoclonal antibody. J. Exp. Zool..

[51] Shoyab M, Plowman GD, McDonald VL, Bradley JG, Todaro GJ (1989). Structure and function of human amphiregulin: a member of the epidermal growth factor family. Science.

[52] Farkas JE, Freitas PD, Bryant DM, Whited JL, Monaghan JR (2016). Neuregulin-1 signaling is essential for nerve-dependent axolotl limb regeneration. Development.

[53] Castilho RM, Squarize CH, Gutkind JS (2013). Exploiting PI3K/mTOR signaling to accelerate epithelial wound healing. Oral Dis..

[54] Zarogoulidis P (2014). mTOR pathway: a current, up-to-date mini-review (Review). Oncol. Lett..

[55] Hirose K, Shiomi T, Hozumi S, Kikuchi Y (2014). Mechanistic target of rapamycin complex 1 signaling regulates cell proliferation, cell survival, and differentiation in regenerating zebrafish fins. BMC Dev. Biol..

[56] Jazwinska A, Sallin P (2016). Regeneration versus scarring in vertebrate appendages and heart. J. Pathol..

[57] Gurtner GC, Werner S, Barrandon Y, Longaker MT (2008). Wound repair and regeneration. Nature.

[58] Chung E (2005). Amphiregulin causes functional downregulation of adherens junctions in psoriasis. J. Invest. Dermatol..

[59] Morhenn VB, Nelson TE, Gruol DL (2013). The rate of wound healing is increased in psoriasis. J. Dermatol. Sci..

[60] Skowron S, Komala Z (1957). Limb regeneration in postmetamorphic Xenopus laevis. Folia Biol Krakow.

[61] Yun, M. H., Davaapil, H. & Brockes, J. P. Recurrent turnover of senescent cells during regeneration of a complex structure. *Elife* **4**, 10.7554/eLife.05505 (2015). 10.7554/eLife.05505 PMC4434796 25942455

[62] Thompson S, Muzinic L, Muzinic C, Niemiller ML, Voss SR (2014). Probability of regenerating a normal limb after bite Injury in the Mexican Axolotl (Ambystoma mexicanum). Regeneration.

[63] Schneider CA, Rasband WS, Eliceiri KW (2012). NIH Image to ImageJ: 25 years of image analysis. Nat. Methods.

[64] Grabherr MG (2011). Full-length transcriptome assembly from RNA-Seq data without a reference genome. Nat. Biotechnol..

[65] Haas BJ (2013). De novo transcript sequence reconstruction from RNA-seq using the Trinity platform for reference generation and analysis. Nat. Protoc..

[66] Camacho C (2009). BLAST+: architecture and applications. BMC Bioinformatics.

[67] Boeckmann B (2005). Protein variety and functional diversity: Swiss-Prot annotation in its biological context. C. R. Biol..

[68] Finn RD (2014). Pfam: the protein families database. Nucleic Acids Res..

[69] Li B, Dewey CN (2011). RSEM: accurate transcript quantification from RNA-Seq data with or without a reference genome. BMC Bioinform.

[70] Robinson MD, McCarthy DJ, Smyth GK (2010). edgeR: a Bioconductor package for differential expression analysis of digital gene expression data. Bioinformatics.

[71] de Hoon MJ, Imoto S, Nolan J, Miyano S (2004). Open source clustering software. Bioinformatics.

[72] Saldanha AJ (2004). Java Treeview–extensible visualization of microarray data. Bioinformatics.

[73] Maere S, Heymans K, Kuiper M (2005). BiNGO: a Cytoscape plugin to assess overrepresentation of gene ontology categories in biological networks. Bioinformatics.

[74] Supek F, Bosnjak M, Skunca N, Smuc T (2011). REVIGO summarizes and visualizes long lists of gene ontology terms. PLoS ONE.

[75] Whited JL, Lehoczky JA, Austin CA, Tabin CJ (2011). Dynamic expression of two thrombospondins during axolotl limb regeneration. Dev. Dyn..

[76] Matsuda T, Cepko CL (2004). Electroporation and RNA interference in the rodent retina in vivo and in vitro. Proc. Natl Acad. Sci. USA.

[77] Whited JL (2013). Pseudotyped retroviruses for infecting axolotl in vivo and in vitro. Development.

