# Supplementary material for: Identification of regenerative roadblocks via repeat deployment of limb regeneration in axolotls
Source: NPJ Regen Med. 2017 Nov 6;2:30. doi: 10.1038/s41536-017-0034-z (PMC5677943; doi:10.1038/s41536-017-0034-z)
Supplement: Supplementary file 1 — Supplementary Figure and Table Legends [file 41536_2017_34_MOESM1_ESM.docx]

**Supplementary Figure 1. Experimental design and digit distributions of regenerated limbs following repeated amputation in the same plane or repeated serially distal amputation.** Both forelimbs were amputated for each animal and allowed to fully regenerate. a) Schematic showing how the amputation plane was maintained as animals aged and grew. Red dotted line indicates amputation plane; arrowheads indicate the mid-humerus mark, evident by visible ossification center. b) Three representative samples partway through the amputation-regeneration protocol showing the boundary between stump skin and regenerate skin (asterisks), which is coincident with the future amputation plane. c) Quantification of the number of digits axolotl limbs were able to regenerate after each amputation round following repeated amputation in the same plane. d) Quantification of the number of digits axolotl limbs were able to regenerate after each amputation round following repeated, progressively distal amputations. Scale bars are 1 mm.

**Supplementary Figure 2. Aberrant expression of Collagen I and Collagen IV in stumps from failed regenerates.** Shown are tissue sections from controls/intact limbs and failed regenerate limbs. a-a’) α-Collagen I (green), DAPI (nuclei, blue), and phalloidin (F-actin, red) in control (a) and failed regenerates (a’). b-b’) α-Collagen IV (red) and DAPI (nuclei, blue) in control (b) and failed regenerates (b’). Epi is epidermis, sc is stratum compactum (arrowhead). Scale bars are 100 µm.

**Supplementary Figure 3. *Amphiregulin* is expressed early during normal limb regeneration.** Shown is a scatterplot of *areg*’s temporal expression pattern at different time points during axolotl limb regeneration. Data are expressed as Transcripts Per kilobase Million (TPM). Expression data was obtained from ^40^.

**Supplementary Figure 4. *Amphiregulin* expression in failed regenerates.** *In situ* hybridization analyses of *areg* expression in control limbs and failed regenerates of adult axolotls. a) Expression of *areg* in intact control limb, b) control limb at 12 hours post-amputation, and c) control limb at 3 days post-amputation. d) Expression of *areg* in failed regenerate limb prior to amputation, e) failed regenerate limb at 12 hours post-amputation, and f) failed regenerate limb at 3 days post-amputation. Arrowheads indicate expression of *areg* in the leading edge of the wound epidermis. Epi = epidermis; WE = wound epidermis; arrowhead indicates leading edge of wound epidermis. Double arrowheads indicate epidermal tongue. Scale bars are 100 µm.

**Supplementary Figure 5. Overexpression of *amphiregulin* leads to enlarged limbs.** Axolotl limbs with no prior injuries were electroporated with either plasmid encoding either GFP (control) or plasmids encoding GFP plus AREG. a) Representative whole mount images of GFP expression in the stylopodium of control limbs at 5 days post-electroporation. b) Representative whole mount images of GFP expression in the stylopodium of *areg* mis-expressing limbs at 5 days post-electroporation. c) Representative tissue section images of GFP expression in the stylopodium of control limbs at 5 days post-electroporation. Sections were stained with anti-AREG antibodies. “Epi” refers to epidermis. d) Representative tissue section images of GFP and *areg* expression in the stylopodium of *areg* mis-expressing limbs at 5 days post-electroporation. Sections were stained with anti-AREG antibodies. e) Representative images of control GFP overexpressing forelimbs and f) non-electroporated hindlimbs from the same animal at 5 days post-electroporation. g) Representative images of *areg* overexpressing forelimbs and h) non-electroporated hindlimbs from the same animal at 5 days post-electroporation. i) Quantification of stylopodial thickness and j) stylopodial index (ratio of forelimb stylopodium to hindlimb stylopodium; bottom graph) in panels e-h. ***indicates p<0.001. N=24 animals for control, and N=23 animals for *areg* overexpression. Scale bars in panels a,b and e-h are 1 mm. Scale bars in panels c and d are 100 µm.

**Supplementary Figure 6.** **Consequences of ectopic *areg* expression in intact limbs.** Intact, unamputated control limbs (electroporated with plasmid encoding GFP) and AREG-overexpressing limbs (electroporated with plasmids encoding AREG and GFP) were harvested and analyzed 5 days post-electroporation. a-b) Representative tissue sections stained with DAPI (nuclei) and α-pH3 (mitotically active cells). Scale bars are 200 µm. c-d) Quantification of thickness of internal tissues (c) and thickness of epidermis (d) in control versus AREG-overexpressing limbs (n=5 limbs per condition). e-f) Quantification of fraction of DAPI nuclei that are also pH3+ in internal tissues (e) and epidermis (f) in control versus AREG-overexpressing limbs (n=5 limbs per condition). *** denotes p<0.001; n.s. denotes not significant.

**Supplementary Figure 7.** **Expression of wound epidermis marker WE3 in epidermis covering limbs overexpressing *areg* at 8 dpa.** Limbs were electroporated with plasmids encoding GFP (control, panel a) or GFP plus AREG (+AREG, panel b), amputated 5 days later through the expression domain, and harvested 8 days post-amputation. DAPI is blue, WE3 is red. Scale bars are 100 µm.

**Supplementary Table 1.** FPKM matrix of transcripts differentially expressed between repeatedly amputated failed regenerates and sibling control limbs at 3 days post-amputation. Samples were processed as biological quadruplicates for each condition, and the FPKM values were averaged.

**Supplementary Table 2.** Full list of significantly enriched Gene Ontology terms (Biological Processes) for comparisons between repeatedly amputated failed regenerates and sibling control limbs at 3 days post-amputation.

**Supplementary Table 3.** Full description of morphological outcomes at ~8 weeks post-amputation following *amphiregulin* mis-expression. Data for each group were sorted according to morphological outcome.
