# Supplementary figures and images for: Identification of regenerative roadblocks via repeat deployment of limb regeneration in axolotls
Source: NPJ Regen Med. 2017 Nov 6;2:30. doi: 10.1038/s41536-017-0034-z (PMC5677943; doi:10.1038/s41536-017-0034-z)

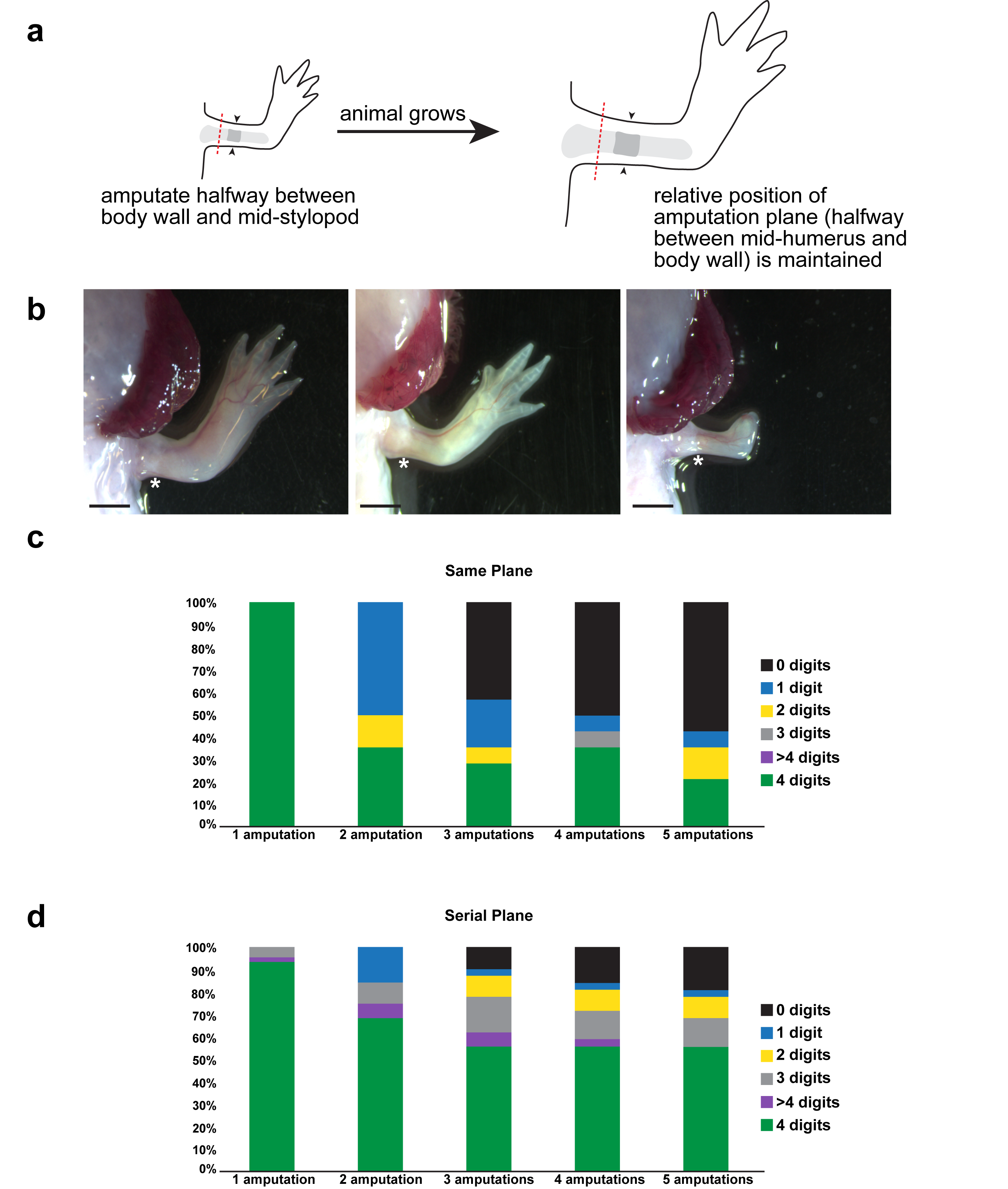

Supplement: Supplementary file 2 — Supplementary Figure 1 [file 41536_2017_34_MOESM2_ESM.tif]

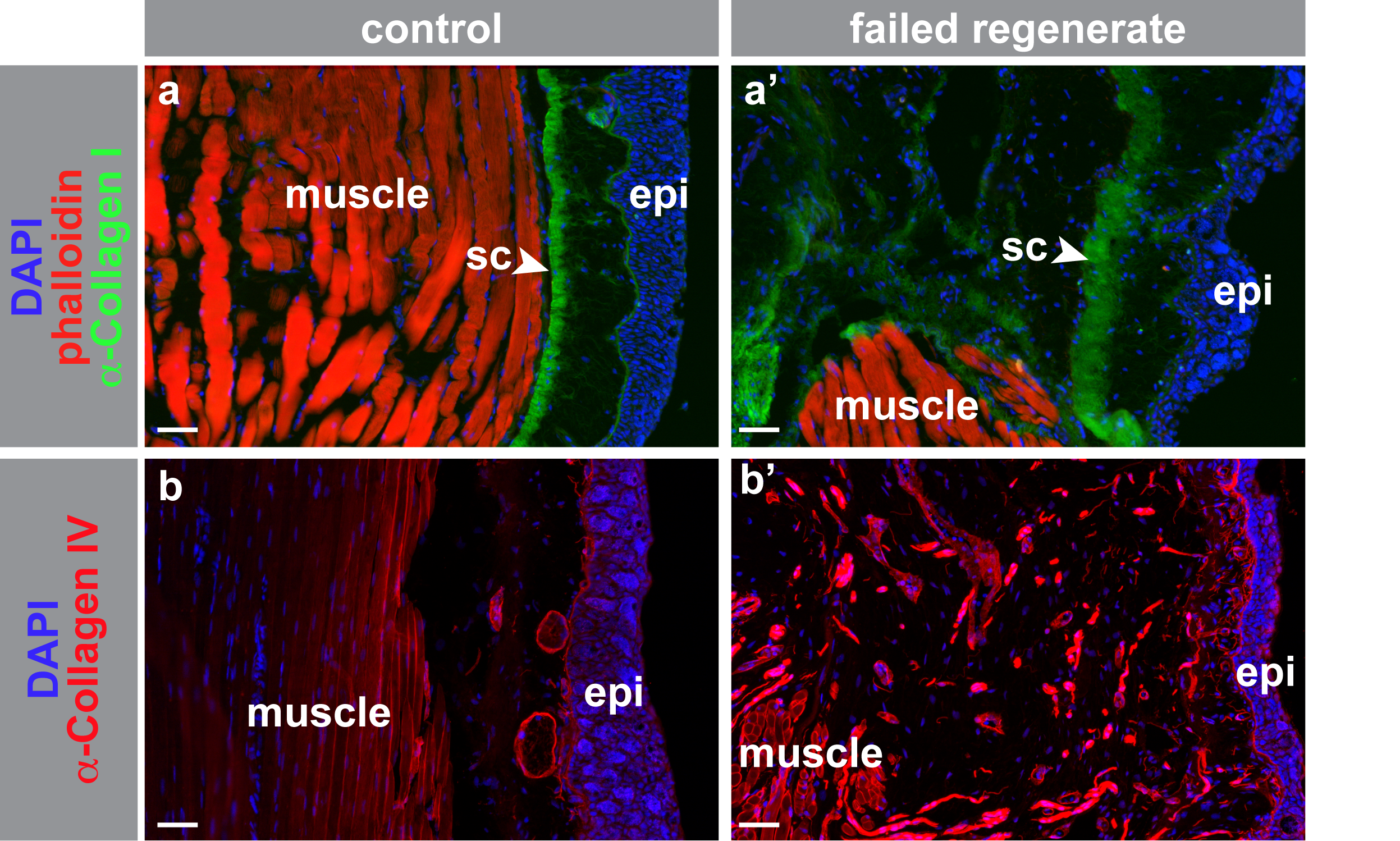

Supplement: Supplementary file 3 — Supplementary Figure 2 [file 41536_2017_34_MOESM3_ESM.tif]

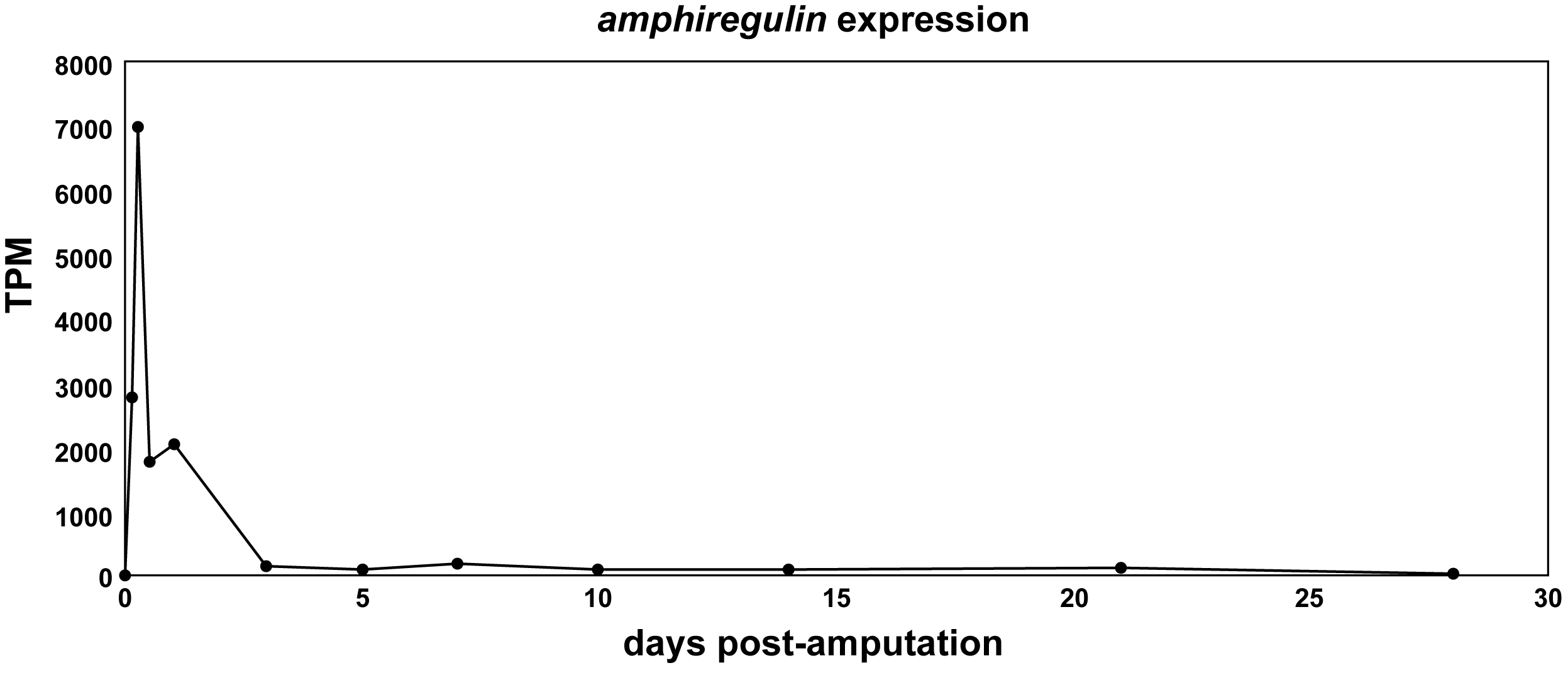

Supplement: Supplementary file 4 — Supplementary Figure 3 [file 41536_2017_34_MOESM4_ESM.tif]

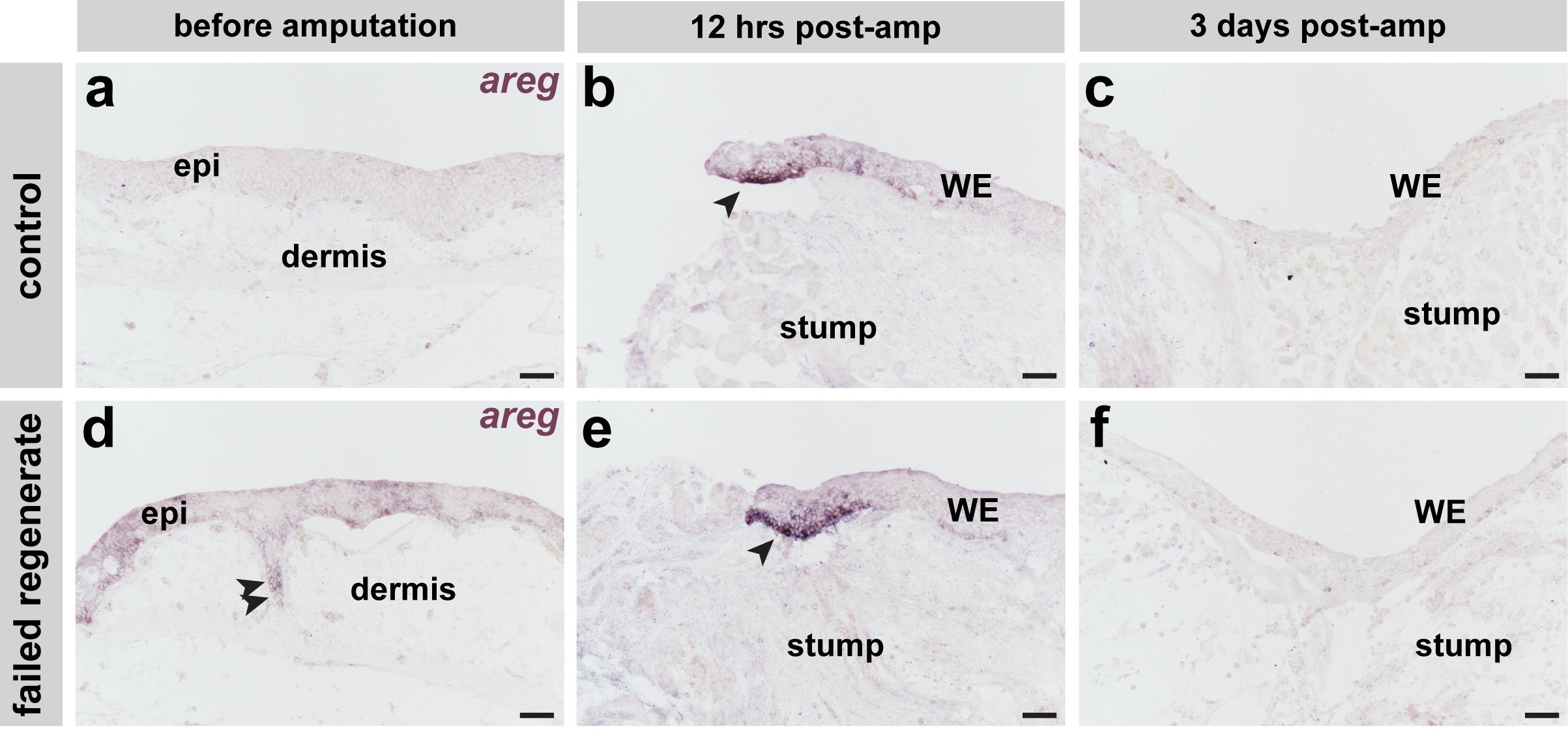

Supplement: Supplementary file 5 — Supplementary Figure 4 [file 41536_2017_34_MOESM5_ESM.tif]

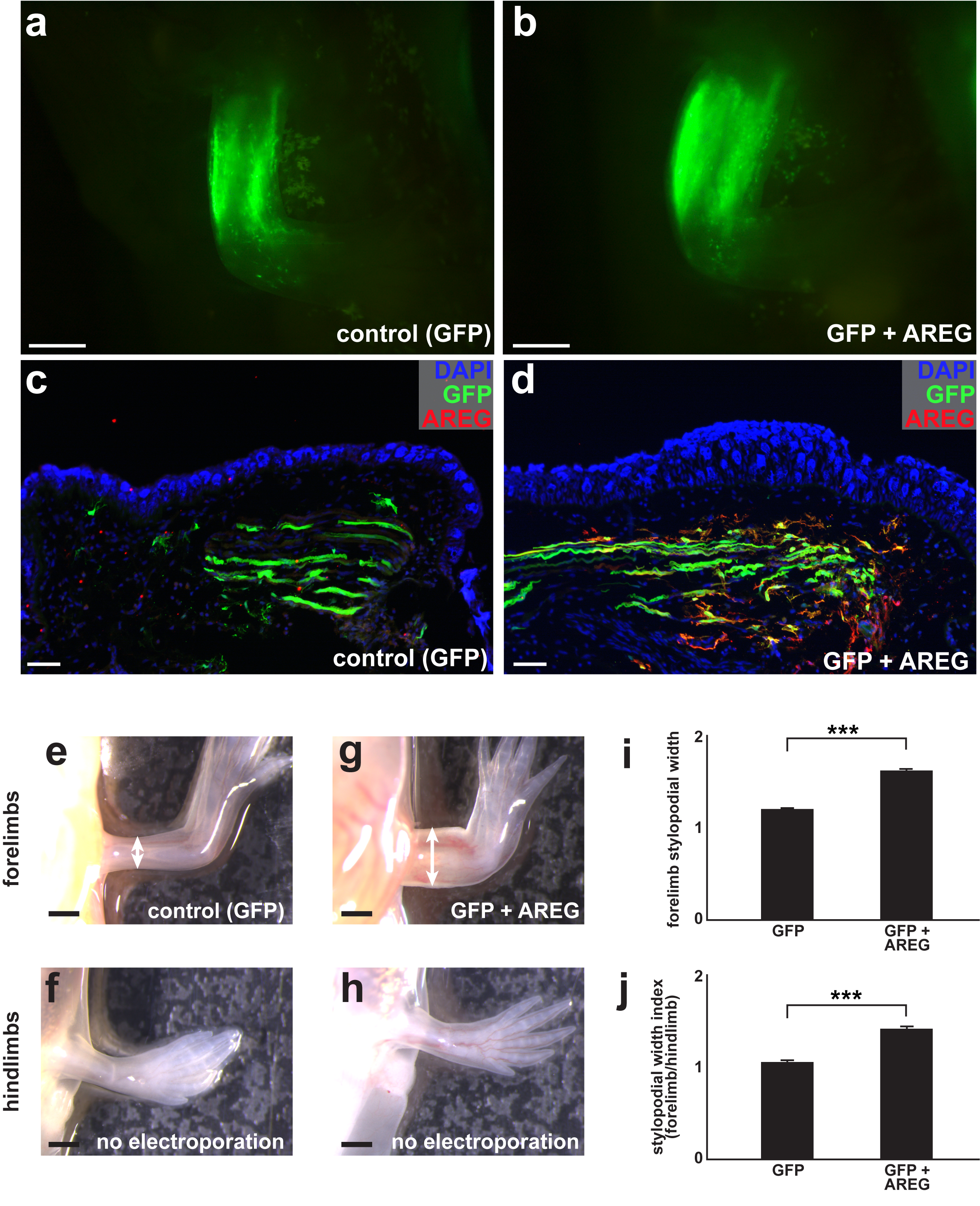

Supplement: Supplementary file 6 — Supplementary Figure 5 [file 41536_2017_34_MOESM6_ESM.tif]

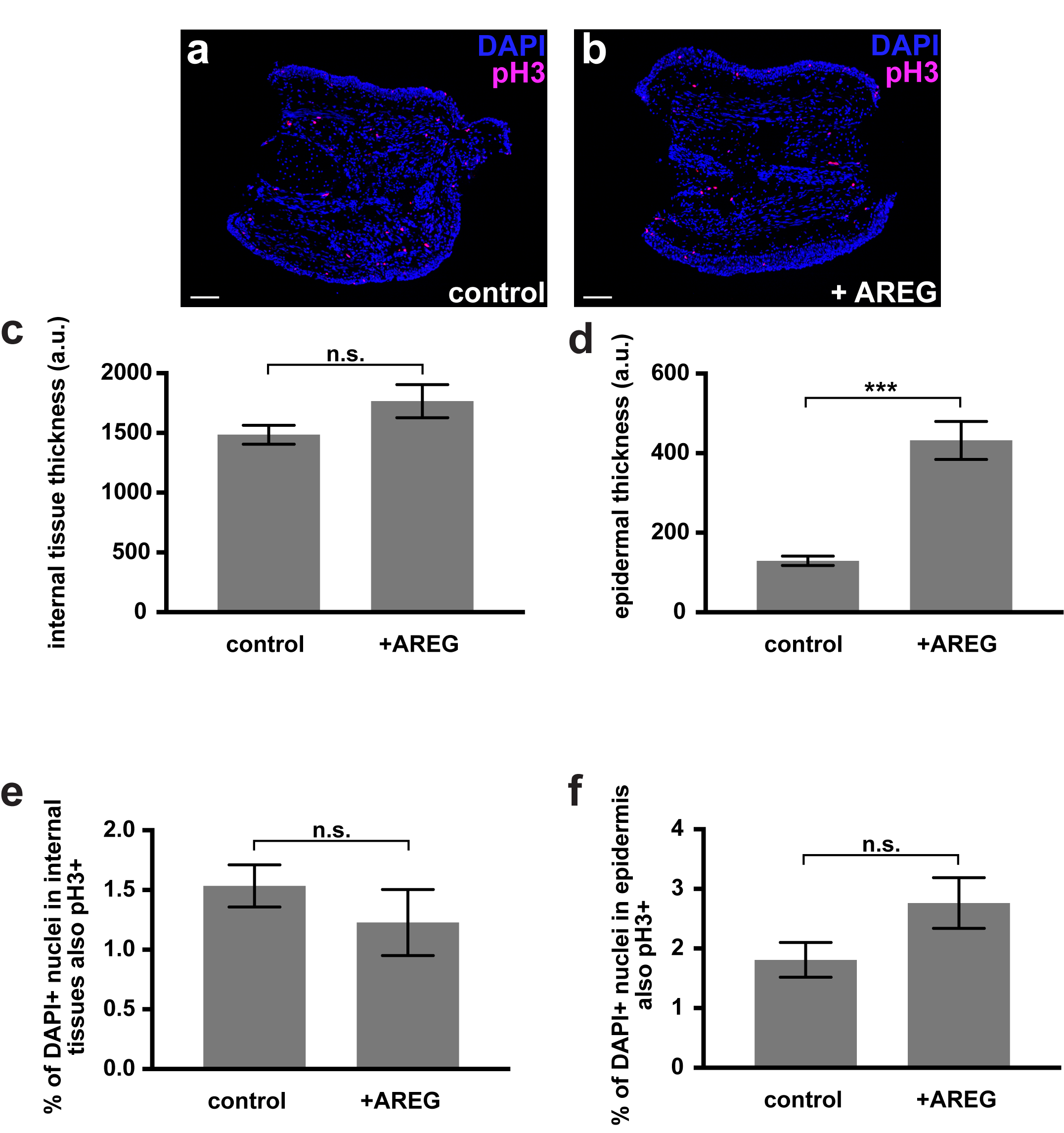

Supplement: Supplementary file 7 — Supplementary Figure 6 [file 41536_2017_34_MOESM7_ESM.tif]

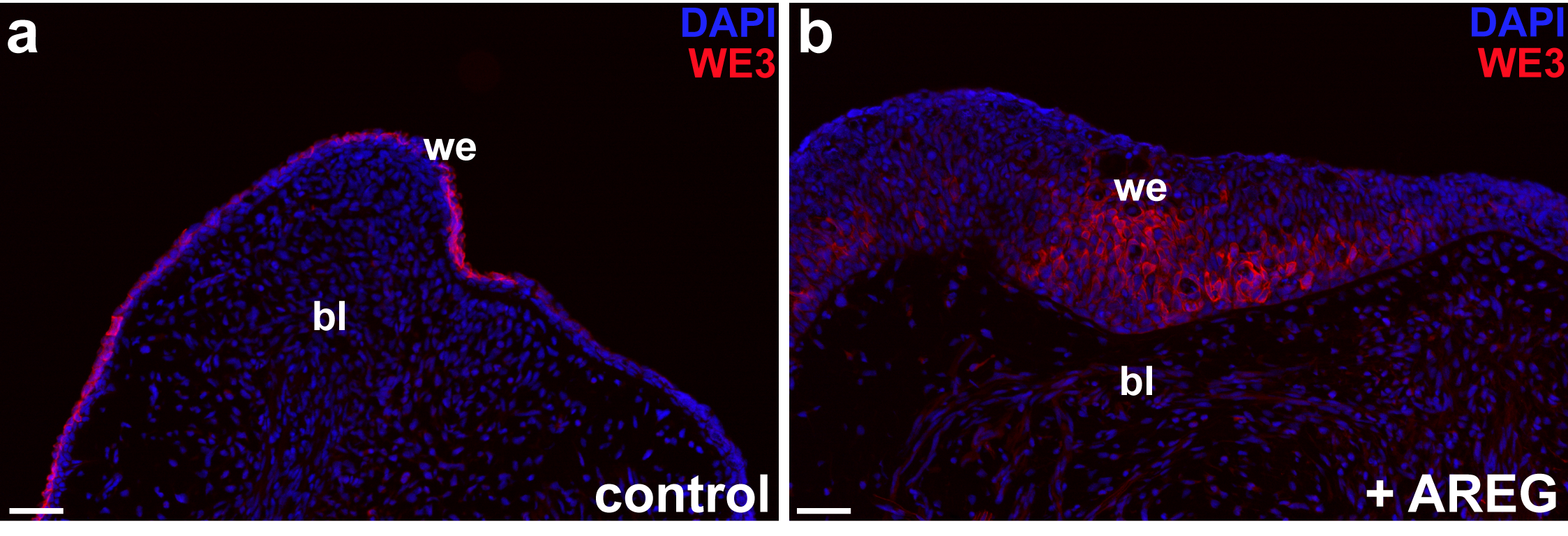

Supplement: Supplementary file 8 — Supplementary Figure 7 [file 41536_2017_34_MOESM8_ESM.tif]
