# Supplementary material for: Identification of regenerative roadblocks via repeat deployment of limb regeneration in axolotls
Source: NPJ Regen Med. 2017 Nov 6;2:30. doi: 10.1038/s41536-017-0034-z (PMC5677943; doi:10.1038/s41536-017-0034-z)
Supplement: Supplementary file 9 — Supplementary Table 1 [file 41536_2017_34_MOESM9_ESM.pdf]

Gene (contig or protein I.D.)	Single Amputation mean FPKM	Repeated Amputation mean FPKM	Fold Change (Repeated/Single)
TR29920|c0_g1_i1	1	123.5985	123.5985
TR6990|c0_g1_i1	1	55.186	55.186
TR15140|c17_g1_i1	5.4185	190.341	35.12798745
TELT_MOUSE	3.3105	95.39275	28.81520918
TR15140|c16_g1_i1	3.81375	106.39975	27.89898394
TR29077|c20_g1_i1	1	24.5315	24.5315
TRI27_HUMAN	1.01475	23.8185	23.47228381
TR387|c0_g1_i1	4.366	89.62725	20.52845854
MYH4_PIG	10.64625	211.721	19.88690854
XIRP1_CHICK	2.72975	52.1575	19.10706109
TR15140|c25_g1_i1	2.3635	44.885	18.99090332
MLRV_HUMAN	4.365	81.0995	18.57949599
HSPBB_DANRE	11.4255	204.41475	17.89109886
MYH7_PAPHA	3.71	64.67675	17.43308625
MYH8_RAT	4.02175	66.25025	16.47299061
MYSS_RABIT	4.211	65.969	15.66587509
RAD_MOUSE	2.4955	38.6035	15.46924464
GNA1_PONAB	1	15.44625	15.44625
AT2A1_PELES	4.50375	69.11025	15.3450458
TELT_MOUSE	8.85475	135.54525	15.3076315
MYH7_PIG	2.85925	41.53475	14.52644924
TR3471|c0_g1_i1	1.33075	19.24575	14.46233327
TPM3_BOVIN	7.41675	106.6555	14.38035528
TR15140|c19_g1_i1	1.3215	18.88075	14.28736285
TNNT3_RAT	5.59675	79.29825	14.16862465
TNNT1_PIG	6.17375	86.0365	13.93585746
TR20028|c2_g1_i1	1	13.77925	13.77925
MLEC_CHICK	11.745	159.57125	13.58631332
MYH4_HUMAN	2.6015	35.2545	13.55160484
CSRP3_MOUSE	6.361	85.86475	13.49862443
MYH3_MOUSE	2.7965	37.3525	13.35687466
MYH2_BOVIN	3.7365	49.75225	13.31520139
TRI63_MOUSE	2.5955	34.312	13.21980351
MURC_XENLA	2.25325	29.111	12.91956063
ANKR1_XENLA	13.13775	169.583	12.90807026
TNNI1_RABIT	3.97925	50.54875	12.70308475
TPM3_BOVIN	6.8615	86.5865	12.61917948
HSPB7_HUMAN	3.7115	46.591	12.55314563
2ABA_RABIT	1	12.514	12.514
RGCC_RAT	3.0525	37.57075	12.30819001
CK096_MOUSE	4.30675	52.5345	12.19817728
TNNC1_PIG	8.5615	103.372	12.07405244
SARCO_RAT	2.18825	26.3665	12.04912601
PDLI7_BOVIN	3.15	37.373	11.86444444
DESM_XENLA	9.3535	110.43825	11.80715775
MYH7_PIG	2.78575	32.821	11.78174639

TITIN_HUMAN	2.9885	34.47225	11.53496737
NEBU_HUMAN	2.215	25.437	11.48397291
DESM_XENLA	18.0285	206.7455	11.46770391
MYH7_CANFA	2.58325	29.2745	11.33243008
MLRB_CHICK	30.4405	344.2225	11.30804356
TR8209|c0_g2_i1	3.08975	34.508	11.16854114
FLNC_HUMAN	1.4615	15.98875	10.93995895
GIMA4_HUMAN	1.98025	20.8895	10.54892059
LMOD2_HUMAN	1.5435	15.76875	10.21622935
TRFE_ANAPL	1.43675	14.2635	9.927614408
TR2143|c0_g1_i1	1.076	10.192	9.472118959
FLNC_HUMAN	4.13525	37.5825	9.088325978
PRVB_XENLA	1.87675	16.667	8.880777941
ADT1_RABIT	5.7165	50.483	8.831102948
TR15147|c7_g1_i1	3.79575	33.03525	8.703220707
FLNC_RAT	5.1295	44.02325	8.582366702
MYH6_RAT	1.0215	8.73775	8.553842389
KAD1_PIG	8.745	74.66675	8.538221841
TR26647|c0_g1_i1	1.61775	13.655	8.44073559
TPM1_XENLA	40.10925	327.23325	8.158548215
F16P2_RABIT	1.5685	12.6675	8.07618744
PFKAM_BOVIN	2.009	16.22375	8.075535092
TR26414|c2_g2_i1	3.5735	28.81825	8.064432629
MYH6_MOUSE	1	7.912	7.912
MYPC1_HUMAN	1.31225	10.2855	7.838064393
XIRP1_CHICK	1.24025	9.55075	7.700665188
KLH34_HUMAN	1.258	9.5635	7.602146264
LMOD3_HUMAN	1.43675	10.82175	7.532103706
MYOTI_HUMAN	1.419	10.50825	7.405391121
TR11059|c0_g1_i1	2.3285	17.15275	7.366437621
MPSF_CHICK	1.1955	8.78325	7.346925972
MYH6_MOUSE	1.09375	8.0265	7.338514286
MUC5B_HUMAN	2.4015	17.48575	7.28117843
TR5659|c26_g1_i1	1.5975	11.6155	7.271048513
HARB1_HUMAN	2.141	14.97725	6.995446053
TR24904|c0_g1_i1	1.4725	10.2945	6.991171477
NEBU_HUMAN	1.6815	11.6595	6.933987511
MUC5A_HUMAN	3.23775	22.441	6.931047796
MLF1_BOVIN	2.146	14.8145	6.903308481
TRI72_XENLA	2.3265	15.909	6.838168923
TR26611|c9_g2_i1	1	6.74775	6.74775
MYOZ2_HUMAN	1.3515	9.1145	6.743988161
SRCA_CHICK	1.89975	12.7835	6.729043295
MYH6_MESAU	1	6.66475	6.66475
NRAP_HUMAN	1.84875	12.30875	6.657876944
TRFEA_XENLA	11.04025	73.3885	6.647358529
PURA1_XENTR	1.4015	9.2955	6.632536568

TR29757|c1_g1_i1	3.7735	24.8985	6.598250961
MYH6_HUMAN	1	6.5745	6.5745
MYPC3_CHICK	1.60975	10.544	6.550085417
MSTN1_BOVIN	3.7795	24.731	6.543458129
NEBU_HUMAN	1.41675	9.21575	6.504852656
TR15205|c7_g1_i1	2.926	19.02225	6.501110731
MYH6_HUMAN	1.06125	6.8515	6.45606596
MYSC_CHICK	1.3105	8.4475	6.446012972
MYL4_HUMAN	2.12425	13.43725	6.325644345
MYH7_BOVIN	1.083	6.83	6.306555863
MYH6_MOUSE	1	6.28875	6.28875
TR15205|c7_g2_i1	2.2685	14.13975	6.233083535
NRAP_HUMAN	1.97575	12.30925	6.23016576
TR1795|c0_g1_i1	1.505	9.35225	6.214119601
TR20012|c3_g2_i1	1.13475	6.9485	6.123375193
TR6410|c2_g1_i7	1.2965	7.89575	6.090050135
TR5659|c23_g1_i1	1.531	9.161	5.983670803
TR20012|c1_g1_i1	1	5.98225	5.98225
TR5659|c3_g2_i1	1.40175	8.382	5.979668272
PDLI3_XENLA	2.2905	13.6685	5.967474351
SGCE_PONAB	1.61625	9.613	5.947718484
TR2965|c1_g1_i1	1.87175	11.09325	5.9266729
TR26411|c9_g1_i1	1.0515	6.0975	5.798858773
TR24947|c0_g2_i1	1.187	6.81025	5.7373631
TR26411|c20_g1_i1	1.11525	6.378	5.718897108
TR16529|c6_g1_i1	2.75425	15.74975	5.718344377
TELT_BOVIN	1.11275	6.3315	5.689957313
SYP2L_MOUSE	1.084	6.154	5.677121771
NEBU_HUMAN	1.38225	7.79875	5.64206909
CASQ2_CHICK	1.27575	7.134	5.592004703
NAR1_MOUSE	1.456	8.06275	5.537603022
FUCL4_ANGJA	1.21975	6.643	5.446197991
TR18740|c24_g1_i1	1	5.3965	5.3965
TR26411|c24_g1_i1	1.0565	5.6905	5.386180786
RTN2_MOUSE	1.94425	10.42475	5.361836184
TR20012|c3_g1_i1	1.108	5.87875	5.305731047
TR18710|c2_g1_i1	1.27325	6.7465	5.298645199
KLH34_HUMAN	1.07675	5.6605	5.25702345
TR30767|c0_g1_i1	1.12025	5.87875	5.247712564
TR5653|c1_g1_i1	3.20375	16.72575	5.220678892
TR11059|c1_g1_i1	1.703	8.883	5.216089254
MLF1_PONAB	1.77625	9.26425	5.215622801
VDHAP_CHICK	2.003	10.32175	5.153145282
RYR1_RAT	1.125	5.79475	5.150888889
TR10077|c22_g1_i1	1.0565	5.43125	5.140795078
ACTN2_MOUSE	1.48375	7.60175	5.123336142
PPR27_MOUSE	1.137	5.78575	5.088610378

TR4340|c0_g2_i2	1.09325	5.557	5.083009376
CAV3_MOUSE	1.40775	7.14875	5.078138874
COX6A_ONCMY	1.5145	7.6445	5.047540442
TR25406|c0_g1_i1	1	5.0435	5.0435
TR29077|c3_g1_i2	1	5.04025	5.04025
ACTN2_HUMAN	1.46225	7.3695	5.039835869
SMTL2_BOVIN	1.7125	8.62475	5.036350365
TR25198|c0_g1_i1	1.56875	7.8085	4.97752988
NEBU_HUMAN	1.37125	6.8065	4.963719234
MPSF_CHICK	1.3555	6.7245	4.960900037
MYOG_CHICK	1.6065	7.95375	4.950980392
TR1170|c1_g1_i1	1.18875	5.87475	4.941955836
TR8074|c4_g1_i1	1	4.9095	4.9095
DRS7C_XENTR	1.4125	6.86575	4.860707965
TR23021|c2_g1_i1	1.6795	8.11825	4.833730277
TPM1_LIZAU	1.55	7.45325	4.808548387
MYH6_MOUSE	1.26125	6.0355	4.785332012
OTOSP_HUMAN	1.5115	7.18825	4.755706252
CO9A2_MOUSE	1.0215	4.82625	4.724669604
TR18709|c0_g1_i1	1	4.67875	4.67875
PBIP1_BOVIN	2.22675	10.35275	4.649264623
TERA_XENTR	1.96625	9.135	4.645899555
TR30024|c0_g3_i1	3.77925	17.45075	4.617516703
CO9A1_MOUSE	1.0545	4.862	4.610715979
TR5694|c2_g1_i1	1	4.591	4.591
PHLA3_RAT	1	4.586	4.586
CO9A2_MOUSE	1.14375	5.22375	4.567213115
CDC16_HUMAN	1	4.563	4.563
CO2A1_CHICK	1.2795	5.82825	4.555099648
MUC5B_HUMAN	1.52775	6.8885	4.508918344
TR15141|c16_g1_i1	1	4.493	4.493
COF2_XENLA	7.94325	35.409	4.457747144
MACD1_BOVIN	1.7635	7.84475	4.448398072
CTNS_HUMAN	1	4.43725	4.43725
TRDN_RAT	1.1645	5.16525	4.435594676
TR18676|c11_g1_i1	1.0325	4.57225	4.428329298
MYH7B_HUMAN	1.1715	5.1805	4.422108408
SDHL_MOUSE	1.54125	6.80225	4.413463098
TERA_XENTR	2.11875	9.27175	4.376047198
TR26614|c1_g1_i1	1.30525	5.67825	4.350316031
TM182_XENTR	1.4965	6.47975	4.329936519
KITH_CHICK	1	4.317	4.317
TR24947|c2_g1_i1	1.28925	5.55875	4.31161528
TR3033|c4_g2_i1	1.14225	4.923	4.309914642
TR24348|c1_g1_i1	1	4.29975	4.29975
SRCH_HUMAN	1.32825	5.68725	4.281761717
TR22643|c2_g2_i1	5.4325	23.22275	4.274781408

KLH31_HUMAN	1.17725	4.99675	4.244425568
TR30024|c0_g2_i1	3.377	14.26975	4.225570033
TR8322|c0_g2_i1	1.233	5.19325	4.21188159
RYR1_MOUSE	1.19825	4.9975	4.170665554
PLEK2_MOUSE	12.36725	51.5425	4.167660555
MYH7B_HUMAN	1.0735	4.4435	4.139264089
FABPH_RAT	4.224	17.4405	4.12890625
MYBPH_CHICK	1.29425	5.312	4.104307514
DUPD1_XENTR	1.155	4.72175	4.088095238
CORO6_RAT	2.03725	8.318	4.082954964
TERA_DANRE	1.86325	7.586	4.071380652
MYH7B_MOUSE	1.12825	4.58275	4.061821405
TR5093|c1_g2_i1	1.53275	6.186	4.035883216
TR29029|c15_g1_i1	1.1125	4.47675	4.024044944
XIRP2_HUMAN	1.09325	4.3685	3.995883833
FHL1_RAT	8.70525	34.77075	3.994227621
TR1193|c1_g1_i1	1.6025	6.38625	3.985179407
CTGF_BOVIN	12.157	48.41825	3.982746566
MYPC1_HUMAN	1.19275	4.731	3.966464054
CASQ2_MOUSE	1.29925	5.11725	3.938618434
TR10851|c6_g1_i1	1	3.933	3.933
CO9A3_CHICK	1.0185	3.9775	3.905252823
AREG_HUMAN	8.683	33.78625	3.891080272
TR22602|c10_g1_i1	1.0565	4.10775	3.888073829
MYH7B_MOUSE	1.12375	4.348	3.869187987
TR11743|c4_g1_i1	1.331	5.1195	3.846356123
MAON_MOUSE	1	3.843	3.843
LFG1_HUMAN	1.20375	4.6005	3.821806854
SMYD1_MOUSE	1.1005	4.15525	3.775783735
AIF1L_MOUSE	5.523	20.80025	3.766114431
TR18692|c32_g1_i1	1.67725	6.316	3.765687882
TR29748|c1_g1_i1	2.1075	7.919	3.757532622
CD109_HUMAN	1	3.713	3.713
OBSCN_HUMAN	1.199	4.45175	3.712885738
TR15121|c23_g1_i1	1.3	4.82425	3.710961538
TR10008|c3_g1_i1	1.3965	5.16925	3.701575367
TR16116|c0_g4_i1	4.131	15.2875	3.700677802
DS22A_DANRE	1.1235	4.15075	3.694481531
TR24046|c3_g1_i1	1.58975	5.85175	3.680924674
TR20012|c2_g1_i1	1.054	3.8795	3.680740038
TR26330|c2_g2_i1	1.081	3.948	3.652173913
TR2965|c3_g1_i1	1.3055	4.74475	3.634431252
HACD1_HUMAN	1.7105	6.2015	3.625548085
ABRA_HUMAN	1.152	4.15925	3.610460069
MSS51_HUMAN	1.2885	4.648	3.607295305
TR16212|c0_g1_i1	1.16125	4.18875	3.607104413
LRRN1_BOVIN	1.01875	3.6685	3.600981595

CTDS2_HUMAN	1	3.59375	3.59375
TR12405|c4_g1_i1	1.3235	4.7455	3.585568568
TXLNB_CHICK	1.19625	4.28625	3.5830721
GPM6B_HUMAN	1	3.57225	3.57225
KLH31_DANRE	1.0785	3.85125	3.57093185
HSP70_PLEWA	11.1625	39.7995	3.565464726
TR21479|c4_g1_i2	1	3.54475	3.54475
TR25198|c1_g1_i1	1.62325	5.7515	3.54320037
TR22602|c18_g1_i1	1.108	3.919	3.53700361
XIRP2_HUMAN	1.118	3.94575	3.529293381
LRRN1_BOVIN	1.078	3.789	3.514842301
TR9684|c2_g1_i1	1.03675	3.62825	3.499638293
TGM1_CANFA	7.1735	25.07	3.494807277
TRDN_CANFA	1.259	4.3845	3.482525814
TR29054|c5_g1_i1	1.1225	3.9035	3.477505568
TR5030|c2_g1_i1	1.287	4.47475	3.476884227
TR29959|c1_g1_i1	1.18475	4.09425	3.455792361
MPSF_CHICK	1.08575	3.748	3.451991711
TR26396|c26_g1_i1	1	3.391	3.391
LAMA3_HUMAN	6.97025	23.48475	3.369283742
ENOB_BOVIN	64.35575	215.97475	3.3559511
ABEC2_BOVIN	1.01025	3.3865	3.352140559
DXO_HUMAN	1	3.3315	3.3315
TR9684|c0_g1_i1	1.086	3.60975	3.323895028
TR21776|c16_g1_i1	10.64125	34.893	3.279032069
TR31042|c4_g1_i1	1.1515	3.77375	3.277247069
MXRA5_HUMAN	1.33775	4.37275	3.268734816
TR16522|c24_g1_i1	9.28775	30.35425	3.26820274
FKBP5_HUMAN	2.4225	7.90675	3.263880289
TR5659|c20_g1_i1	1.06425	3.47175	3.262156448
LORF2_HUMAN	1.44525	4.6835	3.24061581
TR25410|c0_g1_i1	4.73175	15.32975	3.239763301
TR6481|c2_g1_i1	4.25925	13.79575	3.239009215
TR27931|c2_g1_i1	1.07225	3.47275	3.238750291
TR26352|c8_g1_i1	1.1515	3.72125	3.231654364
TM182_DANRE	1.128	3.6405	3.227393617
RYR3_HUMAN	1.2075	3.89525	3.225879917
LAMB3_HUMAN	21.29225	68.565	3.220185748
LECT1_BOVIN	1.076	3.43425	3.191682156
TR20819|c22_g1_i1	1.08575	3.463	3.189500345
TR20819|c26_g1_i1	1.103	3.5125	3.184496827
TR1315|c1_g1_i1	1	3.1835	3.1835
TR22581|c4_g1_i1	1.8205	5.79125	3.181131557
TR27931|c3_g1_i1	1.19575	3.80225	3.179803471
ZN185_MOUSE	6.40325	20.3595	3.179557256
YIPF7_BOVIN	1.13475	3.606	3.177792465
TR29201|c1_g1_i1	1.15775	3.66325	3.164111423

PGAP3_HUMAN	1	3.16375	3.16375
TXLNB_MOUSE	1.14225	3.59925	3.151017728
HEMO_PONAB	2.67575	8.42925	3.150238251
TR17589|c0_g1_i1	3.172	9.94475	3.135167087
UN45B_XENLA	1.20675	3.7695	3.123679304
NUD22_BOVIN	2.862	8.90975	3.113120196
TR6481|c0_g1_i1	4.83525	15.02725	3.107853782
XIRP2_RAT	1.08825	3.3715	3.098093269
TR3033|c21_g1_i1	1.1005	3.3975	3.087233076
TR29689|c19_g1_i1	1	3.058	3.058
TR16529|c15_g1_i1	1.1055	3.38	3.057440072
TR25821|c2_g1_i1	1.022	3.12275	3.055528376
ANXA6_HUMAN	5.17825	15.71725	3.035243567
PACN3_HUMAN	5.58675	16.923	3.029131427
TR16116|c1_g1_i1	1	3.026	3.026
TR15930|c0_g1_i1	1.07375	3.24375	3.020954598
CALM_MYXGL	76.051	228.71625	3.007406214
TR25585|c1_g1_i1	1	2.9925	2.9925
PGCA_BOVIN	1.024	3.06325	2.991455078
TR26338|c7_g1_i1	1.28825	3.85225	2.990296914
TR9684|c4_g1_i1	1.081	3.23175	2.989592969
TR25198|c1_g2_i1	1.36825	4.08675	2.986844509
TR28797|c3_g1_i1	1.054	3.14225	2.98126186
TR17724|c1_g1_i1	1.05375	3.1415	2.981257414
MPSF_CHICK	1.027	3.05425	2.973953262
TR28084|c2_g1_i1	1	2.9675	2.9675
OBSCN_MOUSE	1.1155	3.304	2.961900493
RYR1_MOUSE	1.08825	3.2115	2.951068229
KCC2A_RAT	1.09375	3.2165	2.9408
SMTL1_MOUSE	1.5865	4.663	2.939174283
PDK2_MOUSE	4.01175	11.77275	2.934567209
TR10851|c2_g1_i1	1	2.93125	2.93125
TR25821|c1_g1_i1	1.015	2.96625	2.922413793
MYH7_PAPHA	1	2.92225	2.92225
TR645|c2_g1_i1	1	2.92125	2.92125
RYR1_RABIT	1.12875	3.29475	2.918936877
CIRBP_PONAB	17.2725	50.215	2.907222463
TR25842|c2_g1_i1	1.03925	3.0045	2.891027183
KEAP1_PIG	13.40225	38.529	2.874815796
TR25842|c3_g1_i1	1.04425	3.00075	2.873593488
TR5659|c22_g1_i1	2.43275	6.98925	2.872983249
TR17724|c3_g1_i1	1.0785	3.096	2.870653686
TR23021|c17_g1_i1	1.5975	4.58525	2.870266041
TR21782|c14_g1_i1	1.17675	3.377	2.86976843
IP6K3_HUMAN	1.03925	2.9805	2.867933606
TITIN_HUMAN	1.06625	3.05325	2.863540445
TR22581|c5_g1_i2	1.50675	4.3125	2.862120458

TR15118|c10_g1_i1	2.53475	7.24325	2.857579643
TR17724|c2_g1_i1	1.07375	3.0625	2.852153667
TR25266|c4_g1_i1	1.04425	2.97225	2.846301173
TITIN_MOUSE	1.06375	3.0235	2.842303173
TR13692|c0_g1_i1	154.1545	438.0665	2.841736699
MYOM3_MOUSE	2.598	7.38025	2.840742879
ACHD_CHICK	1.1495	3.257	2.833405829
DPYS_HUMAN	1.11025	3.14225	2.830218419
TR1315|c2_g1_i1	1.03675	2.934	2.829997589
TR24098|c2_g1_i1	1.03675	2.92775	2.823969134
NEST_HUMAN	6.3765	17.9765	2.819179801
TR4957|c7_g1_i1	1.103	3.10275	2.813009973
FOSB_MOUSE	1.2805	3.59725	2.809254198
MYOM3_HUMAN	1.066	2.99425	2.808864916
TR7273|c0_g1_i1	1.2875	3.61575	2.808349515
PACN3_HUMAN	2.0755	5.82475	2.806432185
IL8_CHICK	2.0475	5.74075	2.803785104
ARP21_HUMAN	1.20625	3.3755	2.798341969
TR4376|c0_g1_i1	1.37775	3.853	2.796588641
TR21772|c10_g2_i1	2.70375	7.5335	2.786315303
AT1A2_BOVIN	1.2165	3.389	2.785861077
TR4957|c6_g1_i1	1.0735	2.981	2.776897997
XIRP2_HUMAN	1.03425	2.87125	2.776166304
TR6410|c7_g1_i1	1	2.77475	2.77475
CJ071_HUMAN	1.172	3.25125	2.774104096
TR28114|c2_g1_i1	1.1255	3.11225	2.76521546
GUAD_HUMAN	1	2.761	2.761
TR29201|c2_g1_i1	1.1005	3.03575	2.758518855
TR9245|c0_g2_i1	1.823	5.0185	2.752879868
TR18582|c1_g1_i1	1.1165	3.0725	2.751903269
MYOM3_MOUSE	1.027	2.82575	2.751460565
IBP5_BOVIN	6.99125	19.18175	2.743679599
TR18539|c1_g1_i1	1.044	2.863	2.742337165
TR11059|c1_g1_i2	1.41725	3.88225	2.739283824
ADIPO_MOUSE	1.16025	3.17675	2.737987503
TR25519|c0_g1_i1	1.0465	2.86025	2.733158146
TR22563|c0_g1_i1	1.411	3.8455	2.725372077
TR1107|c0_g1_i1	1.0195	2.76875	2.715792055
TR28797|c1_g1_i1	1.044	2.822	2.703065134
TR21538|c14_g1_i1	1.3955	3.76275	2.696345396
TR9698|c0_g1_i1	1.2025	3.23625	2.691268191
TR29201|c0_g1_i1	1.0565	2.841	2.689067676
TITIN_HUMAN	1.071	2.8765	2.685807656
TR20819|c10_g1_i1	1.1005	2.95175	2.682189914
PCOC2_HUMAN	4.3305	11.6075	2.68040642
TR22563|c0_g2_i1	1	2.67675	2.67675
IOD3_XENLA	8.31975	22.2625	2.675861654

TR4657|c2_g1_i1	1.1785	3.15275	2.675222741
TITIN_MOUSE	1	2.66975	2.66975
TR9519|c9_g1_i1	1	2.66725	2.66725
ACHA_CHICK	1.333	3.5515	2.664291073
PTPRV_RAT	1	2.6585	2.6585
RYR3_RABIT	1.11225	2.94125	2.644414475
TR5061|c5_g1_i1	1.1155	2.948	2.642761094
DUS27_MOUSE	1.06375	2.80775	2.639482961
TR21538|c32_g1_i1	1.5965	4.21325	2.639054181
DUS5_HUMAN	8.66225	22.85425	2.638373402
TR21814|c25_g1_i1	1.06	2.7955	2.637264151
TR3032|c7_g1_i1	1	2.63725	2.63725
TR5735|c10_g1_i1	2.93675	7.7365	2.634374734
TR25263|c31_g1_i1	1.0835	2.84675	2.627365021
TITIN_MOUSE	1.025	2.6925	2.626829268
TR14056|c4_g1_i1	1.055	2.76675	2.622511848
ARHL1_MOUSE	1.03675	2.7165	2.620207379
TITIN_HUMAN	1.09825	2.8765	2.619166856
TR11764|c6_g1_i1	1.0875	2.8455	2.616551724
TR4259|c3_g1_i1	1	2.61575	2.61575
TR26671|c0_g1_i1	1.29125	3.3775	2.615682478
TR19944|c3_g1_i1	2.43775	6.371	2.613475541
TR4291|c1_g1_i1	1.05875	2.7565	2.603541913
TR4195|c0_g1_i1	1.33675	3.47675	2.6008977
POPD1_CHICK	2.10125	5.4645	2.600594884
TR16445|c0_g1_i1	4.1055	10.66275	2.597186701
TR15142|c9_g2_i1	1	2.5965	2.5965
TR1779|c0_g1_i1	1.118	2.901	2.594812165
TR16631|c2_g1_i1	3.30675	8.57	2.591668557
KLH31_DANRE	1.1425	2.96025	2.591028446
CHRD1_PIG	1.1975	3.10125	2.589770355
XIRP2_HUMAN	1.0245	2.65225	2.588823816
PTG3L_MOUSE	1.1665	3.012	2.582083155
TR5571|c7_g2_i1	5.039	12.99125	2.578140504
TR18727|c4_g1_i1	1.261	3.251	2.578112609
VGLL3_HUMAN	1.34475	3.466	2.577430749
LECT1_BOVIN	1.0375	2.673	2.576385542
TR7504|c0_g2_i1	1	2.574	2.574
TR5493|c2_g1_i1	1.2885	3.3145	2.572370974
OBSCN_HUMAN	1.03175	2.6515	2.5699055
TR16811|c0_g1_i1	2.366	6.06525	2.563503804
TITIN_MOUSE	1.12975	2.8945	2.562071255
TITIN_MOUSE	1.0785	2.759	2.558182661
TR28755|c1_g1_i1	1.044	2.6695	2.556992337
TR21782|c18_g1_i1	1.553	3.969	2.555698648
TITIN_HUMAN	1.044	2.664	2.551724138
TR31042|c1_g1_i1	1.14125	2.9055	2.545892662

FOSL2_CHICK	4.26425	10.8485	2.544058158
SEM3A_CHICK	3.71225	9.42925	2.540036366
TR25654|c0_g1_i1	1	2.5385	2.5385
TR11764|c2_g2_i1	1	2.536	2.536
MYSS_CYPCA	1.0245	2.59475	2.532698878
TR22643|c2_g3_i1	4.87525	12.34075	2.531306087
S22AH_RAT	1	2.528	2.528
UBP13_HUMAN	1.085	2.7405	2.525806452
TR5493|c0_g1_i1	1.125	2.841	2.525333333
AKAP6_HUMAN	1.022	2.58	2.52446184
SDF1_XENLA	2.003	5.05575	2.524088867
XIRP2_HUMAN	1.0985	2.7665	2.518434228
RYR3_HUMAN	1.087	2.737	2.517939282
PO21_NASVI	1	2.5145	2.5145
TR21044|c0_g1_i1	1.68975	4.24725	2.513537506
TR4259|c0_g1_i1	1.018	2.555	2.509823183
TR16151|c0_g2_i1	1.1055	2.771	2.506558118
TR9684|c3_g1_i1	1.06375	2.6655	2.505757932
RYR1_MOUSE	1.18425	2.96525	2.503905425
HSPB2_RAT	1.0375	2.59775	2.503855422
MYOME_HUMAN	2.52225	6.315	2.503716919
TITIN_HUMAN	1.08625	2.716	2.500345224
PGCA_HUMAN	1	2.49925	2.49925
TR24098|c27_g1_i1	1.1035	2.75675	2.498187585
CALM_XENLA	15.75375	39.25775	2.491962231
TR17970|c2_g1_i1	1	2.48725	2.48725
TR16632|c2_g1_i1	1.682	4.1805	2.485434007
TR4333|c10_g1_i1	1.217	3.02425	2.485004108
TR5320|c1_g1_i1	1.044	2.58875	2.479645594
TR19659|c0_g1_i1	1.475	3.651	2.475254237
MY18B_HUMAN	1.042	2.57775	2.473848369
MXRA5_HUMAN	1.0735	2.65525	2.473451327
TR24297|c1_g1_i1	1.1085	2.738	2.470004511
TITIN_HUMAN	1.15475	2.8495	2.467633687
AKAP6_HUMAN	1.022	2.51775	2.463551859
TR28794|c3_g1_i1	1	2.463	2.463
TR18694|c9_g1_i1	1.25225	3.0805	2.45997205
SYNP2_HUMAN	1.327	3.26225	2.458364732
TSP4_XENLA	1.61675	3.972	2.456780578
PUA1A_XENLA	1.6295	3.99975	2.454587297
BAG3_HUMAN	6.21175	15.2225	2.450597658
TR26390|c12_g1_i1	1	2.4355	2.4355
TR23021|c10_g1_i1	1.441	3.503	2.430950729
AZIN2_XENLA	1.1155	2.7075	2.427162707
TR26388|c25_g1_i1	1.0685	2.59225	2.426064577
TAL2_TACTR	1.308	3.165	2.419724771
TR26721|c2_g1_i1	1.081	2.6155	2.419518964

TR12205|c1_g1_i1	1	2.4165	2.4165
TR24745|c1_g1_i1	1.12875	2.72575	2.414839424
TR7846|c0_g1_i1	1	2.41275	2.41275
TR24093|c1_g1_i1	1.09325	2.6355	2.410702035
TR30482|c0_g1_i1	1.0785	2.5985	2.409364859
ACHB_XENLA	1.17425	2.82425	2.405152225
TR30036|c6_g1_i1	1.2755	3.06325	2.401607213
TR18814|c2_g1_i1	1.20075	2.883	2.400999375
TR16151|c3_g1_i1	1.13775	2.72925	2.398813448
TR26390|c4_g1_i1	1.1275	2.69975	2.394456763
CCG6_RAT	1.054	2.52275	2.393500949
TR5735|c11_g1_i1	1.97625	4.727	2.391903858
ACHD_XENLA	1.025	2.44825	2.388536585
UACA_HUMAN	2.95175	7.041	2.385364614
TR10651|c1_g2_i1	2.16475	5.154	2.38087539
TR20035|c6_g1_i1	2.1405	5.0905	2.378182668
TR10851|c1_g1_i1	1.0465	2.48675	2.376254181
FGFR4_PLEWA	1.12475	2.66475	2.369193154
OBSCN_HUMAN	1.0955	2.58825	2.362619808
TR19037|c0_g1_i1	1.3825	3.2655	2.362025316
AKAP6_HUMAN	1	2.36175	2.36175
AKAP6_HUMAN	1.0295	2.43	2.360369111
TR26388|c21_g1_i1	1.17025	2.7605	2.358897671
TR18727|c29_g1_i1	1.588	3.74525	2.358469773
CLC3A_XENLA	1	2.354	2.354
TR23021|c5_g2_i1	1.62325	3.8155	2.350531341
TR19100|c1_g1_i1	1.0195	2.39525	2.349435998
MSS51_HUMAN	1.298	3.0475	2.347842835
TR28797|c5_g1_i1	1.06625	2.50125	2.345838218
MXRA5_HUMAN	1.174	2.754	2.345826235
TR19100|c2_g1_i1	1	2.34425	2.34425
TR27931|c5_g1_i1	1.119	2.61875	2.34025916
OBSCN_HUMAN	1.069	2.49675	2.335594013
CRIP2_RAT	1.8485	4.316	2.334866108
TR9910|c1_g1_i1	1	2.334	2.334
AKAP6_HUMAN	1	2.332	2.332
TR27931|c0_g1_i1	1.10175	2.56925	2.331971863
TR11572|c0_g1_i1	1.1155	2.601	2.331689825
MY18B_HUMAN	1	2.33075	2.33075
TPPP3_XENLA	14.64875	34.099	2.327775407
TRDN_RAT	1	2.327	2.327
TR8295|c0_g1_i1	1.16875	2.716	2.323850267
TR28051|c1_g1_i1	1	2.323	2.323
TR12590|c1_g1_i1	1.1875	2.75625	2.321052632
OBSCN_MOUSE	1.044	2.421	2.318965517
SEM3B_HUMAN	3.3295	7.70225	2.313335336
ABEC2_BOVIN	1.2655	2.927	2.312919795

GLSL_MOUSE	1.0565	2.44175	2.311168954
TR12391|c16_g1_i1	1.5945	3.681	2.308560677
TR5634|c5_g1_i1	1.7095	3.9325	2.300380228
DKK1_HUMAN	1.89375	4.35575	2.300066007
TR4957|c4_g1_i1	1.0515	2.4155	2.297194484
TR25254|c3_g1_i1	1	2.2965	2.2965
TR27723|c0_g1_i1	1.11525	2.561	2.296346111
TR5822|c0_g1_i1	1.32725	3.039	2.289696741
FBN1_BOVIN	5.4	12.348	2.286666667
ZN185_MOUSE	9.093	20.7765	2.284889475
TR20003|c8_g1_i1	1.1325	2.587	2.284326711
ABC3G_MACMU	1	2.2805	2.2805
ASPN_BOVIN	1.08975	2.484	2.279421886
TITIN_MOUSE	1.12	2.5525	2.279017857
RABP1_CHICK	1.13025	2.56675	2.270957753
TR18587|c1_g1_i1	1.23525	2.7995	2.266342846
TR8594|c0_g1_i1	1	2.26575	2.26575
OBSCN_MOUSE	1.03675	2.3485	2.265251989
TR20542|c0_g1_i1	1.049	2.376	2.265014299
ABLM2_MOUSE	1	2.26475	2.26475
MY18B_HUMAN	1	2.2635	2.2635
ALKB8_BOVIN	1	2.2635	2.2635
SIA7B_MOUSE	3.0755	6.94675	2.258738417
LDB3_MOUSE	1.09825	2.4785	2.256772137
PERI_XENLA	1	2.246	2.246
DUS14_MOUSE	1.1295	2.536	2.245241257
TR21646|c4_g1_i1	2.09125	4.68325	2.23945009
TR14056|c5_g1_i1	1.27875	2.8565	2.233822092
HIF3A_HUMAN	7.87	17.49875	2.223475222
HGFL_BOVIN	6.062	13.4665	2.221461564
TR13778|c0_g1_i1	1.03425	2.29675	2.220691322
WNT5A_RABIT	1.96675	4.3565	2.215075632
TR23769|c1_g1_i1	1.044	2.31175	2.214319923
NEUL2_HUMAN	1.15475	2.5565	2.213899112
TR24421|c1_g1_i1	1.49725	3.31475	2.213892136
TR9896|c1_g1_i1	1.081	2.3925	2.213228492
FA76B_HUMAN	1	2.20975	2.20975
TITIN_MOUSE	1.0245	2.2595	2.205466081
ANPRB_ANGJA	1	2.1985	2.1985
PAX1_MOUSE	2.13875	4.69625	2.195791935
ALPK2_HUMAN	1.0295	2.26	2.195240408
TR18443|c1_g1_i1	1.24575	2.726	2.188240016
TR28051|c0_g1_i1	1.01225	2.21425	2.187453692
TR25842|c1_g1_i1	1	2.18675	2.18675
YLAT2_XENTR	1.061	2.31825	2.184967012
AKAP6_HUMAN	1.03425	2.256	2.18129079
TR21780|c2_g1_i1	1	2.18125	2.18125

TR15477|c1_g1_i1	1.19125	2.5965	2.179643232
TMOD1_BOVIN	1.1025	2.4025	2.179138322
TOR3A_HUMAN	1.88525	4.10775	2.178888742
TITIN_HUMAN	1.0245	2.23075	2.177403612
TRI55_HUMAN	1.09975	2.39425	2.177085701
TR15492|c2_g1_i1	1	2.176	2.176
TR27786|c0_g1_i1	1.39	3.0235	2.175179856
RHG24_HUMAN	2.88925	6.283	2.174612789
CNN2_MOUSE	74.28375	161.09675	2.168667441
UBP13_HUMAN	1.06375	2.3065	2.16827262
TR18745|c10_g1_i2	1	2.16575	2.16575
TR21782|c9_g1_i1	1.19475	2.58425	2.163004813
TR5634|c6_g1_i1	1.313	2.839	2.162223915
LAMA2_MOUSE	1.54525	3.33925	2.160977188
TR30753|c0_g2_i1	1.2135	2.622	2.160692213
PINLY_HUMAN	1.936	4.18075	2.159478306
TR6826|c4_g1_i1	1.07075	2.3115	2.158767219
RYR3_RABIT	1.09375	2.36	2.157714286
STEA4_HUMAN	1.64375	3.5325	2.14904943
SHC4_HUMAN	1.342	2.884	2.149031297
TR30181|c0_g1_i1	1	2.1455	2.1455
P3H2_HUMAN	1.91	4.09775	2.145418848
RYR3_MOUSE	1.1395	2.4425	2.143483984
TR21776|c19_g1_i1	1.28275	2.7465	2.141103099
TR22825|c0_g1_i1	1.059	2.2625	2.136449481
RUSC2_HUMAN	1.31125	2.795	2.131553861
PGCA_PIG	1	2.1285	2.1285
YLAT2_XENTR	1.05475	2.242	2.125622185
TR23769|c0_g1_i1	1.1085	2.3525	2.122237258
CA2D1_MOUSE	1.071	2.272	2.121381886
MYLK2_BOVIN	1.1025	2.329	2.112471655
TR26674|c1_g1_i1	1.03175	2.177	2.110007269
TSN7_HUMAN	2.4725	5.2085	2.106572295
S38A3_MOUSE	1.11275	2.3415	2.104246237
MYCD_PIG	1.069	2.24825	2.10313377
TR6453|c11_g1_i1	1.07175	2.25275	2.101936086
TR20890|c1_g1_i1	1.213	2.5495	2.101813685
SEM3C_CHICK	1.449	3.04475	2.101276743
TR16129|c0_g1_i1	1.54875	3.2495	2.098143664
TENS_CHICK	2.67225	5.59175	2.092525026
MYLK2_RABIT	1.16575	2.435	2.088784045
TR8625|c0_g1_i1	1	2.0805	2.0805
TR20035|c1_g1_i1	1.63575	3.39075	2.072902338
DREB_HUMAN	2.05025	4.24575	2.070845019
OBSCN_MOUSE	1.03925	2.14925	2.068077941
TR23064|c2_g1_i1	1.064	2.19475	2.062734962
MY18B_HUMAN	1.079	2.22375	2.060936052

TR2981|c10_g1_i1	1.0565	2.1685	2.052531945
RYR3_MOUSE	1.076	2.20825	2.052276952
TR21646|c3_g1_i1	1.5565	3.19275	2.051236749
TR24821|c0_g1_i1	1.044	2.141	2.050766284
TR28722|c0_g1_i1	1.1345	2.3235	2.048038784
RUSC2_HUMAN	1.1845	2.42225	2.044955678
TR8074|c0_g1_i1	1.069	2.1825	2.041627689
BIG1_HUMAN	2.353	4.795	2.037824054
RBM43_HUMAN	1	2.0375	2.0375
TR5493|c1_g1_i1	1.2575	2.5595	2.035387674
PTGIS_MOUSE	1.86375	3.7825	2.029510396
FLIP1_HUMAN	1.077	2.177	2.021355617
LORF2_HUMAN	1	2.01925	2.01925
SPTB2_MOUSE	1.71875	3.46975	2.018763636
GPC5B_HUMAN	1.48975	2.9995	2.013425071
SCN1B_CANFA	1.044	2.0965	2.008141762
CAN5_MOUSE	1.295	2.59875	2.006756757
FA32A_PONAB	1	2.00425	2.00425
TR12590|c0_g1_i1	1.34525	2.689	1.998884966
TR25527|c0_g1_i1	1.153	2.3025	1.996964441
TR22796|c1_g1_i1	1.08575	2.1665	1.995394888
SEM3C_PONAB	1.44425	2.8765	1.991691189
PE2R3_HUMAN	1.75625	3.4975	1.991459075
FLIP1_MOUSE	1.08375	2.149	1.982929642
SIX1B_DANRE	1	1.9815	1.9815
TRDN_RAT	1	1.97925	1.97925
SRBS1_MOUSE	1.74175	3.43475	1.972010909
CI072_HUMAN	1	1.95425	1.95425
TR119|c1_g1_i1	1.022	1.992	1.949119374
TR12391|c20_g1_i1	1.28725	2.508	1.948339483
HS3SA_HUMAN	1.1925	2.323	1.948008386
TR5675|c6_g2_i1	1	1.9475	1.9475
RTJK_DROFU	1.3565	2.6395	1.945816439
TRDN_RAT	1	1.94475	1.94475
TR7446|c1_g1_i1	1	1.94425	1.94425
TR19736|c1_g1_i1	1.30175	2.52825	1.942193201
RYR3_HUMAN	1.15225	2.2145	1.921891951
CR021_HUMAN	1.09475	2.102	1.920073076
TRDN_RAT	1	1.92	1.92
TR13892|c0_g2_i1	1.015	1.94475	1.916009852
DYST_HUMAN	2.39725	4.591	1.915111065
BORG1_RAT	1.4945	2.84825	1.905821345
TR20003|c3_g1_i1	1.1335	2.15175	1.898323776
NEBU_HUMAN	1.005	1.90075	1.891293532
C2CD3_XENTR	1	1.89075	1.89075
MY18B_HUMAN	1.081	2.03975	1.886910268
TR5634|c26_g1_i1	1	1.8855	1.8855

TR17498|c1_g1_i3	1	1.882	1.882
TR9886|c0_g1_i1	1.35175	2.54275	1.881080081
MEOX1_PONPY	1.2025	2.25325	1.873804574
TR5641|c8_g2_i1	1.6795	3.13775	1.868264364
TR11082|c22_g1_i1	1.27875	2.38775	1.867253177
ATS8_HUMAN	1.477	2.74725	1.860020311
TR23171|c0_g1_i1	1.2245	2.27075	1.85443038
CILP1_PIG	1.2715	2.3545	1.851749902
KBTB2_PONAB	1	1.8445	1.8445
BCL6_CHICK	1.548	2.84775	1.839631783
TR17998|c1_g1_i1	1.45375	2.6725	1.838349097
TR18716|c2_g2_i1	1	1.838	1.838
TR23084|c0_g1_i1	1.26975	2.327	1.832644221
TR20035|c4_g1_i1	1.279	2.337	1.827208757
TR17753|c6_g1_i5	1	1.819	1.819
WNT9A_CHICK	1.5175	2.7515	1.813179572
MET25_HUMAN	1	1.813	1.813
EPHA3_HUMAN	1.1385	2.044	1.795344752
S6A14_HUMAN	1	1.7825	1.7825
SPTN5_HUMAN	1.5945	2.83875	1.780338664
RYR1_MOUSE	1	1.7795	1.7795
TR19244|c1_g2_i1	1.0805	1.918	1.775104118
ARHGP_HUMAN	1.8745	3.3085	1.765004001
CONA1_HUMAN	1	1.7535	1.7535
PPR3A_RABIT	1	1.75275	1.75275
TR21463|c3_g3_i1	1	1.7515	1.7515
RUN3A_HUMAN	1.1115	1.94375	1.748762933
TR29089|c0_g1_i1	1	1.734	1.734
G2E3_CHICK	1	1.7335	1.7335
TR4474|c2_g1_i1	1.392	2.406	1.728448276
LAMB1_HUMAN	1.4255	2.461	1.726411785
TR11103|c16_g1_i1	1.2615	2.17575	1.724732461
IL6RB_HUMAN	1	1.714	1.714
PKHS1_MOUSE	1	1.70875	1.70875
NTNG1_HUMAN	1.269	2.16225	1.703900709
TR26688|c0_g1_i1	1.2505	2.124	1.698520592
GLPC_HUMAN	1	1.69325	1.69325
GPC5_HUMAN	1.1805	1.94075	1.644006777
MYSS_CYPCA	1.00975	1.6595	1.643476108
TR22868|c1_g1_i1	1.293	2.112	1.633410673
TRDN_RAT	1.0395	1.68375	1.61976912
TR29829|c1_g1_i1	1	1.59175	1.59175
TR18902|c0_g1_i1	1.015	1.60925	1.58546798
TR11714|c3_g1_i1	1	1.579	1.579
TR15158|c6_g3_i1	1	1.57125	1.57125
MAP4_MOUSE	1.156	1.80875	1.56466263
TR12437|c0_g1_i1	1	1.55175	1.55175

TR16463|c1_g5_i1	1	1.545	1.545
TR26568|c3_g2_i1	1	1.54425	1.54425
NEXN_HUMAN	1.5085	2.32675	1.542426251
TR20867|c0_g3_i1	1.0675	1.6435	1.539578454
OCTC_HUMAN	1	1.5385	1.5385
E41L2_HUMAN	1	1.5215	1.5215
KI26B_HUMAN	1	1.514	1.514
TR19795|c1_g1_i1	1	1.50425	1.50425
TR16609|c4_g1_i1	1	1.4765	1.4765
TR9614|c1_g2_i1	1	1.45375	1.45375
QORX_HUMAN	1	1.4355	1.4355
TR11115|c2_g1_i1	1	1.42675	1.42675
TR3020|c2_g1_i1	1	1.3965	1.3965
TR15182|c4_g1_i7	1	1.39575	1.39575
TR3002|c3_g2_i1	1	1.339	1.339
GALT6_HUMAN	1	1.338	1.338
F214B_BOVIN	1	1.32825	1.32825
TP4AP_HUMAN	1	1.2985	1.2985
TR10063|c5_g2_i1	1.00975	1.2765	1.264174301
C1GLC_HUMAN	1	1.08075	1.08075
STML1_HUMAN	1.114	1	0.897666068
TR21471|c0_g1_i4	1.261	1	0.793021412
PCF11_HUMAN	1.3675	1	0.731261426
GNA12_HUMAN	1.5075	1.00725	0.668159204
NGAP_HUMAN	1.5925	1.0345	0.649607535
TR5640|c1_g1_i1	1.6205	1.04425	0.644399877
TF211_SCHPO	1.557	1	0.642260758
CNRP1_MOUSE	1.58025	1	0.632811264
TR21759|c5_g1_i1	1.58075	1	0.632611102
TAOK1_HUMAN	1.626	1	0.61500615
GTR12_XENLA	1.80675	1.10725	0.612840736
TR5576|c1_g2_i1	1.64975	1	0.606152447
STAU1_HUMAN	1.65	1	0.606060606
TR19472|c0_g2_i1	1.6625	1	0.601503759
TR29693|c6_g1_i6	1.67525	1	0.596925832
TR3951|c0_g2_i1	1.6765	1	0.596480763
TR3049|c2_g2_i1	1.69725	1	0.589188393
B4GT4_HUMAN	1.7525	1	0.570613409
SEPT5_HUMAN	1.764	1	0.566893424
LORF2_HUMAN	1.91975	1.0535	0.548769371
TR22454|c4_g1_i1	1.89	1.03175	0.545899471
TR1643|c0_g1_i1	2.77975	1.50175	0.540246425
TR4094|c3_g1_i1	2.10225	1.127	0.536092282
TR180|c4_g1_i1	2.5035	1.3155	0.52546435
TR17352|c2_g1_i1	2.113	1.10775	0.524254614
TR180|c8_g1_i1	2.39175	1.23025	0.514372322
YRD6_CAEEL	1.99975	1.027	0.513564196

TR26590|c7_g1_i1	3.03225	1.54775	0.510429549
TR7506|c0_g1_i1	2.071	1.05425	0.509053597
TR5551|c9_g1_i1	1.9845	1	0.503905266
PSIP1_FELCA	1.9895	1	0.502638854
TR23244|c0_g1_i1	2.48225	1.2425	0.500553933
TR8241|c1_g2_i2	2.46775	1.23025	0.498531051
TR4752|c2_g1_i1	2.0065	1	0.498380264
TR16528|c24_g1_i1	2.49125	1.22975	0.493627697
TR12812|c2_g1_i1	2.03375	1	0.49170252
GRP75_CHICK	2.047	1	0.488519785
PKHS1_MOUSE	2.06075	1.00525	0.487807837
TR15112|c1_g2_i1	2.178	1.06125	0.487258953
TR15139|c15_g1_i1	3.94575	1.901	0.481784198
DHR11_CHICK	3.2885	1.5815	0.480918352
TR23918|c0_g1_i1	2.233	1.0715	0.479847738
NETO2_RAT	2.4955	1.18775	0.475956722
TR9219|c0_g1_i1	2.2665	1.0775	0.475402603
TR5551|c3_g3_i1	2.10925	1	0.474102169
TR18204|c0_g1_i1	2.11125	1	0.473653049
TR180|c2_g1_i1	2.77925	1.31475	0.473059279
TR19991|c1_g1_i1	2.1145	1	0.472925041
TR27454|c0_g1_i1	2.273	1.056	0.46458425
CP2G1_RABIT	2.15625	1	0.463768116
WNT3_MOUSE	2.26875	1.044	0.460165289
TR12182|c3_g1_i1	2.617	1.17825	0.45022927
TR5577|c5_g1_i2	2.22275	1	0.44989315
TR5287|c0_g1_i1	2.5995	1.165	0.448163108
TR12182|c0_g1_i1	2.632	1.17675	0.447093465
TR15200|c18_g4_i1	3.19075	1.4265	0.447073572
TR16442|c3_g1_i1	2.238	1	0.446827525
TR19244|c1_g1_i1	2.24025	1	0.446378752
TR11564|c0_g1_i1	2.24925	1	0.444592642
MBL2_MOUSE	2.36025	1.04175	0.441372736
TR18897|c1_g1_i1	2.26725	1	0.441062962
S29A3_BOVIN	2.3025	1	0.434310532
S6A14_HUMAN	2.69425	1.16725	0.43323745
TR6443|c6_g1_i1	2.33725	1	0.427853246
F199X_XENTR	2.3385	1	0.427624546
TR9312|c0_g1_i1	2.34325	1	0.426757708
TR29005|c26_g1_i1	2.815	1.1965	0.425044405
TR30099|c0_g3_i1	2.35425	1	0.424763725
TR9989|c4_g2_i1	2.53575	1.0685	0.421374347
PIGW_BOVIN	2.554	1.0715	0.41953798
MAST2_HUMAN	2.39875	1	0.416883794
TR5675|c19_g1_i1	2.60325	1.08275	0.415922405
UDB17_RAT	3.046	1.24525	0.408814839
LFG1_HUMAN	11.424	4.669	0.40870098

TR15532|c2_g1_i1	2.6885	1.07525	0.399944207
TR16600|c13_g1_i1	3.74775	1.47575	0.393769595
TR18673|c5_g1_i1	2.7225	1.071	0.39338843
DHRS4_RABIT	2.651	1.0325	0.38947567
TR5675|c5_g1_i1	2.5935	1	0.385579333
TR6443|c13_g1_i1	2.655	1.02325	0.385404896
TR29078|c0_g1_i1	2.8055	1.08025	0.385047229
TR21503|c5_g2_i1	2.63675	1	0.379254764
RND1_HUMAN	3.49175	1.32425	0.379251092
RGPS2_HUMAN	2.64725	1	0.377750496
TR26072|c0_g1_i1	2.6675	1	0.374882849
TR20142|c1_g1_i1	2.793	1.044	0.373791622
FUCL4_ANGJA	2.735	1.02225	0.373765996
TR29324|c1_g1_i1	3.03725	1.13375	0.373281752
EGLN3_MOUSE	2.69475	1	0.371091938
TR20142|c0_g1_i1	3.16975	1.154	0.364066567
LORF1_MOUSE	2.7535	1	0.363174142
LAAT1_RAT	2.772	1	0.360750361
EYA2_HUMAN	6.146	2.217	0.360722421
LPCT4_XENTR	2.78825	1	0.358647897
LORF2_HUMAN	2.9855	1.06775	0.357645286
CAN7_MOUSE	2.8635	1	0.349222979
TR20866|c7_g1_i1	2.8635	1	0.349222979
MUC3A_HUMAN	2.88225	1	0.346951167
TR23909|c0_g1_i1	3.922	1.34475	0.342873534
TR23085|c1_g2_i1	2.9205	1	0.342407122
MYO5B_HUMAN	2.94725	1	0.339299347
TR20346|c0_g1_i1	3.553	1.19275	0.335702223
TR21763|c41_g1_i1	2.9815	1	0.335401643
LIN1_NYCCO	3.05075	1.01475	0.332623125
TR5547|c5_g1_i1	3.03125	1	0.329896907
GNA1_PONAB	22.2535	7.20275	0.323668187
PIGR_BOVIN	4.99125	1.59825	0.320210368
DOCK1_HUMAN	3.13675	1	0.318801307
TR21763|c17_g1_i1	3.14175	1	0.318293944
TR9857|c1_g1_i1	3.20975	1.01975	0.317703871
KV403_HUMAN	14.86875	4.675	0.314417823
TR18703|c4_g1_i1	24.52075	7.668	0.312714742
LITAF_HUMAN	5.269	1.6335	0.310020877
TR16714|c2_g1_i1	3.638	1.10925	0.304906542
TR16442|c20_g1_i1	3.31325	1	0.301818456
IGHM_SUNMU	382.52675	113.83525	0.297587685
TR15821|c0_g1_i1	3.37175	1	0.296581894
TR25885|c0_g6_i1	3.379	1	0.295945546
TR19965|c1_g1_i1	6.61325	1.8895	0.285714286
BICD2_HUMAN	3.50625	1	0.285204991
LECA_PLEWA	3.52875	1	0.283386468

RDH7_RAT	12.091	3.38975	0.280353155
TR24287|c0_g1_i1	3.577	1	0.27956388
ST2B1_RAT	3.733	1.02575	0.274778998
TR23697|c0_g1_i1	114.8035	31.54025	0.274732478
TR24294|c0_g1_i1	6.49525	1.64125	0.252684654
COR1B_MOUSE	4.0235	1	0.248539829
MUC5B_HUMAN	4.1945	1.01725	0.242519967
TR18673|c3_g1_i5	4.847	1.164	0.240148545
GRAA_HUMAN	5.006	1.202	0.240111866
ARHGG_MOUSE	4.25325	1.013	0.238170811
RN213_HUMAN	4.2135	1	0.237332384
TR16442|c16_g1_i1	4.3115	1	0.231937841
OTX5_XENTR	4.383	1	0.228154232
UBP19_MOUSE	4.39475	1	0.227544229
TR21763|c36_g1_i1	4.402	1	0.227169468
TR2977|c20_g1_i1	4.4115	1	0.226680267
CTDS2_HUMAN	4.51675	1	0.221398129
ZC3HA_MOUSE	4.6375	1	0.215633423
LV302_HUMAN	19.48725	4.197	0.215371589
MSMB_STRCA	4.99825	1	0.200070025
TR5076|c0_g1_i1	9.83725	1.93275	0.196472591
TRI17_BOVIN	5.1035	1	0.19594396
TR5675|c34_g1_i1	5.10775	1	0.195780921
VPI1_SCOJE	25.02925	4.869	0.194532397
ZC3HA_MOUSE	5.21675	1.00975	0.193559208
CQ059_BOVIN	5.29025	1	0.189026984
TR11085|c1_g1_i1	5.48	1.0195	0.186040146
TR10050|c16_g1_i1	5.459	1	0.183183733
FUCL5_ANGJA	300.59175	54.04825	0.179806166
FUCL5_ANGJA	5.5875	1	0.178970917
TR16442|c34_g1_i1	5.65825	1	0.176733089
TR21543|c6_g3_i1	8.12625	1.4015	0.172465774
TR339|c0_g1_i1	5.849	1	0.170969396
TGM3_BOVIN	5.89075	1	0.169757671
TRI39_MOUSE	15.303	2.58625	0.16900281
TR16442|c16_g2_i1	6.27625	1.00975	0.160884286
LV603_HUMAN	121.301	19.419	0.160089364
FBX36_MOUSE	6.83425	1.0935	0.160002926
RN125_MOUSE	8.615	1.3625	0.158154382
MUC2L_RAT	6.44425	1.0175	0.157892695
TR18673|c3_g1_i1	6.63325	1.03425	0.155919044
TR5581|c0_g1_i2	6.43575	1	0.155382046
ADH1_PELPE	1194.877	183.6615	0.153707453
TR18673|c3_g1_i3	7.3065	1.10525	0.151269418
HVM46_MOUSE	40.617	5.63825	0.138815028
TR18673|c3_g1_i2	9.58575	1.3065	0.136296064
PAX6_MOUSE	8.18325	1.112	0.135887331

RETN_BOVIN	7.64375	1	0.130825838
HVM46_MOUSE	89.03575	11.113	0.124815032
TR29027|c20_g1_i1	11.2915	1.28725	0.114001683
HV02_XENLA	19.59425	2.22775	0.113694068
K2C5_XENLA	370.1925	39.193	0.105871945
TGM4_HUMAN	12.23	1.206	0.098609975
MUC5B_HUMAN	10.1825	1	0.098207709
TR29027|c16_g1_i1	12.2055	1.1415	0.093523412
PINLY_HUMAN	18.023	1.638	0.090883871
TR29027|c0_g1_i1	12.913	1.1415	0.088399288
TR29916|c0_g2_i1	13.51225	1.15	0.085107958
TR17495|c15_g1_i1	27.635	2.12325	0.076831916
TR26416|c14_g1_i1	19.643	1.4815	0.07542127
TR17495|c5_g2_i1	18.36125	1.34725	0.073374634
BPIB2_HUMAN	17.50175	1.02775	0.058722699
K2C6A_MOUSE	203.88475	11.77475	0.05775199
TR29027|c11_g1_i1	22.2005	1.27875	0.057600054
TR29916|c0_g1_i1	23.603	1.3335	0.056497055
LECA_PLEWA	22.22575	1.018	0.045802729
TKTL2_MOUSE	54.728	1	0.018272182
