# Supplementary material for: Identification of regenerative roadblocks via repeat deployment of limb regeneration in axolotls
Source: NPJ Regen Med. 2017 Nov 6;2:30. doi: 10.1038/s41536-017-0034-z (PMC5677943; doi:10.1038/s41536-017-0034-z)
Supplement: Supplementary file 10 — Supplementary Table 2 [file 41536_2017_34_MOESM10_ESM.pdf]

Gene Ontology: Biological processes downregulated in repeated amputation vs. single amputation			
GO-ID	p-value	corrected p-value	Description
6958	4.19E-05	1.88E-02	complement activation, classical pathway
2455	5.52E-05	1.88E-02	humoral immune response mediated by circulating immunoglobulin
10185	6.41E-05	1.88E-02	regulation of cellular defense response
32197	7.50E-05	1.88E-02	transposition, RNA-mediated
6956	1.14E-04	1.88E-02	complement activation
2541	1.22E-04	1.88E-02	activation of plasma proteins involved in acute inflammatory response
1868	1.28E-04	1.88E-02	regulation of complement activation, lectin pathway
32196	2.33E-04	2.41E-02	transposition
19724	2.34E-04	2.41E-02	B cell mediated immunity
16064	2.34E-04	2.41E-02	immunoglobulin mediated immune response
19538	3.42E-04	3.19E-02	protein metabolic process
6959	4.71E-04	4.04E-02	humoral immune response
2449	5.97E-04	4.72E-02	lymphocyte mediated immunity
6410	7.56E-04	4.99E-02	transcription, RNA-dependent
32199	7.56E-04	4.99E-02	transcription involved in RNA-mediated transposition
51605	7.77E-04	4.99E-02	protein maturation by peptide bond cleavage

Gene Ontology: Biological processes upregulated in repeated amputation vs. single amputation			
GO-ID	p-value	corrected p-value	Description
6936	9.5114E-31	2.1094E-27	muscle contraction
3012	1.8577E-30	2.1094E-27	muscle system process
6941	8.9734E-24	6.7928E-21	striated muscle contraction
61061	1.318E-20	7.4828E-18	muscle structure development
7517	8.6381E-18	3.9234E-15	muscle organ development
55001	3.417E-17	1.2933E-14	muscle cell development
51146	7.3623E-17	2.3886E-14	striated muscle cell differentiation
55002	2.273E-16	6.4526E-14	striated muscle cell development
14706	3.3431E-15	8.4357E-13	striated muscle tissue development
30239	4.473E-15	1.0158E-12	myofibril assembly
60537	1.0744E-14	2.2182E-12	muscle tissue development
42692	1.7667E-14	3.3435E-12	muscle cell differentiation
30029	2.0042E-14	3.5011E-12	actin filament-based process
44057	2.9E-14	4.069E-12	regulation of system process
55008	3.1375E-14	4.069E-12	cardiac muscle tissue morphogenesis
90257	3.1984E-14	4.069E-12	regulation of muscle system process
30049	3.2251E-14	4.069E-12	muscle filament sliding
33275	3.2251E-14	4.069E-12	actin-myosin filament sliding
31032	3.5853E-14	4.2853E-12	actomyosin structure organization
60415	6.3335E-14	7.1917E-12	muscle tissue morphogenesis
48738	1.9135E-13	2.0693E-11	cardiac muscle tissue development
70252	1.3084E-12	1.3506E-10	actin-mediated cell contraction
45214	2.3596E-12	2.3298E-10	sarcomere organization
6937	3.6309E-12	3.4358E-10	regulation of muscle contraction
30036	4.882E-12	4.4348E-10	actin cytoskeleton organization
71415	2.6076E-11	2.1933E-09	cellular response to purine

71313	2.6076E-11	2.1933E-09	cellular response to caffeine
48747	7.5677E-11	6.1379E-09	muscle fiber development
60047	1.3959E-10	1.0931E-08	heart contraction
3015	1.9973E-10	1.5119E-08	heart process
10927	2.2659E-10	1.6599E-08	cellular component assembly involved in morphogenesis
60048	2.5222E-10	1.79E-08	cardiac muscle contraction
3008	4.3902E-10	3.0213E-08	system process
30048	5.4102E-10	3.5475E-08	actin filament-based movement
48739	5.4673E-10	3.5475E-08	cardiac muscle fiber development
31000	1.1657E-09	7.3538E-08	response to caffeine
3007	1.9978E-09	1.2262E-07	heart morphogenesis
14074	2.2944E-09	1.3712E-07	response to purine
3009	4.2336E-09	2.4036E-07	skeletal muscle contraction
7512	4.2336E-09	2.4036E-07	adult heart development
70296	8.068E-09	4.4689E-07	sarcoplasmic reticulum calcium ion transport
71312	1.9954E-08	0.000001079	cellular response to alkaloid
7507	2.2356E-08	1.1807E-06	heart development
55013	4.156E-08	2.1451E-06	cardiac muscle cell development
55006	9.7021E-08	4.8963E-06	cardiac cell development
32971	1.2087E-07	5.9673E-06	regulation of muscle filament sliding
50801	2.4464E-07	0.000011821	ion homeostasis
7010	2.7069E-07	0.000012807	cytoskeleton organization
55003	2.799E-07	0.000012972	cardiac myofibril assembly
15674	2.9468E-07	0.000013384	di-, tri-valent inorganic cation transport
8016	3.739E-07	0.000016498	regulation of heart contraction
43279	3.7776E-07	0.000016498	response to alkaloid
6816	4.1595E-07	0.000017823	calcium ion transport
50881	4.8212E-07	0.000019907	musculoskeletal movement
50879	4.8212E-07	0.000019907	multicellular organismal movement
32501	5.8976E-07	0.000023723	multicellular organismal process
48769	5.9543E-07	0.000023723	sarcomerogenesis
2026	6.2873E-07	0.000024618	regulation of the force of heart contraction
43271	9.0457E-07	0.000034818	negative regulation of ion transport
3300	9.3889E-07	0.000035537	cardiac muscle hypertrophy
6873	9.8446E-07	0.000036651	cellular ion homeostasis
55010	1.0535E-06	0.000038589	ventricular cardiac muscle tissue morphogenesis
70838	1.1384E-06	0.000041035	divalent metal ion transport
48878	1.2047E-06	0.000042747	chemical homeostasis
32970	1.2599E-06	0.000044021	regulation of actin filament-based process
55082	1.3829E-06	0.000047585	cellular chemical homeostasis
10959	1.4589E-06	0.000049449	regulation of metal ion transport
9888	1.5049E-06	0.000050259	tissue development
55066	0.000001539	0.000050652	di-, tri-valent inorganic cation homeostasis
55007	1.6115E-06	0.00005228	cardiac muscle cell differentiation
6942	0.000001687	0.000053212	regulation of striated muscle contraction
3229	0.000001687	0.000053212	ventricular cardiac muscle tissue development
14896	2.5363E-06	0.000077835	muscle hypertrophy

14897	2.5363E-06	0.000077835	striated muscle hypertrophy
8015	2.8454E-06	0.000086158	blood circulation
55080	2.9064E-06	0.000086847	cation homeostasis
3013	3.0547E-06	0.000090095	circulatory system process
30005	3.5151E-06	0.00010234	cellular di-, tri-valent inorganic cation homeostasis
14808	4.0461E-06	0.00011631	release of sequestered calcium ion into cytosol by sarcoplasmic reticulum
6874	4.2196E-06	0.00011978	cellular calcium ion homeostasis
51924	0.00000558	0.00015645	regulation of calcium ion transport
6875	6.3318E-06	0.00017536	cellular metal ion homeostasis
48856	7.7053E-06	0.00021054	anatomical structure development
30241	7.9729E-06	0.00021054	skeletal muscle myosin thick filament assembly
30240	7.9729E-06	0.00021054	skeletal muscle thin filament assembly
71688	7.9729E-06	0.00021054	striated muscle myosin thick filament assembly
43269	8.6404E-06	0.00022298	regulation of ion transport
55074	8.6404E-06	0.00022298	calcium ion homeostasis
19725	8.8815E-06	0.00022663	cellular homeostasis
35051	0.000010179	0.00025519	cardiac cell differentiation
48731	0.000010225	0.00025519	system development
30001	0.000010862	0.00026626	metal ion transport
2027	0.000010903	0.00026626	regulation of heart rate
30705	0.000011141	0.00026917	cytoskeleton-dependent intracellular transport
3208	0.000011528	0.00027558	cardiac ventricle morphogenesis
30003	0.000012832	0.00030355	cellular cation homeostasis
31034	0.00001414	0.00032767	myosin filament assembly
31033	0.00001414	0.00032767	myosin filament assembly or disassembly
3231	0.000015018	0.00034449	cardiac ventricle development
3205	0.000019887	0.00045163	cardiac chamber development
30154	0.000020353	0.00045757	cell differentiation
55065	0.000020551	0.00045757	metal ion homeostasis
51481	0.000025739	0.0005675	reduction of cytosolic calcium ion concentration
71407	0.000028594	0.00062439	cellular response to organic cyclic substance
51239	0.000031644	0.00068442	regulation of multicellular organismal process
51480	0.000032322	0.00069248	cytosolic calcium ion homeostasis
7044	0.000035428	0.0007458	cell-substrate junction assembly
43462	0.000035467	0.0007458	regulation of ATPase activity
7519	0.000038876	0.00080998	skeletal muscle tissue development
32502	0.000044938	0.00092776	developmental process
3206	0.000047171	0.0009651	cardiac chamber morphogenesis
51289	0.000048752	0.00097978	protein homotetramerization
1756	0.000048752	0.00097978	somitogenesis
60538	0.000050798	0.0010119	skeletal muscle organ development
51926	0.000053136	0.0010314	negative regulation of calcium ion transport
14866	0.000053136	0.0010314	skeletal myofibril assembly
14888	0.000053136	0.0010314	striated muscle adaptation
42592	0.000060015	0.001155	homeostatic process
71286	0.000063454	0.0012081	cellular response to magnesium ion
14070	0.000063836	0.0012081	response to organic cyclic substance

9887	0.000069193	0.0012987	organ morphogenesis
43500	0.000075624	0.0014077	muscle adaptation
48468	0.000081962	0.0015133	cell development
9653	0.000084816	0.0015534	anatomical structure morphogenesis
48729	0.000092532	0.0016811	tissue morphogenesis
51155	0.00010432	0.0018802	positive regulation of striated muscle cell differentiation
61053	0.00011123	0.001989	somite development
48869	0.00011692	0.0020743	cellular developmental process
48513	0.00017445	0.0030712	organ development
10880	0.00018415	0.003217	regulation of release of sequestered calcium ion into cytosol by sarcoplasmic reticulum
6812	0.00019449	0.0033716	cation transport
10831	0.00021597	0.0037157	positive regulation of myotube differentiation
35282	0.00022723	0.0038799	segmentation
6811	0.00027194	0.0046088	ion transport
43502	0.00029665	0.0049903	regulation of muscle adaptation
7275	0.00030512	0.0050951	multicellular organismal development
22617	0.00032596	0.0054034	extracellular matrix disassembly
48630	0.00035073	0.0056893	skeletal muscle tissue growth
14878	0.00035073	0.0056893	response to electrical stimulus involved in regulation of muscle adaptation
7522	0.00035073	0.0056893	visceral muscle development
51260	0.00037914	0.0061065	protein homooligomerization
48741	0.00044941	0.0071874	skeletal muscle fiber development
32989	0.0004811	0.0076404	cellular component morphogenesis
51591	0.00049777	0.0078502	response to cAMP
46716	0.00050409	0.0078951	muscle cell homeostasis
30198	0.00051156	0.0079572	extracellular matrix organization
51259	0.00066705	0.010305	protein oligomerization
60306	0.00071018	0.010806	regulation of membrane repolarization
32026	0.00071018	0.010806	response to magnesium ion
7015	0.00071851	0.010806	actin filament organization
34220	0.00071851	0.010806	ion transmembrane transport
48646	0.0007359	0.010995	anatomical structure formation involved in morphogenesis
51049	0.00080479	0.011946	regulation of transport
32879	0.00081239	0.01198	regulation of localization
14823	0.00095564	0.013842	response to activity
43931	0.00096301	0.013842	ossification involved in bone maturation
70977	0.00096301	0.013842	bone maturation
31581	0.00096301	0.013842	hemidesmosome assembly
43056	0.0010391	0.014567	forward locomotion
14889	0.0010391	0.014567	muscle atrophy
14891	0.0010391	0.014567	striated muscle atrophy
42511	0.0010391	0.014567	positive regulation of tyrosine phosphorylation of Stat1 protein
65008	0.0010619	0.014795	regulation of biological quality
51209	0.0011211	0.01543	release of sequestered calcium ion into cytosol
51283	0.0011211	0.01543	negative regulation of sequestering of calcium ion
46034	0.0011804	0.016149	ATP metabolic process
51262	0.0012232	0.016621	protein tetramerization

51051	0.00123	0.016621	negative regulation of transport
51592	0.0012369	0.016621	response to calcium ion
71318	0.0012663	0.016916	cellular response to ATP
51282	0.0013056	0.017238	regulation of sequestering of calcium ion
16266	0.0013056	0.017238	O-glycan processing
43062	0.0013955	0.018319	extracellular structure organization
32956	0.0014422	0.018823	regulation of actin cytoskeleton organization
70588	0.0014768	0.019165	calcium ion transmembrane transport
60420	0.0015103	0.019488	regulation of heart growth
42391	0.0018056	0.023167	regulation of membrane potential
6006	0.0018919	0.024138	glucose metabolic process
46033	0.0020378	0.024927	AMP metabolic process
61050	0.0020525	0.024927	regulation of cell growth involved in cardiac muscle cell development
61056	0.0020525	0.024927	sclerotome development
60316	0.0020525	0.024927	positive regulation of ryanodine-sensitive calcium-release channel activity
51725	0.0020525	0.024927	protein amino acid de-ADP-ribosylation
33058	0.0020525	0.024927	directional locomotion
14874	0.0020525	0.024927	response to stimulus involved in regulation of muscle adaptation
45091	0.0020525	0.024927	regulation of retroviral genome replication
42510	0.0020525	0.024927	regulation of tyrosine phosphorylation of Stat1 protein
50727	0.0020934	0.025237	regulation of inflammatory response
55086	0.0021003	0.025237	nucleobase, nucleoside and nucleotide metabolic process
60348	0.0021316	0.025478	bone development
71277	0.002257	0.026836	cellular response to calcium ion
32844	0.0022889	0.02697	regulation of homeostatic process
9150	0.002292	0.02697	purine ribonucleotide metabolic process
1501	0.0024206	0.02816	skeletal system development
33500	0.0024427	0.02816	carbohydrate homeostasis
42593	0.0024427	0.02816	glucose homeostasis
7204	0.0024427	0.02816	elevation of cytosolic calcium ion concentration
22607	0.0024858	0.028511	cellular component assembly
10830	0.0025123	0.028527	regulation of myotube differentiation
10611	0.0025123	0.028527	regulation of cardiac muscle hypertrophy
60402	0.0025537	0.02871	calcium ion transport into cytosol
60401	0.0025537	0.02871	cytosolic calcium ion transport
14743	0.0030495	0.034115	regulation of muscle hypertrophy
34330	0.0031092	0.034612	cell junction organization
1503	0.0033693	0.036192	ossification
43267	0.0033786	0.036192	negative regulation of potassium ion transport
44208	0.0033786	0.036192	'de novo' AMP biosynthetic process
2318	0.0033786	0.036192	myeloid progenitor cell differentiation
14898	0.0033786	0.036192	cardiac muscle hypertrophy in response to stress
14887	0.0033786	0.036192	cardiac muscle adaptation
10256	0.0033786	0.036192	endomembrane system organization
3299	0.0033786	0.036192	muscle hypertrophy in response to stress
6163	0.0035483	0.037832	purine nucleotide metabolic process
34329	0.0035895	0.037875	cell junction assembly

51279	0.0036024	0.037875	regulation of release of sequestered calcium ion into cytosol
51149	0.0036024	0.037875	positive regulation of muscle cell differentiation
43266	0.0036521	0.038221	regulation of potassium ion transport
51241	0.0037623	0.039194	negative regulation of multicellular organismal process
9259	0.0038009	0.039415	ribonucleotide metabolic process
71347	0.0043224	0.044417	cellular response to interleukin-1
45932	0.0043224	0.044417	negative regulation of muscle contraction
71310	0.0045465	0.046509	cellular response to organic substance
44262	0.0046635	0.047492	cellular carbohydrate metabolic process
