# Supplementary material for: Identification of regenerative roadblocks via repeat deployment of limb regeneration in axolotls
Source: NPJ Regen Med. 2017 Nov 6;2:30. doi: 10.1038/s41536-017-0034-z (PMC5677943; doi:10.1038/s41536-017-0034-z)
Supplement: Supplementary file 11 — Supplementary Table 3 [file 41536_2017_34_MOESM11_ESM.pdf]

Morphological outcomes following *areg* misexpression (8 weeks post-amputation)

Forelimb specimen (limb)	Classification	Description
pCAG-*egfp* control specimen 35	Mild	Carpal fusion
pCAG-*egfp* control specimen 37	Mild	Carpal fusions; truncation in digit 4
pCAG-*egfp* control specimen 19	Mild	Extra digit; carpal fusion
pCAG-*egfp* control specimen 38	Mild	Extra digit; truncation in digit 4
pCAG-*egfp* control specimen 13	Mild	Truncation in digit 4
pCAG-*egfp* control specimen 22	Mild	Truncation in digit 4
pCAG-*egfp* control specimen 24	Mild	Truncation in digit 4
pCAG-*egfp* control specimen 29	Mild	Truncation in digit 4
pCAG-*egfp* control specimen 32	Mild	Truncation in digit 4
pCAG-*egfp* control specimen 1	Normal	
pCAG-*egfp* control specimen 2	Normal	
pCAG-*egfp* control specimen 3	Normal	
pCAG-*egfp* control specimen 4	Normal	
pCAG-*egfp* control specimen 5	Normal	
pCAG-*egfp* control specimen 6	Normal	
pCAG-*egfp* control specimen 7	Normal	
pCAG-*egfp* control specimen 8	Normal	
pCAG-*egfp* control specimen 9	Normal	
pCAG-*egfp* control specimen 10	Normal	
pCAG-*egfp* control specimen 11	Normal	
pCAG-*egfp* control specimen 12	Normal	
pCAG-*egfp* control specimen 14	Normal	
pCAG-*egfp* control specimen 15	Normal	
pCAG-*egfp* control specimen 16	Normal	
pCAG-*egfp* control specimen 17	Normal	
pCAG-*egfp* control specimen 18	Normal	
pCAG-*egfp* control specimen 20	Normal	
pCAG-*egfp* control specimen 21	Normal	
pCAG-*egfp* control specimen 23	Normal	
pCAG-*egfp* control specimen 25	Normal	
pCAG-*egfp* control specimen 26	Normal	
pCAG-*egfp* control specimen 27	Normal	
pCAG-*egfp* control specimen 28	Normal	
pCAG-*egfp* control specimen 30	Normal	
pCAG-*egfp* control specimen 31	Normal	
pCAG-*egfp* control specimen 33	Normal	
pCAG-*egfp* control specimen 34	Normal	
pCAG-*egfp* control specimen 36	Normal	
pCAG-*egfp* control specimen 39	Normal	
pCAG-*egfp* control specimen 40	Normal	
pCAG-*egfp* control specimen 41	Normal	
pCAG-*egfp* control specimen 42	Normal	
pCAG-*egfp* control specimen 43	Normal	
pCAG-*egfp* control specimen 44	Normal	
pCAG-*egfp* control specimen 45	Normal	

pCAG-*egfp* control specimen 46	Normal	
pCAG-*egfp* control specimen 47	Normal	
pCAG-*egfp* control specimen 48	Normal	
pCAG-*egfp* + pCAG-*areg* specimen 7	Mild	Carpal fusion
pCAG-*egfp* + pCAG-*areg* specimen 19	Mild	Carpal fusion
pCAG-*egfp* + pCAG-*areg* specimen 29	Mild	Carpal fusion
pCAG-*egfp* + pCAG-*areg* specimen 41	Mild	Carpal fusion
pCAG-*egfp* + pCAG-*areg* specimen 14	Mild	Digital outgrowth
pCAG-*egfp* + pCAG-*areg* specimen 4	Mild	Truncation in digit 1; carpal fusion
pCAG-*egfp* + pCAG-*areg* specimen 35	Mild	Truncation in digit 1; carpal fusion
pCAG-*egfp* + pCAG-*areg* specimen 18	Moderate	Fusion of radius and ulna
pCAG-*egfp* + pCAG-*areg* specimen 21	Moderate	Fusion of radius and ulna
pCAG-*egfp* + pCAG-*areg* specimen 30	Moderate	Fusion of radius and ulna; digital outgrowth
pCAG-*egfp* + pCAG-*areg* specimen 9	Moderate	Fusion of radius and ulna; truncation in digit 4
pCAG-*egfp* + pCAG-*areg* specimen 31	Moderate	Fusion of radius and ulna; truncation in digit 4
pCAG-*egfp* + pCAG-*areg* specimen 44	Severe	Loss of digit 4 and carpal element
pCAG-*egfp* + pCAG-*areg* specimen 38	Severe	Loss of ulna; loss of digits 1 - 3; loss of several carpals
pCAG-*egfp* + pCAG-*areg* specimen 46	Severe	Near-complete loss of radius and ulna; carpal fusion and loss; truncation in digit 1
pCAG-*egfp* + pCAG-*areg* specimen 42	Severe	Spike
pCAG-*egfp* + pCAG-*areg* specimen 34	No regeneration	Stump; little to no regeneration
pCAG-*egfp* + pCAG-*areg* specimen 36	No regeneration	Stump; little to no regeneration
pCAG-*egfp* + pCAG-*areg* specimen 40	No regeneration	Stump; little to no regeneration
pCAG-*egfp* + pCAG-*areg* specimen 1	Normal	
pCAG-*egfp* + pCAG-*areg* specimen 2	Normal	
pCAG-*egfp* + pCAG-*areg* specimen 3	Normal	
pCAG-*egfp* + pCAG-*areg* specimen 5	Normal	
pCAG-*egfp* + pCAG-*areg* specimen 6	Normal	
pCAG-*egfp* + pCAG-*areg* specimen 8	Normal	
pCAG-*egfp* + pCAG-*areg* specimen 10	Normal	
pCAG-*egfp* + pCAG-*areg* specimen 11	Normal	
pCAG-*egfp* + pCAG-*areg* specimen 12	Normal	
pCAG-*egfp* + pCAG-*areg* specimen 13	Normal	
pCAG-*egfp* + pCAG-*areg* specimen 15	Normal	
pCAG-*egfp* + pCAG-*areg* specimen 16	Normal	
pCAG-*egfp* + pCAG-*areg* specimen 17	Normal	
pCAG-*egfp* + pCAG-*areg* specimen 20	Normal	
pCAG-*egfp* + pCAG-*areg* specimen 22	Normal	
pCAG-*egfp* + pCAG-*areg* specimen 23	Normal	
pCAG-*egfp* + pCAG-*areg* specimen 24	Normal	
pCAG-*egfp* + pCAG-*areg* specimen 25	Normal	
pCAG-*egfp* + pCAG-*areg* specimen 26	Normal	
pCAG-*egfp* + pCAG-*areg* specimen 27	Normal	
pCAG-*egfp* + pCAG-*areg* specimen 28	Normal	
pCAG-*egfp* + pCAG-*areg* specimen 32	Normal	
pCAG-*egfp* + pCAG-*areg* specimen 33	Normal	
pCAG-*egfp* + pCAG-*areg* specimen 37	Normal	
pCAG-*egfp* + pCAG-*areg* specimen 39	Normal	

pCAG-*egfp* + pCAG-*areg* specimen 43	Normal	
pCAG-*egfp* + pCAG-*areg* specimen 45	Normal	
